# Cross platform analysis of transcriptomic data identifies ageing has distinct and opposite effects on tendon in males and females

**DOI:** 10.1038/s41598-017-14650-z

**Published:** 2017-10-31

**Authors:** Louise I. Pease, Peter D. Clegg, Carole J. Proctor, Daryl J. Shanley, Simon J. Cockell, Mandy J. Peffers

**Affiliations:** 1 MRC – Arthritis Research UK Centre for Integrated research into Musculoskeletal Ageing (CIMA), Liverpool, UK; 2 Department of Musculoskeletal Biology, Institute of Ageing and Chronic Disease, The University of Liverpool, Leahurst Campus, Neston, CH64 7TE UK; 3 Institute of Cellular Medicine, Newcastle University, Newcastle, NE2 4HH UK; 4 Institute for Cell and Molecular Biosciences, Newcastle University, Newcastle, NE1 7RU UK; 5 Faculty of Medical Sciences, Bioinformatics Support Unit, Framlington Place, Newcastle University, Newcastle, NE2 4HH UK

## Abstract

The development of tendinopathy is influenced by a variety of factors including age, gender, sex hormones and diabetes status. Cross platform comparative analysis of transcriptomic data elucidated the connections between these entities in the context of ageing. Tissue-engineered tendons differentiated from bone marrow derived mesenchymal stem cells from young (20–24 years) and old (54–70 years) donors were assayed using ribonucleic acid sequencing (RNA-seq). Extension of the experiment to microarray and RNA-seq data from tendon identified gender specific gene expression changes highlighting disparity with existing literature and published pathways. Separation of RNA-seq data by sex revealed underlying negative binomial distributions which increased statistical power. Sex specific *de novo* transcriptome assemblies generated fewer larger transcripts that contained miRNAs, lincRNAs and snoRNAs. The results identify that in old males decreased expression of CRABP2 leads to cell proliferation, whereas in old females it leads to cellular senescence. In conjunction with existing literature the results explain gender disparity in the development and types of degenerative diseases as well as highlighting a wide range of considerations for the analysis of transcriptomic data. Wider implications are that degenerative diseases may need to be treated differently in males and females because alternative mechanisms may be involved.

## Introduction

Tendinopathy is especially common among athletes and manual workers, and development of tendinopathy is known to increase in frequency with age^1^. Tendons provide physical and mechanical connections between muscle and bone allowing movement of the skeletal system^2^. The cellular and molecular mechanisms that result in increased age-related tendon injury are not well established but are thought to result in altered matrix turnover^3,4^. Interest in mesenchymal stem cells (MSCs) as a therapy for musculoskeletal disorders such as tendinopathy and arthritis has increased recently due to their ability to differentiate into osteoblasts for bone regeneration, chondrocytes for cartilage regeneration, and tissue-engineered tendons (TET) for tendon production^5^. Aged MSCs are thought to be less able to proliferate and regenerate tissues giving rise to musculoskeletal diseases affecting 1.7 billion people across the globe^6^. One theory is that as we age genes become hypermethylated blocking transcriptional start sites, which results in reduced transcription and a subsequent reduction in cell proliferation, however contradictory results have been obtained^7–9^. Studies show high concentrations of external glucose reduce the potential for cells to respond to oxidative stress damaging the biomechanics of tendon, and that thyroid disorders and diabetes increase calcification of tendon^10^. Females, diabetics and those treated with quinolones are most susceptible; the development of tendinopathy has been specifically linked to gender and hormones^10,11^. Testosterone, oestrogen, insulin and growth hormones have all been found to influence the development of tendinopathies. In mixed male (n = 3) and female (n = 5) samples from donors with tibial tendon dysfunction oestrogen receptors are not significantly differentially expressed^12^. In male rat achilles tendon systematic application of glucocorticoids decreases tensile strength, collagen type I and lysyl oxidase expression^13^. Diabetic male rats with injured tendon have identified reductions in the expression of collagen I and III as well as MMP-3^14^. Gene focused RT-PCR studies completed on tendon like tissues derived from male mesenchymal stromal cells did not identify significant age-related differences in osteocalcin TGF-*β*2 or decorin^15^. Some gender differences have been highlighted; achilles tendinopathy serum has lower concentrations of both Tumour Necrosis Factor alpha (TNF-*α*), Interleukin 1 beta (IL-1-*β*) and Platelet Derived Growth Factor (PDGF-BB) in females^16^.

Experimental animal models are frequently male which does not represent the demographic affected by tendinitis, additionally they may not capture the structure and function of human tendons, both of which are limitations. There are clear benefits in replacing animal models with alternatives wherever possible; reducing the number of animals used to a minimum^17^. Thus transcriptomic data from TET could prove an invaluable *in vitro* model to assess factors contributing to the development of tendinopathy. RNA-seq has a plethora of advantages over microarrays such as the ability to identify isoforms, alternate splicing, differences in transcriptional start sites, and differential promoter use. Additionally RNA-seq is not dependent on the design of microarray probes, fluorescence of dyes, efficiency of microarray scanners, poly-A selection or RT-PCR steps. RNA sequencing can identify microRNAs, long non-coding RNAs and small nuclear RNAs as well as mRNA resulting in a detailed and comprehensive view of the transcriptome. Transcriptional start sites have been found to be different in males and females producing different isoforms under different oestrogen concentrations and in different tissues^18^. In females hypermethylation of one X chromosome leads to inactivation of transcriptional start sites in some tissues; females are mosaics with patches of tissue expressing either the paternally or maternally derived X chromosome^19,20^.

This study extends the analysis of RNA-seq data from tissue derived Achilles tendon from young and old donors (E-MTAB-2449) to include RNA-seq data from TET (E-MTAB-4879). Both of the aforementioned studies were gender imbalanced. Analysis of these combined datasets was undertaken in order to identify male and female specific gene expression changes with age. Previous work has identified that age affects gene expression in tendon and TET similarly^21^. The validity of this was tested using parallel analysis of microarray data from young and old tendon derived from various tendons^22^ (E-GEOD-26051), contrasting and comparing results. To the authors knowledge this is the first study of its kind, implementing a cross platform parallel analysis of samples with separation of male and female subsets to identify gender specific gene expression changes with age. Separate analysis of male and female data sets simplified analysis and reduced the dependency of results on mathematical algorithms and their underlying assumptions. The results highlight the importance of gender differences which are frequently neglected in gene expression studies.

## Results

### Differentially expressed genes; RNA-seq and microarray

**Figure 1:**
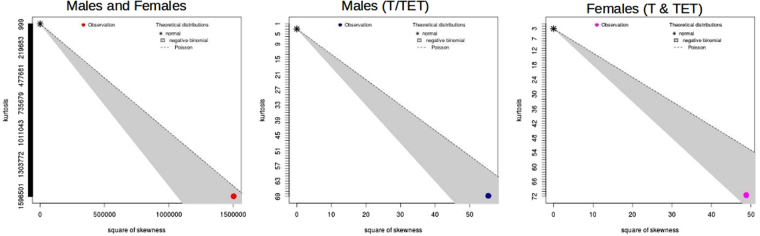
Cullen and Frey graphs of data distribution for all samples assessed together using Method One. Distribution of mixed male and female data sets (red, left), when a mixture of tendon and tissue engineered tendon from male (blue, middle) and young TET versus tendon in females (pink, right) were assessed separately.

**Figure 2:**
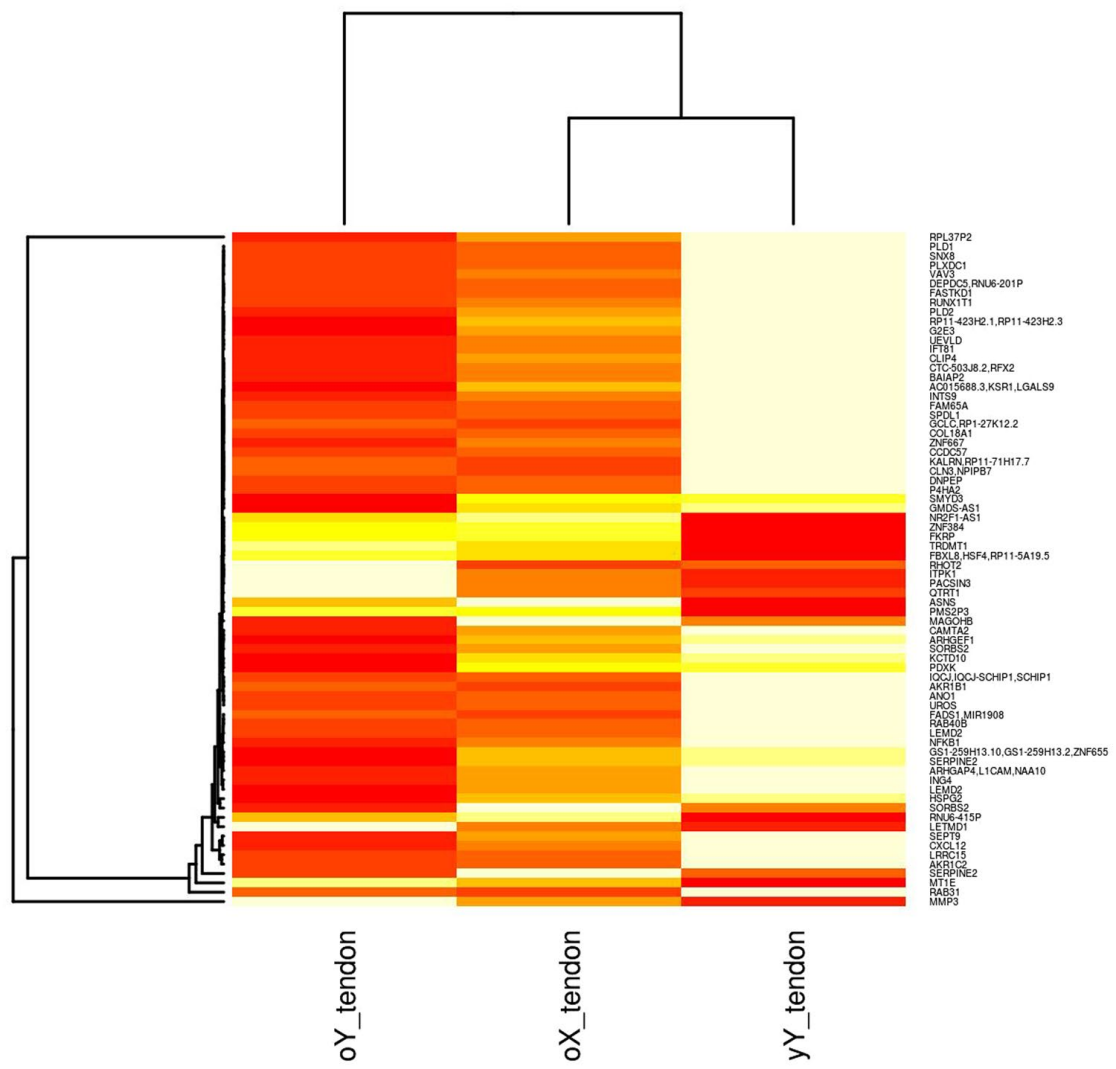
Hierarchically clustered heatmap of mean FPKM normalised transcript counts for genes identified as significant (q < 0.05) in old male tendon (oY tendon) versus young male tendon (yY tendon) that were also identified as DE in old female tendon (oX tendon) versus young female TET. Heatmap colours are white highest expression, yellow-orange medium, and red lowest expression.

**Figure 3:**
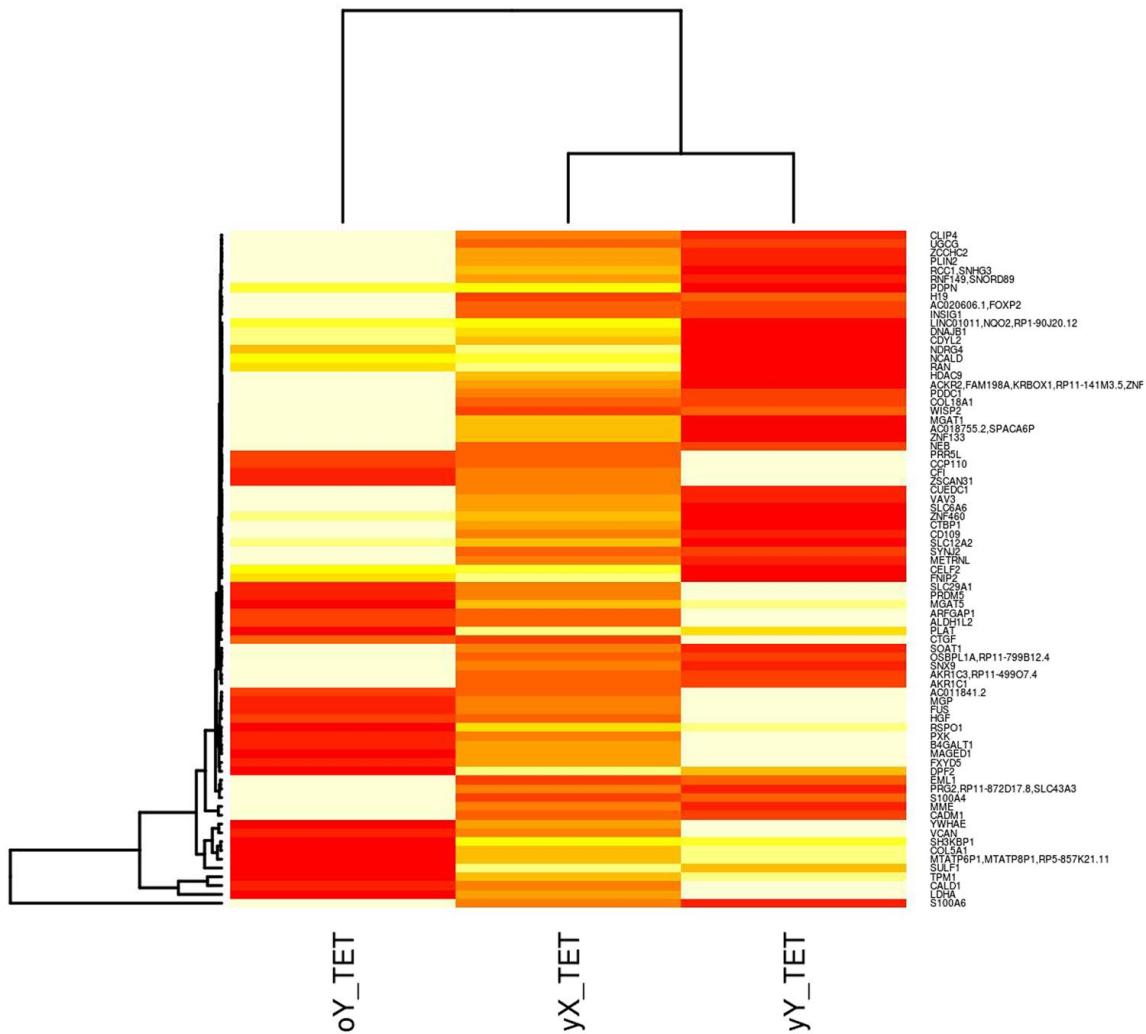
Hierarchically clustered heatmap of mean FPKM normalised transcript counts for genes identified as significant (q < 0.05) in old male (oY) versus young male (yY) tissue engineered tendon (TET) that were also identified as significantly different in young female tissue engineered tendon (yX TET) versus old female tendon. Heatmap colours are white highest expression, yellow-orange medium, and red lowest expression.

**Figure 4:**
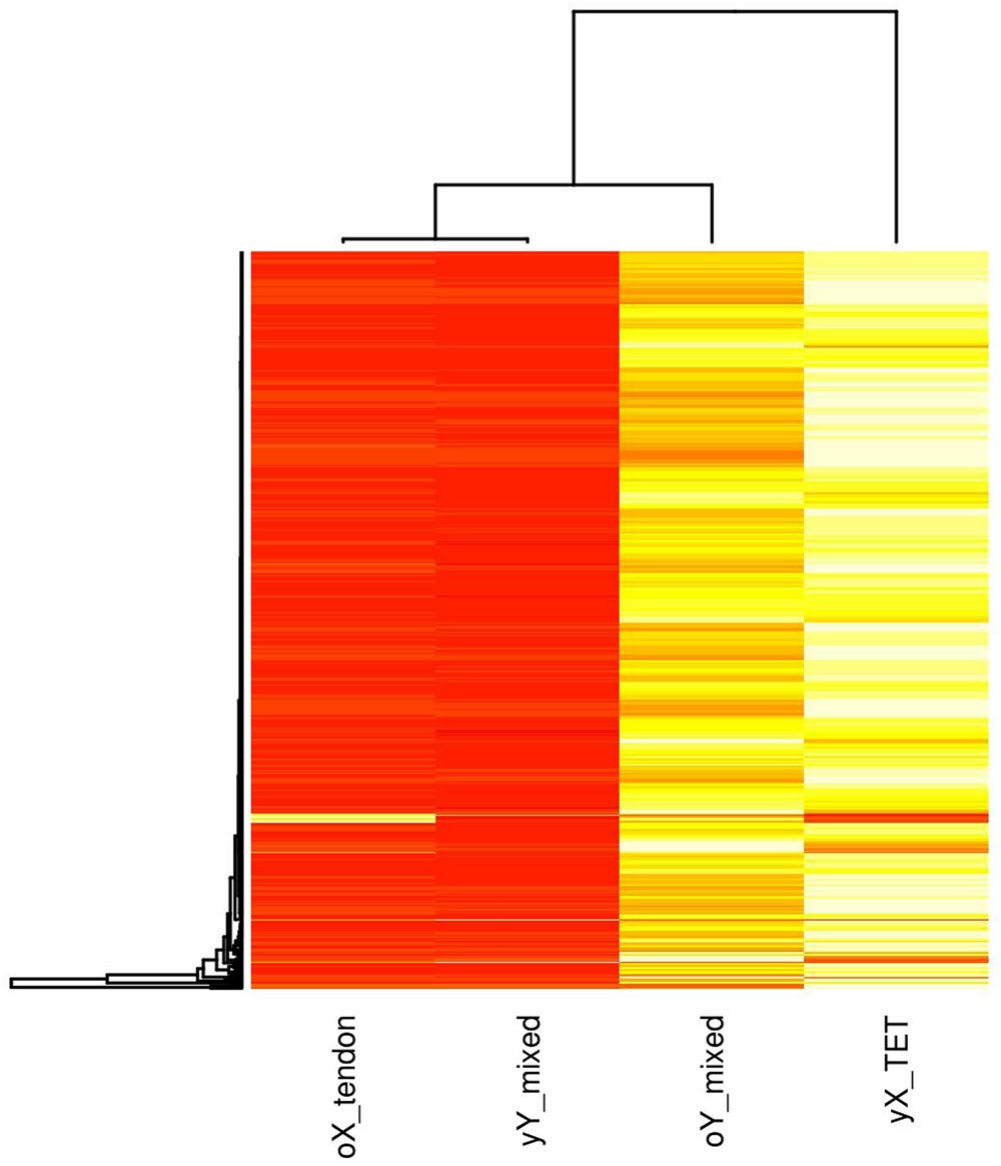
Hierarchically clustered heatmap of mean FPKM normalised transcript counts for genes identified as significant (q < 0.05) in old female tendon (oX tendon) versus young female TET (yX TET) and young and old males assessed using mixed TET and tendon samples (yY mixed and oY mixed respectively). The heatmap represents 696 genes that were significantly (q < 0.05) affected by age in males and females, therefore gene name labels could not be shown. Heatmap colours are white highest expression, yellow-orange medium expression, and red lowest expression.

Cullen Frey graphs of data distribution show that separating samples by gender impacts on data distribution (Fig. 1). The squared cumulative variance CV^2^ of young males and females was similar, however CV^2^ is reduced in old females, but increased in old males. Old females have similar gene expression profiles to young males, and old males are similar to young females (Figure M1S1, Figs 2, 3 and 4). More age-related changes in gene expression were identified in females than males irrespective of platform used (Tables 1 and 2, M1S2, M2S2), despite more male samples being assayed. Without gender separation no genes were identified as significantly affected by age in microarrays, but 51 were up-regulated and 62 down-regulated in RNA-seq (Table M1S4). To assess the impacts of analysis decisions two methods were applied to the data to assess male and female differences in gene expression. The first method assessed significance using a simultaneous analysis of male and female data using a merged gene transcript file. The second analysed males and females in separate pipelines using sex specific merged transcript files. An overview of significantly (q < 0.05) differentially expressed (DE) transcripts, isoforms, promoters, splicing events and transcriptional start sites from RNA-seq data for possible comparisons using Method One are shown in Table 1.

**Table 1:** The number of significant (q < 0.05) transcripts, isoforms, promoters, transcriptional start sites, alternate splicing events other RNAs that were observed in each of the data analysis operations using Method One.

Entity	old (o) V young (y)	female (X) V male (Y)	oY V yY (T /TET)	oX (T) V yX (TET)
Transcripts	111	1,206	159	5,515
Isoforms	74	1,176	0	5,777
Promoters	74	0	0	5,787
Transcriptional start sites	92	1,188	0	5,716
Splicing	0	0	0	0
miRNAs	11,016	11,016	11,016	11,016
lncRNAs	6,120	6,120	6,120	6,120
snoRNAs	342	342	342	342
scaRNAs	294	294	294	294
Total transcripts tested	2,845,471	2,845,471	2,845,471	2,845,471
Percentage DE	0.00004	0.0004	0.00006	0.002
Average transcript size	77,424	77,424	77,424	77,424
Range of transcript sizes	25:877,728	25:877,728	25:877,728	25:877,728

**Table 2:** The number of significant (q < 0.05) transcripts, isoforms, promoters and alternate splicing events that were observed in each of the data analysis operations using Method Two.

Entity	oY V yY (T/TET)	oY V yY (T)	oY V yY (TET)	oX (T) V yX (TET)
Transcripts	1,033	228	683	19,816
Isoforms	116	0	392	14,191
Promoters	0	0	0	1,378
Transcriptional start sites	375	93	616	17,259
Splicing	51	0	0	474
miRNAs	1,476	1,476	1,476	1,477
lncRNAs	817	817	817	800
snoRNAs	24	24	24	22
scaRNAs	0	0	0	0
Total transcripts tested	130,771	130,771	130,771	121,829
Percentage DE	0.008	0.002	0.005	16.2
Average transcript size	121,849	121,849	121,849	116,159
Range of transcript sizes	89:2,298,478	89:2,298,478	89:2,298,478	89:2,298,478

For females (X) only young (y) tendon (T) and old (o) TET was available. For males (Y) young and old cells from T, TET were assessed when both were combined (T/TET). Old versus young, and male versus female comparisons were possible due to CuffDiff analysis on male and female data sets combined. When age and gender factors are used to separate samples into groups all DE transcripts from females have different transcriptional start sites (TSS). More transcripts, isoforms, and transcriptional start sites were identified as significantly different in the male versus female comparison than the old versus young comparison. Additionally old female tendon (oX T) versus young female TET (yX TET) identified the most transcripts as DE. In females all transcripts had different promoters, and transcriptional start sites giving rise to different isoforms. No alternate splicing was identified in any comparison and a large number of small RNAs (miRNA, lncRNA, snoRNA, and scRNA) were identified. The total number of transcripts identified was over 2.5 million, and the percentage of transcripts identified as DE was below 0.01% in all comparisons. Descriptive statistics were obtained for the data generated using Method Two; separate male and female analysis with sex specific transcript files (Table 2).

**Figure 5:**
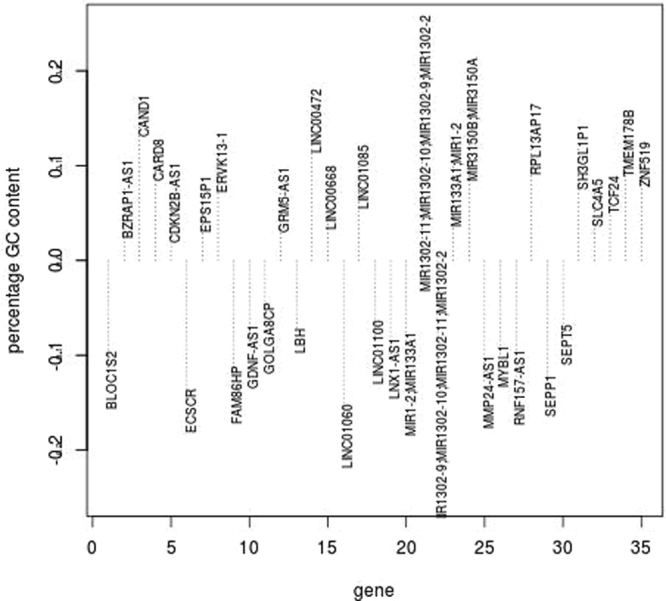
Histogram of percentage difference in GC content for 35 female transcripts compared to male transcripts that were identified as significant (q < 0.05) in old male tendon (oY tendon) versus young male tendon (yY tendon) and also identified as DE in old female tendon (oX tendon) versus young female TET. Negative values indicate the transcript for that gene had a lower GC content in females than the transcript for that gene in males.

The analysis was repeated for males using the E-MTAB-2499 tendon RNA-seq data (old male versus young male (oY v yY (T)) and E-MTAB-4879 TET old male versus young male (oY v yY (TET). Additionally an old versus young analysis was completed on mixed tendon and TET from males (oY V yY (T/TET)). Direct old versus young, and male versus female comparisons were not possible due to separate CuffDiff analysis for male and female data sets and sex specific merged transcript files. However significantly (q < 0.05) DE transcripts identified in males and females were compared (Figs 2, 3, 4 and 5). Combining experimental data sets from tendon and TET increased the number of transcripts identified as DE in males. More isoforms and transcriptional start sites were identified as significant when TET was assessed. No alternate splicing was identified using individual data sets from males, however when they were combined and sex specific transcript files used 51 alternate splicing events were identified. When old female tendon was compared with young female TET 19,816 out of 121,828 transcripts were DE, (16.2%), more than three times as many as when male and female samples were assessed simultaneously by CuffDiff using a mixed sex transcript file (Method One). Sex specific transcriptome assembly identified far fewer transcripts than Method One (Table 1) and both the average and range of transcript sizes are much larger. Interestingly the equivalent of nearly every annotated gene is identified as DE in old females and a lower count of small RNAs (miRNA, lncRNA, and snoRNA) was returned than when Method One was used. In old female tendon nearly all of the transcripts identified had different transcriptional start sites and promoters, once again the majority (but not all) were isoforms of genes, only 474 alternate splicing events were identified. Separation of samples by gender identified the correct data distribution to assess significance was the negative binomial. When separated by gender standard deviation, skewness and kurtosis were reduced in male and female subgroups (Fig. 1). This increased statistical power allowing for the identification of more significantly DE genes. Separation of samples by sex prior to CuffMerge and CuffDiff analysis also reduced the total number of transcripts tested and further increased the number of transcripts identified as significantly (q < 0.05) DE (Table 2). Cullen Frey graphs of data distributions plotted for data generated using Method Two showed that male subsets of data followed a negative binomial distribution when tendon and TET were assessed separately, as well as when they were mixed (Figure M2S1). Young female TET and old female tendon assessed separately also followed a negative binomial distribution (data not shown). Separating male and female data sets and transcript files prior to CuffDiff analysis increased skew, kurtosis and standard deviation estimates (Figure M2S1), however the number of transcripts identified as significantly different remained higher in males and females.

No genes were identified as significantly DE using both technologies for aged males or females (Tables M1S3 and M2S3). However in aged males and females many processes, gene ontology categories and pathways identified using each technology were similar and related. Microarray results can be influenced by the sensitivity of normalisation methods, and like RNA-seq could be affected by combined normalisation and analysis of male and female data sets. For these reasons a variety of available normalisation methods were assessed for microarray data (E-GEOD-26051) with male and female data sets normalised together, and with sex specific normalisation. Sex specific normalisations reduced the number of significant genes identified to zero for each method apart from Guanine Cytosine Robust Multi Array (GC-RMA) where 19 genes were identified as significantly (q < 0.05) increased in males. The number of genes identified as significant for males and females normalised together using different normalisation methods are shown in Table 3.

**Table 3:** The number of genes identified as significantly (q < 0.05) increased and decreased in expression in aged males and females using different normalisation approaches.

Normalisation Method	Male up	Male down	Female up	Female down
RMA	280	0	1503	983
MAS5	0	0	0	0
GC-RMA	131	1	0	0
VSN	0	0	0	0
VSN-RMA	19	0	0	0

Table 3 shows that significantly (q < 0.05) DE genes were identified in both males and females using Robust Multi-Array (RMA) normalisation. However if other normalisation methods were used the number of genes DE in old females was reduced to 0, in males they were also reduced but some were still identified as significant. Significant genes were identified in males for all normalisation methods except Variance Stabilsing Normalisation (VSN) and Micro-Array Suite 5 (MAS5). For the female data set significantly DE genes were only identified using RMA. Methods that correct for mismatches (MAS5) high variance (VSN and VSN-RMA) and high GC content (GC-RMA) of probes reduced the number of significant results by reducing the sensitivity of microarrays. RMA normalisation was most appropriate for the data taking into consideration that female transcripts were different to male transcripts (Tables 1 and 2), and therefore would be classed as mismatches. RMA normalisation does not control for mis-matches or the GC content of probes which also differed in female transcripts (Fig. 5). More genes were affected by ageing in females irrespective of the technology used (Tables 1, 2, 3, M2S3 and M2S2). Furthermore assessing males and females in separate CuffDiff analysis with sex specific merged transcript files identified even more DE genes. The main focus of this report was the gender and age analyses because they addressed confounding factors, and raised important biological questions. In addition summary statistics (Tables 1, 2, 3, Figs 1 and M2S1) strongly indicated these were going to be the most informative, interesting and reliable analyses.

### Gene Ontology and pathways overview

#### Males increased

Using Method One with RNA-seq data genes in the p38MAPK cascade were over-represented in old males (Figure M1S2). Using Method Two biological processes increased in old males included cellular response to stress, replicative senescence, regulation of fibroblast proliferation, metabolism, cellular metabolism, co-enzyme metabolism and protein localisation (Figure M2S2). Significant cellular components were of nuclear origin (Figure M2S3), and molecular functions included ion binding, catalytic activity and transferase activity (Figure M2S4). In microarrays only metallopeptidases (MMPs) and ammonia assimilation genes were also increased (Figure M1S3).

#### Males decreased

Gene Ontology analysis of RNA-seq genes decreased in old males using Method One identified they were involved in regulation of the immune system with reductions in collagen fibril organisation, keratin catabolism and mineral regulation (Figure M1S7). Using Method Two biological processes increased included protein galactysolation and glycosylation, as well as negative regulation of the lectin pathway (Figure M2S10). Molecular function and cellular component categories identified reductions in collagen type XI, interleukin-8 and tumor necrosis factor binding as well as a reduction in androgen binding activity (Figures M2S11 and M2S12).

#### Male Pathways

Only the nitrogen metabolism pathway (glutamate synthesis) was identified as increased in old males using microarrays. RNA-seq identified more pathways; when mixed tendon and TET were assessed using Method One genes increased were involved in inflammation (cytokines), calcium signalling, response to hormones, Janus kinase (JAK) Signal Transducer and Activator of Transcription (JAK-STAT) signalling, increased cell cycle and cancers. Genes decreased were involved in immune signalling; major histocompatibility complex II, killer cell inhibitory receptor, natural killer cells and regulation of cell proliferation (Table M1S4). When Method Two was used with mixed tendon tissues pathway results were consistent with increased cell cycle. Increased expression of genes in cancer pathways, ABC transporters, ubiquitin mediated proteolysis, PI3k-AKT signalling, and focal adhesion were identified. No pathways were identified by assessing genes significantly decreased in expression. When tendon tissue was assessed separately, pathways containing genes increased in expression in old males included prostate cancer, MAPK signalling, neurotrophin signalling, leukemia and those associated with infections. Those decreased included Ras signalling, pathways in cancer, ether lipid metabolism and cytokine-cytokine receptor pathways. Separate analysis of TET identified increases in glycosphingolipid biosynthesis, RIG-I like receptor signalling, fc*γ* R-mediated phagocytosis, pathways in cancer, PPAR signalling and regulation of the actin cytoskeleton consistent with increased cell cycle. Significant pathways identified by genes significantly decreased in expression included transcriptional misregulation in cancer, N-glycan biosynthesis, proteoglycans in cancer, and PI3K-AKT signalling (Table M2S5).

#### Females increased

RNA-seq identified increased gene expression in old females that represented a variety of biological processes involved in cell development, response to oxygen, lipid transport, protein modification, T-cell anergy as well as alterations in metabolic pathways (Figures M1S4 and M1S5). The most prevalent molecular functions affected were heterocyclic compound binding and increased ubiquitin activity (Figure M1S6). Results from Method Two, were consistent with Method One, biological processes included negative regulation of macromolecule metabolism, protein localisation, cellular metabolism, and anatomical structure morphogenesis (Figure M2S4). Significant cellular components were the extracellular exosome and cytosol (Figure M2S5). The most prevalent molecular functions were enzyme binding and regulation of nucleic acid binding, and transcription factor activity (Figure M2S6).

#### Females decreased

RNA-seq identified genes decreased in expression in old females using Method One were involved in cellular organisation, development and adhesion, response to chemical and ADP metabolism (Figure M1S8). On the other hand microarrays identified decreases in transcription, chromatin organisation, nucleic acid binding, compound cyclic binding and metabolism (Figures M1S9 and M1S10). Method Two identified reductions in protein modification, cell cycle regulation, response to stress, macromolecule metabolism, cellular metabolism, organelle and cellular organisation, as well as nitrogen metabolism (Figure M2S7). These results were affirmed by network topology which identified high connectivity with histone de-acetylases, cell cycle genes and cullin encoding genes involved in ubiquitination (Table 8). Cellular components included the nucleus, catalytic complexes, and adherens junction (Figure M2S8). Molecular functions significantly reduced included zinc ion binding, enzyme binding, ATPase activity, kinase activity, catalytic activity and binding (Figure M2S9).

#### Female Pathways

Contrasting Gene Ontologies and genes were affirmed by pathways in which changes in old females were the opposite of those seen in old males. A reduction in the cell cycle was evidenced by diminution in the expression of cyclinD, DNA replication, RNA transport, metabolism, focal adhesion and increased expression of TGF-*β* (Table M1S5). These results were confirmed using Method Two which identified reductions in MAPK signalling, cancer and the P53 pathway, JAK-STAT signalling, focal adhesion, lipid metabolism, and oxidative phosphorylation (Table M2S4). Microarrays identified increased lipid metabolism, alcohol dehydrogenase and retinol metabolism that were mirrored by fatty acid degradation as well as reductions in glycolysis and steroid biosynthesis in RNA-seq. Coupled with increased ubiquitin mediated proteolysis and insulin resistance the data indicates increased autophagy occurs in old female cells (Figure M1S11). Most notably increased expression of genes in pathways in old males were mirrored by decreases in genes in the same pathways in old females (Table 4, detailed in Table M2S4).

**Table 4:** Pathways whose genes changed in different directions in males and females.

Pathway	Direction in males	Direction in females
neuroactive ligand receptor	increased	decreased
focal adhesion	increased	decreased
JAK-stat signalling	increased	decreased
p53 signalling	increased	decreased
natural killer cell mediated cytotoxicity	increased	decreased
pathways in cancer	increased	decreased
cytokine-cytokine receptor interaction	increased	decreased
Leishmaniasis	decreased	increased
Toxoplasmosis	decreased	increased
Asthma	decreased	increased

### Sex specific gene expression changes

The processes and pathways affected in old males and females were different irrespective of the technology and methods used to assess them. Summary data from both technologies indicated gender differences in cell proliferation, metabolism, mineral handling, immune signalling and oxidative stress. To assess the impact of sex separation on gene expression the log_2_ fold change in expression of genes identified as significantly affected by age in the mixed sex tendon study by Peffers *et al*.^55^ E-MTAB-2449 that were re-analysed with sex separation in this study are shown in Table 5.

**Table 5:** log_2_ fold change of transcripts identified as significantly (q < 0.05) DE in tendon by Peffers *et al*.^55^ in this study.

gene	Peffers tendon mixed sex	tendon male	TET male	T/TET male	TET vs T females
ANO1	−1.04	−8.28	NA	NA	−6.11
EMCN	−1.09	−6.76	NA	NA	NA
LRRC15	−3.13	−3.89	NA	NA	2.19
MXRA5	−3.13	−4.59	NA	−2.14	NA
PIEZO2	−2.45	−6.60	NA	−3.91	NA
SLC50A1	−1.53	NA	NA	2.4	−2.72
DOCK5	1.01	NA	NA	5.08	−3.50
MCTP2	2.08	NA	NA	5.56	−6.90
PRDM8	1.39	NA	NA	5.22	NA
SATB	1.13	NA	NA	6.80	−5.66
UCHL1	1.98	NA	NA	6.05	−4.69
RSP01	2.83	NA	−2.31	NA	−11.60
SLC6A15	4.97	NA	5.80	NA	NA
CPE	1.15	NA	2.02	NA	NA
CRABP2	−4.36	NA	−2.48	NA	−3.0
SDC1	−3.50	NA	−2.85	NA	NA

Table 5 shows the log_2_ fold change of some transcripts identified as significantly (q < 0.05) DE in tendon in the original study by Peffers *et al*.^55^ compared to the log_2_ fold change in expression of significantly (q < 0.05) DE transcripts identified in tendon and TET in this study. When no significant (q < 0.05) DE was identified for a gene in the analysis it is shown as NA. For most of the genes shown in Table 5 the fold change in expression is greater when males are considered separately from females. Some of the genes (LRRC15, DOCK5, MCTP2, SATB, UCHL1) changed in different directions in old males and females. Some genes identified as significantly affected by age in old tendon by Peffers *et al*.^55^ were not significant when old male tendon was analysed separately from old female tendon. To visualise differences in the expression levels heatmaps were generated from RNA-seq data to compare and contrast the expression levels of genes affected by age in males and females for separate and mixed tendon and TET tissues (Figs 2, 3 and 4).

Figure 2 shows that tendon from young males (yY tendon) had different gene expression to tendon from old males (oY tendon), and old female tendon (oX tendon) was different to both male samples. Hierarchical clustering of samples identified old female tendon (oX tendon) clustered with young male tendon (yY tendon) and that the expression levels of genes in old male and old female tendon are not the same. Most of the genes are tightly clustered, however matrix metalloproteinase-3 (MMP3) clustered separately from the rest of the genes, and was increased in expression in old male tendon, but had lower expression levels in old female tendon. Only four genes had similar expression levels in old tendon derived from males and females. They included a transcription factor (zinc nuclear factor 384 (ZNF384)) that regulates the expression of MMPs, a gene involved in protein glycosylation (fukutin related protein (FKRP)), the pseudogene Postmeiotic Segregation Increased 2-Like 3 (PMS2P3) and regulator of the immune system and class I MHC presentation, F-Box And Leucine Rich Repeat Protein 8 (FBXL8/HSF4). Old female and young male tendon had similar expression levels for the histone methyltransferase SET and MYND Domain Containing 3 (SMYD3) as well as Pyridoxal (Pyridoxine, Vitamin B6) Kinase (PDXK) involved in metabolism.

TET from young males (yY TET) had different gene expression to TET from young females (yX TET) (Fig. 3). Hierarchical clustering of samples identified that young male TET (yY TET) clusters with young female TET (yX TET). Only eight genes had similar expression levels in young female TET and young male TET. They included two regulators of endocytosis, SH3 Domain Containing Kinase Binding Protein 1 (SH3KBP1) and synaptojanin 2 (SYNJ2). Two genes that regulate the synthesis of glycoproteins and glycosylation; UDP-Glucose Ceramide Glucosyltransferase (UGCG) and Cluster of differentiation (CD109), a regulator of cholesterol metabolism, lipogenesis and glucose homeostasis insulin induced gene 1 (INSIG1), a cytoskeleton component Nebulin (NEB), the transcription factor forkhead box protein 2 (FOXP2) and Collagen Type XVIII *α* 1 (COL18A1).

The heatmap in Fig. 4 represents the mean FPKM normalised counts for 696 genes that were identified as significantly (q < 0.05) DE in old tendon tissues. When mixed (tendon tissue + TET) male samples were assessed this increased the number of replicates in young and old age groups resulting in more genes being identified as significantly DE. Hierarchical clustering of samples by gene expression profiles identified that old females (oX) cluster with young males (yY) and the expression levels of genes in young female (yX) TET are most similar to old males (oY) but they cluster separately. The pattern is consistent with the clustering pattern of sample groups in the PCA plot (Figure M1S1), and the contrasting Gene Ontology and Kegg pathways identified for males and females detailed in Method One and Method Two supplementary file.

### Gene expression microarray and RNA-seq consistencies

No genes identified as DE using microarrays were also identified as DE using RNA-seq (Tables M1S3 and M2S3), however affected processes were similar. The sensitivity of microarrays to detect transcripts is determined by their similarity to known protein coding regions. Tables 1 and 2 identified that the majority of transcriptional start sites and promoters were different in old females indicating the sequences and sequence properties could differ from those microarrays are designed to detect. The sequences of female transcripts were interrogated as described in Method Two, sequence properties and the percentage GC content of some transcripts in male and female data sets were different (Fig. 5).

Transcripts in females started and ended at different loci, contained miRNAs, lincRNAs and these were transcribed from the reverse strand. The GC content of genes is different because the transcripts are different. Out of 696 transcripts that were DE in old males and females (Fig. 5) 148 (21.3%) had different GC content in old females compared to old males. Figure 5 shows the transcripts with greater than seven per cent difference in GC content in females. Some transcripts (CAND1, LINC00472, MIR3150A/B) had as high as 15 per cent more GC content in females than in males. Other transcripts (ECSCR, LINC01000) had fifteen per cent lower GC content in old female transcripts compared to male transcripts. DE microRNAs (MIR), long non-coding RNAs (LINC) and snoRNAs were identified as contributing to differences in GC content in the analysis (Table M2S6). To investigate the role of other RNAs these were subset from mRNA transcripts (Table M2S6). This identified that the MIR and LINCs were transcribed with genes. Network analysis completed on significantly (q < 0.05) DE transcripts in males and females identified that the most significant reactome categories related to mRNA splicing and processing (Table 5). These results are supported by KEGG significant pathways which identified that genes increased in expression were involved in RNA transport and non-homologous end joining (Table M2S4). The results are also consistent with the most significant Biological Process category identified by genes increased in old females, negative regulation of macromolecule metabolism. Within this category were RNA splicing, translation, RNA catabolism and gene expression (Figure M2S4). Network analysis was completed on DE genes in males and females, KEGG functional enrichment results can be found in Table M2S7. Briefly the results identified pathways related to immune signalling, cell cycle and metabolism that are consistent with the KEGG pathway and Gene Ontology results obtained for males and females when analysed separately using both methods. The top ten significant (q < 0.05) Reactome categories identified for genes increased and decreased in expression in females (Table 6) identified the cell cycle, immune signalling, glucose metabolism and regulation of cholesterol biosynthesis.

**Table 6:** The most significant (q < 0.05) reactome categories identified for genes decreased in expression in old age in females.

Reactome genes decreased	Expected	Hits	q value
Cell Cycle, Mitotic	75.3	111	0.0065
Class I MHC mediated antigen processing and presentation	48.9	77	0.0065
Cytosolic tRNA aminoacylation	4.4	14	0.0065
Loss of proteins required for interphase microtubule organization from the centrosome	11.9	26	0.00823
Loss of Nlp from mitotic centrosomes	1.9	26	0.00823
Base-free sugar-phosphate removal via the single-nucleotide replacement pathway	1.83	8	0.00823
Glucose metabolism	12.8	27	0.00823
tRNA Aminoacylation	7.69	19	0.00823
p75 NTR receptor-mediated signalling	15.6	31	0.00823
Regulation of Cholesterol Biosynthesis by SREBP (SREBF)	7.14	18	0.00837

For males no significant reactome categories were identified for upregulated genes, whilst genes decreased identified the xenobiotic reactome (q = 0.00217) alone. Significant reactome categories for upregulated genes in old females were mRNA splicing, processing and transport (Table 7).

**Table 7:** The most significant (q < 0.05) reactome categories identified for genes increased in expression in old age in females.

Reactome genes increased	Expected	Hits	q value
Gene Expression	56.1	102	0.000000213
mRNA Splicing	5.94	20	0.000636
mRNA Splicing - Major Pathway	5.94	20	0.000636
Processing of Capped Intron-Containing Pre-mRNA	6.15	20	0.000831
Transport of Mature mRNA derived from an Intron-Containing Transcript	1.34	9	0.000948
mRNA Processing	7.23	21	0.00159
mRNA 3′-end processing	1.86	10	0.00159
Post-Elongation Processing of Intron-Containing pre-mRNA	1.86	10	0.00159
Transport of Mature Transcript to Cytoplasm	1.55	9	0.00201

Network topology diagrams for females identified Ubiquitin C (UBC) was a significant node connected to all genes increased in expression. UBC was increased in expression 6.7 fold in old females (q = 0.0003). In old females genes increased in expression were involved in immune response (TNF Receptor Associated Factor 6 (TRAF6)), growth and transcriptional regulation; Specificity Protein 1 (SP1) and and Early Growth Response 1 (EGR1). Interestingly Cullin Associated and Neddylation Dissociated 1 (CAND1) and Amyloid *β* Protein Precursors (APP) were identified as significant nodes in the networks for genes increased and decreased in expression (Figure M2S14). More significant nodes with high connectivity were identified for genes decreased in expression. Highly connected genes decreased in expression in old females are detailed in Table 8.

**Table 8:** Fold change of genes significantly (q < 0.05) decreased in expression in old females that were highly connected in the network grouped by function.

Function	Genes decreased in old females	log_2_ fold change
Cell cycle regulators	SRC, BRCA1, TP53, RELA, CSNK2A1, MCC, CDK2	−8.6, −3.7, −5, −3.2, −2.9, −3.2, −5
Transcriptional regulators	IKBKG, SMAD3	−4.9, −3.0
Histone De-Acetylation	HDAC2, HDAC5, HDAC1	−6.6, −3.6, −2.6
Amino acid metabolism	YWHAE, YWHAB	−4.2, −4.5
Ubiquitination	CUL3, CUL5, CUL7, CUL9, CUL4B, CAND1, APP	−2.5, −8.5, −2.0, −2.1, −2.8, −4.8, −3.0

Most of the genes decreased are involved in cell cycle regulation (Table 8). Interestingly ten histone de-acetylation genes (HDAC 1, 2, 4, 5, 6, 7, 8, 9, 10 and 11) were at least two fold decreased in expression in old females, which is consistent with the reduction in chromatin re-modelling identified using microarrays. A total of 18 UBE2 genes and five CUL genes involved in ubiquitination were decreased in expression in old females. Consistently gene ontology and KEGG pathways identified cancer and cell cycle regulators that were over-represented by genes decreased in expression. They include; SRC Proto-Oncogene (SRC), Breast Cancer 1 (BRCA1), RELA Proto-Oncogene NFκB Subunit (RELA), Mutated in Colorectal Cancers (MCC), and Cyclin Dependent Kinase 2 (CDK2).

For males analysis was completed on mixed tendon tissues as well as TET and Achilles tendon separately. Network analysis was completed on genes increased in expression in each analysis, the results are summarised in Table 9.

**Table 9:** Genes increased in expression that had high connectivity in network analysis grouped by function.

Tissue	Function	Genes increased in old males
T/TET	Cell cycle regulators	SMARCA4, CDKN1A, TNFRSF1A, PIN1
	Transcriptional regulation	ATXN7, PML
	Growth factors	EGFR, HGS
	Chromatin remodelling	CHD4
	Cell adhesion and signalling	FN1
Tendon	Amino acid metabolism	RHOT2, ASNS, TRDMT1
	Transcriptional regulation	PACSIN3, NFIL3, UBC
	Ubiquitination	UBC
	Autophagy	ATG13
TET	Response to hormones	SGK1, RAN
	Cell cycle regulators	KYNU, BIN1, MITF, LDLP, NEB, CTPB1, EPB41L3
	Stress response	S100A4, DNAJB1, DNAJA1, USP21
	Chromatin remodelling	HDAC9

The processes identified in each of the tissues were different, the mixed analysis and TET both identified increased expression of cell cycle regulating genes. Whilst the genes identified in each analysis were different some processes were similar. Chromatin remodelling was identified in mixed tendon analysis and tendon. Response to cellular stress were evidenced by ubiquitination and autophagy in tendon, and response to stress in old male TET. When old male TET was assessed separately genes increased respond to hormones; Serum/Glucocorticoid Regulated Kinase 1 (SGK1), RAN, Member RAS Oncogene Family, Androgen Receptor-Associated Protein 24 (RAN). The functions of genes identified by network analysis of genes decreased in expression are summarised in Table 10.

**Table 10:** Genes decreased in expression that had high connectivity in network analysis grouped by function.

Tissue	Function	Genes decreased in old males
T/TET	Transcription factor	NRII3
	Protease inhibitor	A2M
Tendon	Transcription regulation	NFKB1, RUNX1T1
	Chromatin remodelling	MECP2, ING4
	Cell cycle regulation	SEPT9, PLD2, RACGAP1
	Insulin responsive	BAIAP2, SORBS2
	Ubiquitination	UBC
	Interleukin signalling	IL1RAP
TET	Transcription regulation	ERG, MAGED1
	mRNA splicing	FUS
	Cell cycle	ERG, PIAS1, SH3KBP1

Most of the genes decreased in expression in male tendon tissues were involved in regulating transcription. More processes were identified in tendon than when tendon and TET are mixed. In mixed tendon/TET NR1I3 a transcription factor that regulates cytochromes was decreased. This coincided with increased metabolism, ion binding and catalytic activity (Figures M2S2 and M2S4). When TET was considered separately regulators of differentiation and proliferation were decreased. FUS RNA Binding Protein that regulates pre-mRNA splicing was also decreased (Table 10) and no alternate splicing was identified in old male TET (Table 2).

Comparative analysis of RNA-seq data identified that 696 genes were significantly (q < 0.05) DE in males and females, but their expression levels changed in opposite directions (Fig. 4). RNA-seq identified one gene CRABP2 that was significantly (q < 0.016) decreased, two and three-fold in old males and old females respectively. No genes were identified as significantly (q < 0.05) DE using both microarrays and RNA-seq.

## Discussion

Analysis of RNA-seq data generated from TET was extended to include RNA-seq and microarray data from young and old tendon providing more samples for each biologically defined group (young and old males and females respectively), to compensate for a gender imbalance in each of the separate experiments. The work is subject to limitations namely; combining experimental data sets is problematic due to batch effects. A batch effect cannot be reliably determined due to the differences in the biological compositions of each experiment (Table 12) which could contribute to differences in sample clustering. Additionally the tendon tissues from each experiment were treated differently, TET was cultured in the absence of sex hormones and treated with TGF-*β*3 to initiate differentiation, whereas RNA was directly extracted from Achilles tendon after donation. TGF-*β* proteins are involved in regulating cell proliferation, differentiation and maturation; high concentrations of TGF-*β*3 have been observed in mature oocytes of large folicles^23^. Young female and most old male samples came from TET (Table 12) that had been treated with TGF-*β*3 to initiate differentiation. High cell cycle in young females and old males could be a consequence of treatment with TGF-*β*3, which could lead to the observed lower cell cycle in old females and young males. Furthermore uncertainty regarding the biological differences identified exists; it is not possible to unconfound potential batch effects, treatment or biological differences, especially for females where no cross-over samples were available in each of the experiments. To try and determine whether differences were biological in nature, as a consequence of a batch effect, or different treatment regimes for tendon and TET, heatmaps of FPKM normalised transcript counts from tendon (Fig. 2) and TET (Fig. 3) were used to show that the global expression of genes differ in males and females within tissue type, experimental data set and age group, indicating the differences are biological and due to sex differences. For males cross-over samples were available, young versus old comparisons of TET identified a more pronounced response to testosterone depletion in TET (cultured in the absence of testosterone) however increases in cell cycle, oxidative stress and alterations in immune signalling were identified in both tendon and TET. Microarray data for males and females was analysed in conjunction with RNA-seq data to determine whether the observed differences were due to differences in TET and tendon, or sex specific gene expression changes with age. However only one method for microarray data normalisation (RMA) identified DE transcripts in males and females (Table 3). It has previously been concluded from microarray data that age and gender do not affect gene expression based on covariance analysis^22^. The results of this study identify that in the context of age, gene expression in males and females violate the underlying assumptions of covariance analysis, notably gene expression moves in opposite directions. An in depth discussion about the statistical considerations and observations made in the analysis can be found in the statistical discussion in the supplementary file. More genes are identified as DE when males and females are separated than when assessed together (Tables 1, 2, M1S3 and M2S4). However no genes were identified as consistently DE using microarrays and RNA-seq for males or females. Different results may have been obtained due to age differences, different tendons used, differences in the sensitivities of technologies, assumed data distribution, mathematical assumptions, methods for processing microarray data as well as dependence of microarrays on probes designed to be specific to known protein coding regions of the genome^24^. Transcripts increased in expression using RNA-seq with females are involved in mRNA splicing, capping, processing and transport reactomes (Table 7), and Table M2S6 shows some MIR, LINCs, and snoRNAs are being transcribed with gene transcripts in females. Differences in transcriptional start sites (Tables 1 and 2), and transcription of other RNAs with genes (Table M2S6) would explain the large number of isoforms and relatively low number of alternate splicing events identified in females, as well as contributing to differences in the GC content of male and female transcripts (Fig. 5). These differences would also lead to a reduction in the potential for microarrays to identify significantly DE genes because the transcripts differ from known protein coding regions. What is more, microarray normalisation methods that correct for mismatches (MAS5), variance (VSN) and GC content of probes (GC-RMA) remove these differences identifying even fewer significant changes in gene expression. More isoforms are identified in the male versus female comparison (Table 1), and the majority of isoforms are identified in females (Tables 1 and 2). Numerous studies have identified DE of isoforms in males and females^25–30^. A transcript is considered an isoform if it differs from the known protein coding sequences on the reference genome. The reference genome used in this study was generated by the human genome project. The genome was mapped using a bacterial artificial chromosome (BAC) library created from a single male donor (RPCI-11). Ten subsequent BAC libraries were created from a mixture male (n = 1) and female (n = 1) DNA which were pooled and Sanger sequenced. Each sequence library was mapped to the genome using the male (RPCI-11) map^21^. The use of male only DNA to map sequences meant that potential differences in the DNA of males and females could not be identified. In addition the map produced did not contain the second X chromosome from females; the sequences could not be mapped to male DNA, so they were discarded. The methods assumed that the two X chromosomes from females were identical, and that the X chromosome in males was the same as those in females so there was enough X chromosome coverage. Evidence against this assumption is that the Y chromosome in males is clearly not the same as the X chromosome. The human genome project was the benchmark study and all subsequent mammalian genome projects used the same methods. Transcriptomic studies have identified the genes on only one X chromosome are expressed, subsequently theories have arisen that female mammals are mosaics with patches of tissue expressing either the paternally or maternally derived X chromosome^19^. However one considers that females may express the genes on both X chromosomes in all tissues; but we can only measure the expression of genes we know about and can map back to reference genomes. Inability to align transcripts from the second female X chromosome is a current limitation of all mammalian transcriptomic analyses, and all technologies based on reference genomes. Additionally a high degree of variation in X chromosomes observed in experimental data sets could be attributed to the assumption the male X chromosome was the same as both female X chromosomes. Whereas experimentally observed increased variance in the Y chromosome could be due to reduction in the number of samples used, n = 10 versus n = 20 for the rest of the genome. This study shows that females are transcriptionally different, in terms of the genes DE as well as the sequences and sizes of the transcripts. An increase in average transcript size alongside a significant reduction in the total number of transcripts identified using Method Two (Table 1 versus 2) is evidence that separating samples by sex has increased the accuracy and efficiency of *de novo* transcriptome assembly. Sex specific *de novo* transcriptome assembly has further increased statistical power by reducing the number of transcripts against which p-values are corrected during Benjamini Hotchberg multiple testing correction. Clearly, males and females are genetically different in respect of sex chromosomes, however improved transcriptome assembly achieved using Method Two could be considered evidence that males and females are also globally genetically different. A hypothesis that cannot be confirmed or refuted using the genome map created using male DNA; male and female samples should have been sequenced separately and sex specific maps generated. In respect of these observations what evidence do we have that males and females are genetically the same? What technologies/experiments is this based on? And what resolution did they have? Could the accuracy and reliability of transcriptomic and genetic studies be improved by generating male and female reference genomes and completing sex specific genome alignments or *de novo* genome assemblies? In this study age was used to identify DE, but the determination of whether transcriptional start sites differ is dependent on the known protein coding sequences on the reference genome. Differences identified in females may not be a consequence of old age, but rather the sequences and loci of gene transcripts in males and females may differ (Tables 1, 2, and Fig. 5). Method Two was used to determine the impact of separating male and female transcripts in the determination of gene expression. Using sex specific transcript files clarifies the position on TSS, isoforms and splicing by identifying that TSS differ in female tendon and most transcripts are isoforms (Table 2). This also identified that MIRs and LINCs are often being transcribed together as units, and in some cases DE with gene transcripts in females (Table M2S6), leading to reduction in the number counted when filtered from the transcript file (Table 2). Surprisingly the results identify that isoforms are not always a consequence of alternate splicing of exons, sex specific *de novo* transcriptome assembly strongly indicates that miRNAs, lincRNAs contribute to the formation of isoforms. One ponders whether considering small RNAs (miRNA, lincRNA, snoRNA) together with gene transcripts in RNA-seq analyses would improve our understanding of transcription and translation? These results also raise questions about whether genome alignments of RNA-seq data reliably identify isoforms given the assumptions and subsequent limitations of reference genomes, annotated transcript files, short read sequencers and aligners? This study identified transcripts transcribed from the reverse strand, however the direction of transcription cannot be determined from the data or existing technologies. What impact does our assumption of direction have on measured expression levels? And if transcripts are being transcribed from alternate loci backwards from the reverse strand to include small RNAs, does this fit with the triplet code theory of translation?

The utility of transcriptomic results and ease at which they can be interpreted depend on the experimental design, confounding factors, data analysis decisions, assumptions, the accuracy of technologies and tools, as well as statistical methods. The calculation of significance relies on underlying assumptions made about the probability distributions of transcript measurements as well as accurate measurement of mean counts and variance per gene^24^. Separating samples by known biological differences (age and gender) aimed to decrease overall variance and further increase statistical power. Separating samples by sex changed the data distribution from Poisson to Negative Binomial (Fig. 1), an observation that is in line with RNA-seq data distributions observed in other eukaryotes^24,31^. The shift in data distribution reduced skew and increased statistical power leading to the identification of more DE genes. Separating RNA-seq data from males and females put the data into its natural and most powerful distribution (negative binomial), reduced overall variance, potentially improving the accuracy and reproducibility of results. On these basis should males and females be analysed separately in all analyses?

RNA-seq results for old males point to an increase in cell cycle, as well as increases in metabolism, cell stress, and catalytic activity (Figures M1S2, M2S2, M2S3 and M2S4). This could explain an increase in variance. In particular a high cell cycle would give rise to a heterogeneous population of cells in various stages of the cell cycle. A large number of genes are involved in cell cycle processes^32^; therefore RNA extracted from rapidly dividing cells represents a pooled sample of RNA with high variability. Higher variance observed in old male cells (Figure M1S1) may have reduced statistical power leading to a reduction in the number of DE genes identified.

RNA-seq identified increases in p38MAPK signalling (Figure M1S1) increases in metabolism, response to stress, ion binding, catalytic activity and cell proliferation (Figures M2S2–M2S4) in males. NRF2 is activated by p38MAPK in response to reactive oxygen species (ROS) promoting the cell cycle and increasing cell proliferation^33^. Increased levels of the oxytocin receptor (OXTR) have been implicated in activation of the p38MAPK pathway, regulation of energy metabolism, and thermoregulation^34–37^. Ageing leads to increased circulating leptin in males which has been associated with decreased testosterone concentrations and increased BMI. Leptins such as LEP are involved in lipid metabolism^5,38^, and are also regulators of immune and inflammatory responses, nutrient uptake, insulin secretion, and MMP expression (Figure M1S3). Activating natural killer cell receptors (NKC, NKG2C/E, NKG2D, NKG2DL) downregulated in old males in this study are thought to regulate the innate and adaptive immune response through cytokine production and MMP expression leading to the development of inflammatory conditions such as rheumatoid arthritis (RA)^39^. Natural killer cells, Interleukin 1 Receptor Accessory Protein (ILR1RAP) and NFκB were decreased, whilst genes involved in the production of cytokines, the cytokine receptor (IL8, LIF, LEP, SF10D), and JAK-STAT pathway were increased (Table M2S5). JAK-STAT signalling is associated with increased proliferation of cells increased cytokine production and increased expression of cyclinD as evident in this study^33^; which in this study has led to the identification of a variety of cancer pathways (Tables M1S5, M2S4 and M2S5). When Method Two is used to assess mixed tendon tissues as well as TET and Achilles tendon separately, pathway and gene ontology analyses identify age-related decreases in glycosylation, and immune responses alongside increases in fc*γ* R-mediated phagocytosis, RIG-I like receptor signalling, mTOR signalling, PPAR signalling, and pathways in cancer (Tables M2S4 and M2S5, Figures M2S18–M2S26). Glycosylation of immunoglobulins regulates the adaptive immune response and has been implicated in the development of auto-immune diseases including RA^40,41^. All of these processes are up-regulated with an increase in cell cycle, regulating immune signalling and response to stress^42–44^.

**Figure 6:**
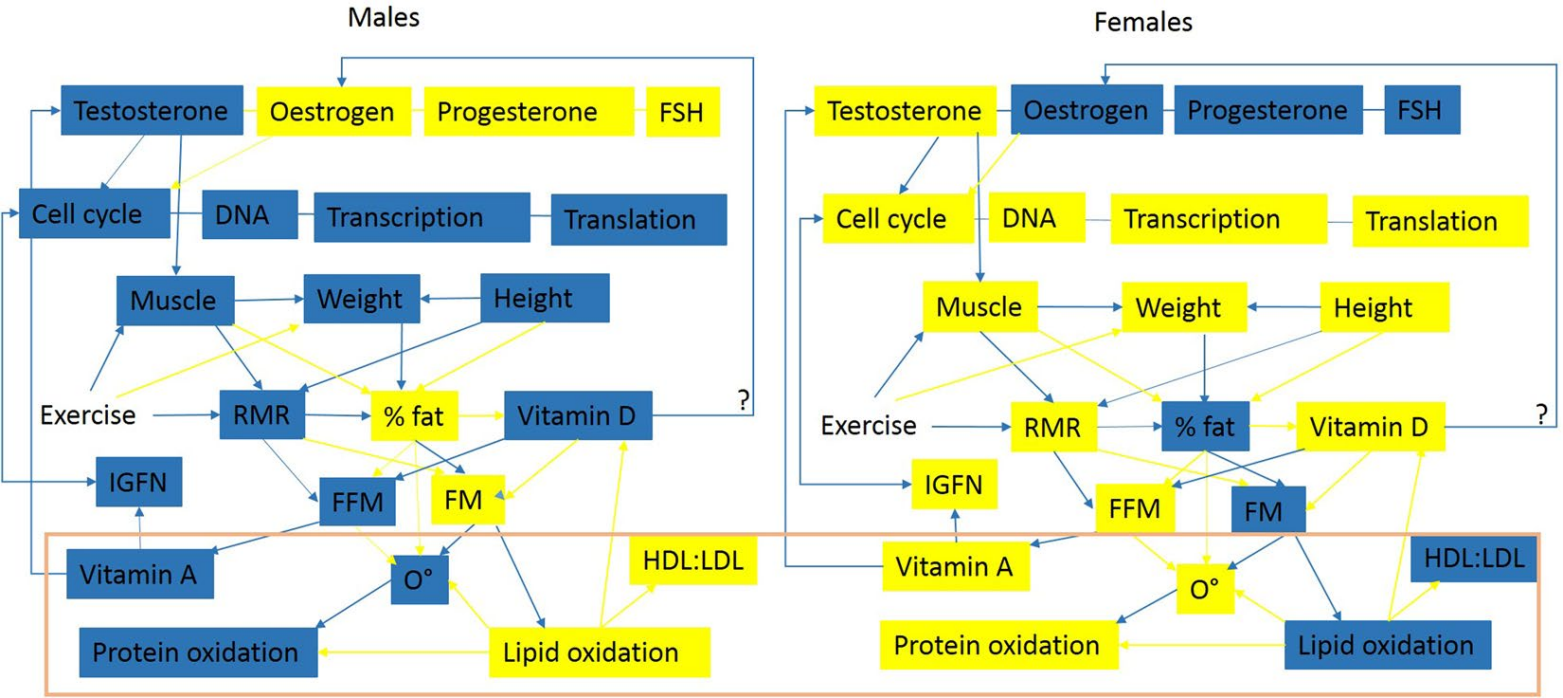
Illustration of some documented differences between males (left) and females (right). Blue boxes depict high entity levels, yellow boxes depict low entity levels, blue arrows show positive regulatory processes, yellow arrows show negative regulatory processes. Entities at the bottom of the diagram surrounded by the orange box are hypothetical, interpreted from relationships identified in existing literature, and inferences that could potentially be made from them. Abbreviations: FFM = fat free mass, FM = fat mass, RMR = resting metabolic rate, FSH = follicle stimulating hormone, O = Reactive Oxygen Species (based on measurement of oxidative stress enzymes). References^29,56,57,59–61^.

Female bias in the development of tendinopathy has been identified in the literature, an effect amplified in old age^1,10,11^ however tendinopathy also disproportionately affects manual workers and athletes^1^. Results for females were in clear contrast to those obtained from males (Figs 2, 3 and 4). Antigen processing and presentation (MHCII) was increased resulting in the identification of a variety of pathways involved in infections and immune responses (Table M1S5). Reductions in the expression of SMAD3, high expression of TGF-*β* as well as reduced p53 signalling, MAPK signalling and sonic hedgehog signalling are indicative of a reduced cell cycle in females (Table M2S4, Table 4)^45,46^. Cell cycle reductions are further supported by reduced DNA replication, RNA transport, base excision repair (Tables M1S5 and M2S4) glucose, fructose, mannose and galactose metabolism (Table M1S5), digestion and absorption of B vitamins, oxidative phosphorylation, and ether lipid metabolism (Table M2S4). Reactome categories for genes significantly decreased included cell cycle processes, glucose metabolism and cholesterol biosynthesis (Table 6). Coupled with increases in sphingolipid metabolism, fatty acid degradation and insulin signalling, these changes suggest potential insulin resistance and degradation of lipids for energy. Aldehyde and alcohol dehydrogenases are increased in expression during vitamin A deficiency, which is consistent with lipid degradation rather than synthesis^47^. The Rac-MEKK-JNK pathway increased in females promotes heat shock (HSP90) gene expression^48^. HSP90 was increased in old females using RNA-seq, and was identified as a significant node in network topology (Figure M2S14). Since HSP90 is required for proper function of steroid hormone receptors^49^, this may be indicative of an androgen deficient state. This is an observation that is supported by the reduction in the steroid biosynthesis pathway in old female TET (Table M1S5). TET were differentiated from MSCs and cultured in the absence of hormones, which could lead to increased expression of hormone responsive pathways. However reductions in hormone concentrations do occur in old age^50^. The availability of hormones is linked to the availability of lipids and both play a role in regulating the cell cycle (detailed in the supplementary discussion; hormones lipids and cell cycle, and the illustration summarising these connections in Fig. 6).

The majority of genes DE in females were decreased in expression, analysis of these genes indicate a decrease in cell cycle. Proliferation of MSCs has been observed to decrease linearly with age^6,51,52^. This has been explained by the DNA clock theory; increased methylation of genes occurs in aged cells reducing their proliferation potential^7–9^. Gender differences in global DNA methylation have been identified. Females have been found to have significantly (p < 0.01) higher methylation than males^53^. Contrary to this Horvath *et al* ^54^. reported that males have significantly higher methylation than females, and that ethnicity also impacts on methylation. Age related changes in DNA methylation could alter transcriptional start sites as observed in this study. Decreased expression of histone de-acetylases in old female TET (Table 8) are evidence of chromatin remodelling that could occur as a consequence of age or be different in TET and tendon tissue. Age-related changes in transcriptional start sites are supported by TET from males where nearly all of the age-related DE transcripts have different transcriptional start sites and more than half are isoforms (Table 2). It is possible that transcriptional start sites, gene and isoform expression differ in TET, as a consequence of differentiation procedures and chemicals used.

RNA-seq data from Achilles tendon (E-MTAB-2449) has previously been analysed^55^. In the study all young tendon donors were male, however old donors were a mix of males (n = 2) and females (n = 3) (Table 12). CRABP2 was identified as decreased in expression four fold by Peffers *et al*.^55^, in this study it was decreased significantly (q < 0.05) as well as two and a half fold in old male mixed tendon tissues. Assessing TET and Achilles tendon separately CRABP2 was not significantly (q < 0.05) DE; however it was decreased four fold and five fold respectively. In old female tendon CRABP2 was also identified as significantly (q < 0.05) and three fold decreased in expression using Method One and four fold using Method Two. Similar results were observed for MMP3. It was increased in old age in the study by Peffers *et al*.^55^, and also increased in old male tendon and in old female tendon in this study. Few other results from this study were in agreement with the study by Peffers *et al*.^55^. Dramatic differences and sometimes a change in direction of expression are seen when samples are separated by gender prior to analysis (Tables 4 and 5). Sex-related differences in the expression of cytochromes, neurexophilin, X chromosome located genes and oestrogen biosynthesis genes were identified in this study. Our results identify the importance of gender as a consideration in -omics studies. Further insight into the accuracy of technologies and interactions between age and gender could be gained by repeating the analysis on healthy donated tendon from young (aged 23–29) and old (aged 63–69) males and females. Based on the distributions and variance observed in this study nine replicates per age and gender group are required to achieve adequate statistical power. Total RNA extracted from each replicate could be split and assessed using microarrays and RNA-seq to determine the accuracy and consistency of the technologies. In terms of analysis male and female RNA-seq samples should be processed separately with *de novo* transcriptome assembly as in this study. More replicates would allow for clustering to separate samples by dominant cell cycle stage and clusters could be analysed separately to further reduce variance. This study highlights some limitations of the reference genome (missing X chromosome, mixture of male and female genomes) the generation of male and female specific reference genomes would facilitate more accurate assessment of gene expression so sequences could be more accurately aligned and transcriptome assemblies improved.

## Summary, conclusions and wider implications

The results of this study show that ageing affects cell cycle, oxidative stress, immune signalling and gene expression differently in males and females. Documented differences between males and females including the presence of different isoforms are supported by cross species studies. Males and females differ in body compositions, fat mass, FFM, genetics, metabolic rates, oxidative stress levels, hormone profiles, longevity and the propensity to develop degenerative conditions. Females have greater longevity which has been correlated with reduced oxidative stress, different oxidative stress bio-markers and hormone profiles^56–59^. It may be the case that females who have higher body fat percentages oxidise lipids to deal with oxidative stress, whereas males who have lower body fat produce proteins to deal with oxidative stress. Oxidative stress in males and females may not differ but rather the methods used to measure them (oxidative stress proteins) have led to this conclusion. However high metabolic and lipid synthesis rates are known to generate high ROS that may underpin higher oxidative stress in males.

A reduction in the steroid bio-synthesis pathway in old females, alongside differences in retinol and lipid metabolism are coupled with a reduction in the cell cycle. Lipids determine whether cells have the ability to proliferate (requirement for cell membrane components, energy) or synthesise hormones, MHCs and vitamin D because lipids are precursors for these molecules. Elevated lipid synthesis is thought to be a hallmark of cancers^62^, however increased lipid synthesis is both a precursor and requirement for cell division.

This study highlights the fundamental importance of gender in large scale studies such as this. Indeed gender may be an overlooked factor that could help account for controversial data, regarding the levels and impacts of genes and proteins in the pathways identified^34,29,63^. In old female TET genes are transcribed from different start sites and many are identified as isoforms, genes from TET could have different transcriptional start sites to tendon, ageing could alter transcriptional start sites, or genes in females could have different transcriptional start sites. Consistency in RNA-seq and microarray studies is limited; differing GC content of transcripts, high numbers of isoforms, alternate splicing, different transcriptional start sites and promoters all act to reduce the sensitivity of microarrays, an effect that is compounded by data processing techniques. This study goes some way to meeting the aims of the three Rs; identifying these compounding factors means in future we can better design studies to avoid confusion and experimental repeats to address anomalies. Taking gender into account is hampered by the lack of reporting in public repositories such as Array Express where gender and age are often not reported. A large number of tendon based gene expression studies were excluded from this study because essential information such as age and gender were not recorded. We would like to see age and gender included in Minimum Information standards such as MIAME which would help overcome current limitations. Refining and grouping samples by biological attributes can improve data distribution calculations greatly increasing statistical power and thus obtain significant results when the number of replicates is low. Our study highlights just some of the factors that need to be considered in the design and analysis of omics studies. Our study identifies a clear requirement for the biology community to conduct well planned gender balanced studies and analyse data generated from males and females separately.

**Table 11:** Details of search queries used in Array Express to identify suitable studies (detailing age and gender of participants) for inclusion in the analysis.

Query string	Studies returned	Tendon specific studies	Suitable study ID
sac:age AND sac:sex AND tendon	14	1	E-MTAB-2449
sac:age AND sac:gender AND tendinopathy	1	1	E-GEOD-26051
sac:age AND sac:sex AND tendinopathy	0	0	None
sac:age AND sac:gender AND tendon	2	1	E-GEOD-26051
tendon	50	3	E-GEOD-26051 E-MTAB-2449
tendinopathy	1	1	E-GEOD-26051
tissue engineered tendon	1	1	None
tissue engineered tendon AND sac:age	0	0	None

## Methods

The research aimed to investigate the effects of ageing on the transcription of genes in tendon tissues. TET samples were differentiated as described in^64^, sense strand RNA libraries were prepared and sequenced as described in^65^ and^66^. Studies for incorporation in the analysis were identified by filtering by organism (*Homo sapiens*), experiment type (RNA assay) and constructing search queries in array express (Table 11). For comparative purposes the number of tendon studies returned without age or gender specifications are recorded. No gene expression studies assessing TET from humans were publicly available, one study E-MTAB-3732 identified using this search term did not contain age or gender phenotypic data. The filtering process identified only two studies using tendon (E-MTAB-2449 and E-GEOD-26051) where sample attribute columns (sac) age and gender were available (Table 11).

Summary data for RNA-seq samples from TET (E-MTAB-4879), tendon (E-MTAB-2449), and their associated attributes are shown in Tables M1S1 and M2S2. E-MTAB-2449 RNA samples from tendon tissues were processed by Peffers using the same methods and equipment as tissue engineered tendon described in^55^. Technologies used and available replicates for each age and gender group for each technology are shown in Table 12.

**Table 12:** Details of the technologies and additional samples available for assessment of gene expression in old and young tendon.

Technology	Young males	Old males	Young females	Old females	Sample type
Illumina HiSeq. 2000	4	2	0	3	Achilles tendon^55^
Age ranges	14–27	60–74	None	66–79	Achilles tendon^55^
Illumina HiSeq. 2000	1	4	3	0	TET
Age ranges	25	54–74	20–22	None	TET
Affymetrix HGU133 plus 2	11	4	3	5	various tendons^22^
Age ranges	36–58	59–66	46–58	59–65	various tendons^22^
Totals	16	10	6	8	tendons n = 40

More young males were assayed in the microarray experiment (Affymetrix) than the RNA-seq experiments (Illumina). Only young TET and old tendon samples were available for females.

### RNA-seq data analysis

**Figure 7:**
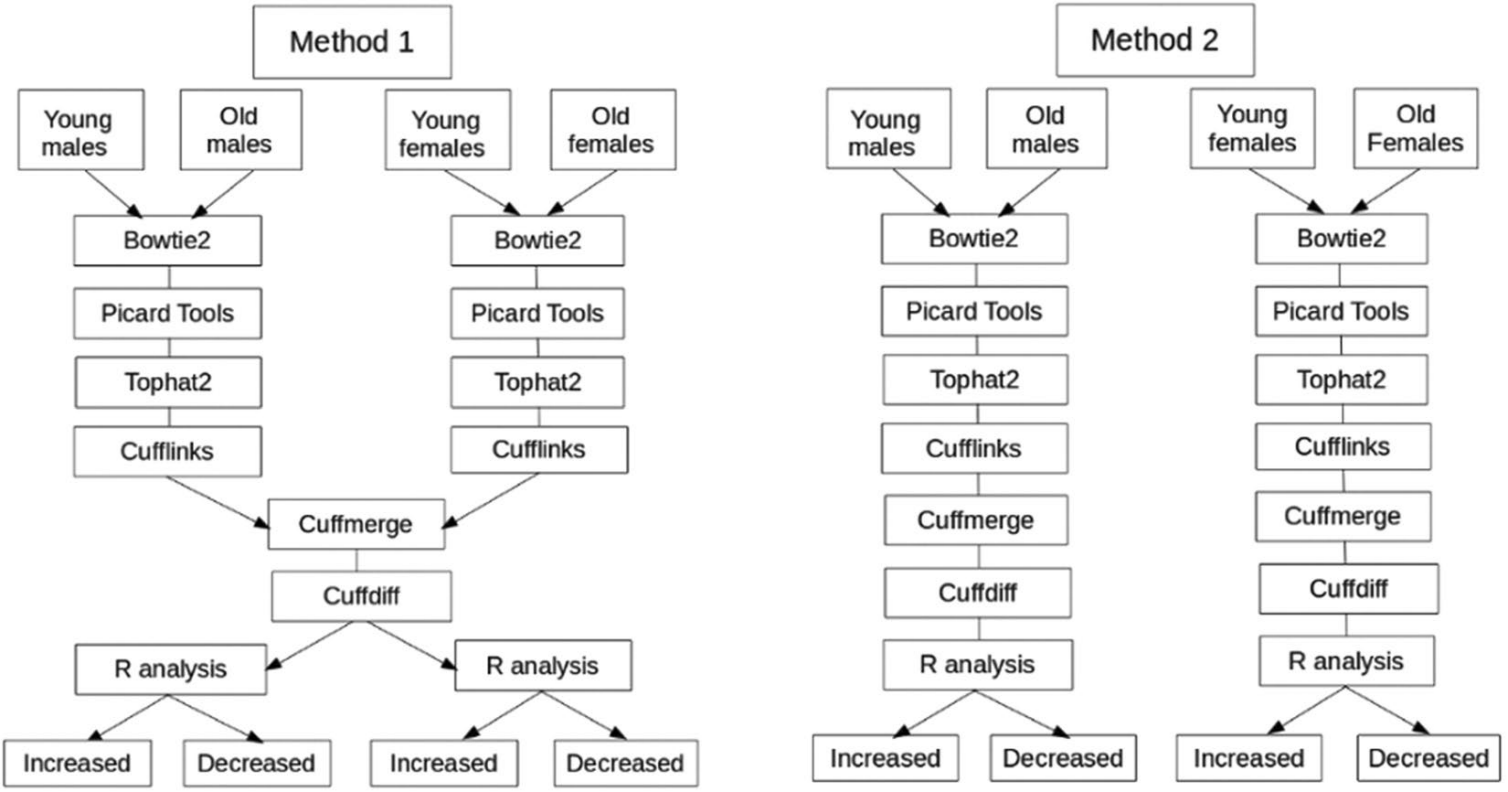
Flow diagram of methods used in the analysis of RNA-seq data. Method One: CuffMerge and CuffDiff were completed on male (young n = 5, old n = 6) and female (young n = 3, old n = 3) subsets to ensure male and female result comparability. Male and female data was separated at each stage of downstream analysis. Method Two: Bowtie2, Tophat2, Cufflinks, CuffMerge, CuffDiff were completed on male (young n = 5, old n = 5, (mixed T/TET was young n = 4, and old n = 5)) and female (young n = 3, old n = 3) subsets separately to ensure the model selected was appropriate for the sex specific transcripts and data distributions. Male and female data was separated at all stages of analysis.

Two methods were applied to RNA-seq data, the first (Method One) assessed age and gender related differences in gene expression in the same analysis using a merged transcript file generated by merging male and female transcripts. The second (Method Two) aimed to identify sex specific differences in transcripts by analysing males and females in separate pipelines with sex specific merged transcript files (Fig. 7).

#### Method One

Paired end fastq files were quality checked using fastqc, aligned to the hg19 reference genome using Bowtie2^67^ with the very sensitive setting unpaired reads were excluded (bowtie2 –very-sensitive –no-mixed -x hg19*). SAM files were converted to sorted BAM files using SAMtools^68^ and insert sizes, mean inner distance, and standard deviation per sample was estimated using Picard tools (BroadInstitute 2014). Fastq files were aligned with the hg19 genome build using -b2-sensitive settings in Tophat^69^ parameterised using descriptive statistics to produce tophat aligned BAM files. Mapped reads were assembled into transcript files per sample using Cufflinks^70^. Male and female transcript files were merged using CuffMerge to create a merged transcripts file. Phenotypic data; age group and gender (young male, young female (<35), old male, old female (>54) was used to assign samples to groups. FPKM normalised data was significance tested using the cross replicate pooled condition dispersion method in CuffDiff. CuffDiff was completed on male and female data sets combined (Fig. 7). A dispersion model based on each replicated condition was built then averaged to provide a single global model for all conditions in the experiment. CuffDiff p-values were adjusted using Benjamini-Hochberg Multiple Testing Correction (BH-MTC)^71^ to generate q-values^72^. The number of transcripts, isoforms, promoters, transcriptional start sites and alternate splicing identified by CuffDiff were recorded (Table 1). Additionally the total number of transcripts tested were determined by counting the number of lines in the gene expression cuffdiff output file. Small RNAs (miRNA, lincRNA, snoRNA, scaRNA) were filtered from the transcripts using grep in command line and the number of lines counted, the mean and range of transcript sizes were determined using retrospective functions in R (Table 1). Quality and data distributions were assessed using cummeRbund^73^. Data quality and distribution was checked using Squared Cumulative Variance (CV^2^) plots and PCA plots (Figure M1S1). Cullen and Frey graphs were generated in R using fitdistrplus^74^ (Fig. 1). To ascertain the direction of change of genes conditional statements were written in R to subset male and female genes with a significant (q < 0.05) and 1.5 fold increase or decrease in expression (Tables M1S2). Methods are summarised in the schematic in Fig. 7. An overview of the results is shown in Table 1. Analysis without gender separation was conducted for young and old tendon for each technology to identify significantly (q < 0.05) differentially expressed genes (Table M1S4). For completeness a male versus female comparison of gene expression without age separation was also completed for microarray and RNA-seq data (Table M1S3).

#### Method Two

The impact of including the younger (54 year old) male, including adapter regions, and assessing differential expression in males and females simultaneously was controlled for by re-analysing male and female data sets in completely separate pipelines (Fig. 7, Method Two). The 54 year old male (T68YR5) was excluded (Tables M2S1), 10 nucleotide adapter regions were trimmed and reads aligned using Bowtie2 as described above. Trimmed fastq files were aligned with the hg19 genome build using a stranded experiment (first stand) and -b2-sensitive settings in Tophat^69^ and parameterised using descriptive statistics to produce tophat aligned BAM files. Mapped reads were assembled into transcript files per sample using Cufflinks with the first strand setting^70^. Male and female specific transcript files were merged using CuffMerge to create sex specific merged transcript files. Separate CuffDiff analyses were conducted for male and female data sets (Method Two, Fig. 7). Phenotypic data; age group and gender (young male, young female (<35), old male, old female (>60) was used to assign samples to groups. RNA-seq analysis (Bowtie2, Tophat2, CuffLinks, CuffMerge, CuffDiff) were completed for a mixture of tendon and TET, for this analysis T24YR8 was removed to reduce variance, since it was the only young male TET sample it could not be determined whether this sample was representative of young male TET. To test the assumption that age affects tendon and TET similarly young male versus old male comparisons were completed for tendon and TET data sets separately. CuffDiff analysis was repeated as described above. The number of transcripts, isoforms, promoters transcriptional start sites and alternate splicing events identified by CuffDiff were recorded (Table 2). The total number of transcripts tested were determined by counting the number of lines in the gene expression cuffdiff output file. Small RNAs (miRNA, lincRNA, snoRNA, scaRNA) were filtered from the transcripts using grep in command line and the number of lines counted, the mean and range of transcript sizes were determined using retrospective functions in R (Table 2). Cullen and Frey graphs were generated in R using fitdistrplus^74^ (Figure M2S2). To ascertain the direction of change of genes conditional statements were written in R to subset male and female genes with a significant (q _<_ 0.05) and 1.5 fold increase or decrease in expression (Table M2S2). A matrix of gene count values for genes significantly (q < 0.05) DE in old female tendon tissues was merged with genes significantly (q < 0.05) DE in old male tendon. Hierarchically clustered heatmaps of genes significantly (q < 0.05) DE in male and female tendon were generated in R using the heatmap function^75^ (Fig. 2). Young and old TET from males and young TET from females were compared (Fig. 3). Old female tendon and young female TET was also compared to mixed tendon tissues from young and old males (Fig. 4). Significantly (q < 0.05) and 1.5 fold DE genes in young male tendon (n = 4) versus old male tendon (n = 2) and young male TET (n = 1) versus old male TET (n = 3) from males were determined (Table M2S3).

#### Sequence properties

Sequences for transcripts DE in males and females (Fig. 5) were retrieved using Biomart^76^ ensembl hg19 libraries from chromosome, transcriptional start sites and transcript end sites. Potential differences in sequence properties were probed by determining the percentage GC content of transcripts from males and females using the GC function of the seqinr package^77^. The percentage differences in GC content was determined by subtracting female GC content from male GC content (Fig. 5). DE transcripts with different GC contents included some microRNAs and long non-coding RNAs. RNAs other than mRNA that were identified as significantly (q < 0.05) DE were subset from the count data using grep (Table M2S6).

#### Network analysis

Significantly DE gene lists for males and females identified using Method Two were uploaded to Network Analyst to assess significant interactions and modules^78^. The zero order network was visualised and functionally analysed by identifying enriched KEGG, Gene Ontology, reactome and motif categories for females. The top ten most significant reactome categories for females are shown in Tables 6 and 7. In males a smaller list of significant genes meant the first order network was analysed. Network topology diagrams generated by Network Analyst for up and down regulated genes identified in old females (Figure M2S14), old mixed tendon tissues versus young mixed tendon tissues (Figure M2S15). Additionally up and down regulated genes in old tendon versus young tendon in males (Figure M2S16) and old TET versus young TET in males (Figure M2S17) were assessed. Chord diagrams generated from lists of up and down regulated gene symbols were used to visualise genes changed in the same directions in tendon and TET, and mixed tendon analyses (Figure M2S13). DE transcripts with different GC contents were uploaded to Network Analyst and significant KEGG pathways and reactome enrichment identified (Tables M2S7 and M2S8).

### Microarray data analysis

#### Normalisation methods: Impact on results

To assess the impact of different normalisation methods the Array Express package in Bioconductor was used to import raw data. Data was normalised using Variance Stabilising Normalisation (VSN), Robust Multi-array Analysis (RMA), Variance Stabilising Normalisation with Robust Multi-array Analysis (VSN-RMA), Guanine-Cytosine affinity correction with Robust Multi-array Analysis (GC-RMA), and MicroArray Suite 5 (MAS5) (Table 3). RMA normalisation was chosen because; it identified significantly DE genes in males and females (Table 3), it did not correct for differences in GC content (Fig. 5) differences in transcriptional start sites and sequences (potential mismatches) (Tables 1 and 2), or differences in variance (Figure M1S1).

#### Selected microarray analysis methods

The Array Express package in Bioconductor was used to import processed data (RMA normalized) from E-GEOD-26051, the data was log_2_ transformed. Limma^79^ was used to assign samples to groups based on phenotypic data (age groups; young = under 59 (only ages available), old = 60 and over), gender, and disease state. Phenotypic data was used to exclude diseased tendon: Only samples from healthy tendon in young and old participants were compared. Contrasts were created in Limma, fit to a linear model and standard error smoothed using empirical Bayes for males and females to identify genes that were significantly (q < 0.05) and 1.5 fold increased and decreased in expression in old age (Table M1S2). To test the impact of sample balancing male age groups were re-defined as young (35–50 n = 3) and old (<59, n = 3) and the data re-analysed (Table M1S1, male array balanced).

### Comparative analysis

For both Methods One and Two gene symbols for each of the conditions were written out from the R console and intersecting genes for possible comparisons identified using grep –Fx in command line (Tables M1S3 and M2S3).

### Gene Ontology and pathway analysis

Gene lists were written out from the R console for each tissue type, condition, technology (microarray and RNA-seq) and method (Fig. 7). Unranked gene symbol lists with a background universal list of gene symbols for all known transcripts (RNA-seq) or gene symbols for all array probes (microarrays) were submit to GOrilla^80^. Significantly (q < 0.05) affected Gene Ontology categories were reduced for visualisation using REVIGO^81^. Scatterplots and TreeMaps were selected to visualise GO categories (Figures M1S2–M1S10 and Figures M2S2–M2S12 for mixed tendon tissue analyses). This process was repeated for genes significantly (q < 0,05) and 1.5 fold differentially expressed in tendon and TET from old males (Figures M2S18–M2S26). Gene identifiers for DE genes and background universal lists were converted to Entrez IDs and KEGG pathway analysis was completed on each gene list; results were visualized using KEGGgraph^82^ and pathview^83^. KEGG p-values were corrected using hypergeometric testing and significant (q < 0.05) categories are summarised in Tables M1S5 and M2S5 for Methods One and Two respectively.

## Electronic supplementary material

Supplementary File
