# Supplementary material for: Cross platform analysis of transcriptomic data identifies ageing has distinct and opposite effects on tendon in males and females
Source: Sci Rep. 2017 Oct 31;7:14443. doi: 10.1038/s41598-017-14650-z (PMC5663855; doi:10.1038/s41598-017-14650-z)
Supplement: Supplementary file 1 — Supplementary File [file 41598_2017_14650_MOESM1_ESM.pdf]

# Cross platform analysis of transcriptomic data identifies ageing has distinct and opposite effects on tendon in males and females

**Louise. I. Pease[1], Peter. D. Clegg[1,2,] Carole. J. Proctor[1,3,] Daryl. J. Shanley[1,4,] Simon. J. Cockell[5], and Mandy. J. Peffers[*1,2]**

The sample identifier allows the sample origins to be traced for instance T24XR3 identifies a Tissue engineered tendon (T) sample from young (24), female (X) donor replicate three (R3). For RNAseq three replicates are available however no old females were sampled and only one young male was assayed; gender affects global gene expression; male and female samples are not comparable.

Table M1S1 Details of tissue engineered tendon sample donors (left) and Tendon donors (right) for biological replicates of RNAseq data

| Cell | Age | Gender | Replicate | sample identifier | Processing and data deposits |
|---|---|---|---|---|---|
| Tissue engineered tendon | 20 | X | 3 | T24XR3 | Mandy Peffers Liverpool University 2016 |
| | 22 | X | 5 | T24XR5 | E-MTAB-4879 |
| | 20 | X | 6 | T24XR6 | |
| | 25 | Y | 8 | T24YR8 | |
| Tissue engineered tendon | 67 | Y | 3 | T68YR3 | Mandy Peffers Liverpool University 2016 |
| | 74 | Y | 4 | T68YR4 | E-MTAB-4879 |
| | 54 | Y | 5 | T68YR5 | |
| | 67 | Y | 6 | T68YR6 | |
| Tendon | 21 | Y | 1 | TP24YR1 | Mandy Peffers Liverpool University |
| | 16 | Y | 2 | TP24YR2 | E-MTAB-2449 (April 2014) |
| | 27 | Y | 3 | TP24YR3 | |
| | 14 | Y | 4 | TP24YR4 | |
| Tendon | 66 | X | 1 | TP68XR1 | Mandy Peffers Liverpool University |
| | 68 | X | 2 | TP68XR2 | E-MTAB-2449 (April 2014) |
| | 74 | Y | 3 | TP68YR3 | |
| | 60 | Y | 4 | TP68YR4 | |
| | 79 | X | 5 | TP68XR5 | |

Table M1S2 The number of significantly (q<0.05) differentially expressed genes RNAseq and microarray in age for each gender with 1.5 fold change in expression.

| Regulation | | Up | Down |
|---|---|---|---|
| Male | RNAseq | 82 | 77 |
| | Array | 16 | 0 |
| | Array (balanced) | 0 | 1 |
| Female | RNAseq | 297 | 452 |
| | Array | 618 | 618 |

Table M1S3 The number of significant (p<0.05) genes 1.5 fold up and down regulated identified in combinations of gender, age and technology comparisons

| Comparison | Technology | Up | Down |
|---|---|---|---|
| Both genders | RNA-seq | 0 | 1 |
| | Array | 0 | 0 |
| | Both Technologies | 0 | 0 |
| Males | Both Technologies | 0 | 0 |
| Females | Both Technologies | 0 | 0 |
| old male tendon V old male tissue engineered tendon | RNA-seq | 5 | 1 |
| young male tendon V young male tissue engineered tendon | RNA-seq | 1 | 0 |
| Male V Female | Array | 0 | 0 |
| Male V Female | RNA-seq | 24 | 43 |
| Young female vs Young male | RNA-seq | 297 | 71 |
| Old female vs Old male | RNA-seq | 278 | 9 |

Table M1S4 The number of significant (q<0.05) genes with 1.5 fold up and down regulated genes measured using microarrays and RNAseq using BH-MTC without gender separation.

| | Both genders | |
|---|---|---|
| Regulation | Up | Down |
| Array significant (q<0.05) | 0 | 0 |
| RNAseq significant (q<0.05) | 51 | 62 |

Table M1S5 Pathways significantly (q<0.05) represented by significantly (q<0.05) differentially expressed genes that were 1.5 fold up and down regulated measured using microarrays and RNAseq, upregulated genes and pathways, downregulated genes and pathways, genes with high (<3) fold changes are noted with an asterisk (*).

| | Pathways microarrays | Gene Symbols / enzyme names | Pathways RNAseq | Gene Symbols / enzyme names |
|---|---|---|---|---|
| Male genes increased in expression | Nitrogen metabolism | 4.2.1.1 (carbonate dehydratase), 6.3.4.16 (carbamoyl-phosphate synthase), 1.4.1.3 (glutamate dehydrogenase), 6.3.1.2 (glutamine synthetase) | Calcium signalling<br>cytokine -cytokine receptor pathway<br>Neuroactive ligand receptor pathway<br>Focal adhesion caveolin<br>JAK-stat signalling pathway<br>Bacterial invasion of epithelial cells<br>CHAGAS disease<br>GLIOMA<br>Melanoma<br>Bladder cancer<br>Chronic myeloid leukemia | GPCR, PHK<br>IL8, LIF, LEP, SF10D<br>HTR, BDKRB, OXTR, LEP<br>Caveolin, Shc, CycD<br>Cytokines, Hormones, CycD<br>Shc, Caveolin<br>B2R, IRAK, IL8<br>Shc, cyclinD<br>GF, cyclinD<br>Cyclin D1, IL-8<br>Shc, CyclinD1 |
| Male genes decreased in expression | ----- | ---- | Antigen processing and presentation<br>Natural killer cell mediated cytotoxicity<br>Leishmaniasis<br>Malaria<br>Toxoplasmosis<br>Amoebiasis<br>Colorectal cancer<br>Renal cell carcinoma<br>Pancreatic cancer<br>Chronic myeloid leukemia<br>Asthma<br>Rheumatoid arthritis | MHCII, KIR<br>NKG2C/E, NKG2D, NKG2DL<br>TGFβ, MHCII<br>NKC, TGFβ<br>MHCII, TGFβ<br>TGFβ<br>TGFβ<br>TGFBeata<br>TGFβ<br>TGFβ<br>MHCII, FceR1<br>MHCII, TGFBeata |
| Females genes | Sphingolipid metabolism | DEGS, | Glycolysis / gluconogenesis | Aldehyde dehydrogenase, alcohol dehydrogenase |

| | | | | |
|---|---|---|---|---|
| increased in expression | Protein export<br>Non-homologous end joining<br>Ubiquitin mediated proteolysis<br><br>Dorso ventral axis formation<br>Neurotrophin signalling<br><br><br><br>Long term depression<br><br>Insulin signalling<br><br>GnRH signalling<br><br><br>Colorectal cancer | 3-dehydrosphinganine reductase,<br>Ceramidase,<br>ceramide synthetase,<br>acylsphingosine deacylase,<br>sphingomyelin synthase,<br>Ceramide glucosyltransferase,<br>Galactosylceramidase,<br>Sphingolipid Delta(4)-Desaturase 1,<br>SRP19, SRPRB, SEC11, SRP72, SEC61β<br>Mre11, XRCC4, Artmeis<br>E6AP, UBE3B, Itch, HERC4, UBE4B, Skp1,<br>HIP2, Mdm2, cIAPs, PIAS, PML, BTB,<br>Cul3, UBE2N<br>Sos, Notch, Ras85D, Rolled, Yan, Orb*<br>SOS, Ras, Shc, TrkC, cAMK, PSEN*, SC-1,<br>ASK1, JNK, MEK1/2, Erk1/2, FKHRL1,<br>GSK3β<br>Ras, MEK1/2, ERK1/2, PP2A, G, PLC,<br>IGF1<br>INSR, JNK, SHC, SOS, Ras, MEK1/2,<br>ERK1/2, GSK-3β, AMPK, GK*, PYG,<br>PKA, PGC-1α, aPKC<br>PLCβ, Sos, Ras, MEK1/2, ERK1/2, JNK,<br>MEKK*, CaMK, IP3R<br>Axin, GSK-3β, K-Ras, TGFβ, TGFβR1,<br>hMSH2, ERK, hMSH6, c-Fos | Fatty acid degradation<br><br>Tyrosine metabolism<br><br>Retinol metabolism<br>Xenbiotics<br>Drug metabolism<br>ABC transporters<br>Phagosome<br>Vascular smooth muscle contraction<br>Coagulation cascade<br>Antigen processing and presentation<br>MHCI pathway<br>MHCII pathway<br>Leishmaniasis<br>Toxoplasmosis<br>Staphylococcus aureus<br>Asthma<br>Systemic lupus erythematosis<br>Hypertrophic cardiomyopathy<br>Dialated cardiomyopathy<br>Viral myocarditis | Aldehyde dehydrogenase, alcohol dehydrogenase, long chain acyl-CoA dehydrogenase<br>Tyrosine monooxygenase, monoamine oxidase, alcohol dehydrogenase (oxidoreductases)<br>ADH, DHRS<br>Alcohol dehydrogenase<br>Alcohol dehydrogenase, monoamine oxidase<br>ABCA5-10<br>Dynein, MHCII, TLR4, CD36, TSP, iC3b, cathepsin<br>CRLR, AC, PKC, IRAG, s-GC, MLCP, MHC<br>VWF, TM, PAR3,4, FH, C3, Clusterin, C6,7,8,9<br>HSP70, Ii, MHCII, SLIP, CTSB/LS, CLIP,<br>TLR2/4, C3b, C3bi, MHCII<br>TLR4, Laminin, tgHSP70, MKK3/6, MHCII,<br>PI3K<br>C3, HF, MHCII<br>MHCII, MB<br>H2A, H2B, MHCII, C7, MAC, C3<br>ITGA, Desmin, Laminin, DHPR, Titin<br>ITGA, Desmin, Titin, DHPR, AC, Laminin<br>MHCII, laminin |
| Females genes decreased in expression | RNA transport<br><br><br>DNA replication<br><br><br>Base Excision repair | RNAseqP, Nup43, Nup54, Gemin8, eIF4E,<br>FMRP, EIF2B, ACIN1, THOC5<br><br>RFC1, α1 e3, RNaseH2B,<br>SSB,<br><br>MBD4, XRCC1, Polε | Glycolysis / gluconeogenesis<br><br><br><br><br>Fructose and manose metabolism<br>Galactose metabolism<br>Steroid biosynthesis<br><br>Amino sugar and nucleotide sugar metabolism<br>Butiriosin and neomycin biosynthesis<br>Neuroactive ligand-resecptor interaction<br>P53 signalling pathway<br><br>Lysosome | Glucose phosphomutase, phosphoglyceraldehyde dehydrogenase, aldehyder dehydrogenase, phosphoglyceralkinase, glycerate phosphomutase, pyruvate kinase, lactate dehydrogenase, thiose phosphoisomerase<br>Phosphofructokinase, fructose aldolase<br>hexokinase<br>Squalene synthase, squalene monooxygenase, methylsterol monooxygenase, 7-dehydrocholesterol reductase, DWF5<br>Hexosaminidase, hexokinase, phosphoglucomutase<br>Hexokinase<br>GR1<br>p14ARF, IGF-BP3, PAI, Noxa, PAG608, TSAP6, Cdc2<br>AP3, cathepsins, TPP1, HEXA/B, LIMP, NPC, LALP70<br>TfR, vATPase, Rab7, TUBA, TUBB, CALR, SRA1,<br>SRB1, αVβ5, TSP, α5β1 |

| | | | | |
|---|---|---|---|---|
| | | | Phagosome<br>Focal adhesion<br><br><br>ECM receptor interaction<br>RENIN Angiotensim<br>Neurotrophin signalling pathway<br><br>Protein digestion and absorption<br>Parkinsons<br>Amoebiasis<br>Pathways in cancer<br><br>Glioma<br>Bladder cancer<br>Small cell lung cancer | ECM, ITGA, ITGB, Actinin, Fuilamin, PI3K, GF, Shc, MLC, CycD, RhoGAP<br>Collagen, Laminin, THBS, Integrin α3, Integrin α5, Tenascin, Integrinβ5,Agrin, CD44<br>MME, CTSA, AP-N<br>Shc, PI3K, CaMK, RhoGD1, NRAGE, NADE, IRAK, 14-3-3E<br>Peptidase, collagen<br><br>Hsp27, COL, Laminin, EhRab, Actinin, PI3K<br>Wnt, Frizzlebox, ECM, ITGA, HSP, Fu, Glut1, VEGF, HIF-α, p14/ARF, p21, p16/INK4, CyclinD1, p15INK4b<br>Shc, PI3K, p21, p14ARF, CyclinD1, p16INK4<br>MMPs, VEGF, p21, p16INK4, CyclinD1<br>ECM, ITGA, PI3K, COX-2, CyclinD1, p15INK4b |

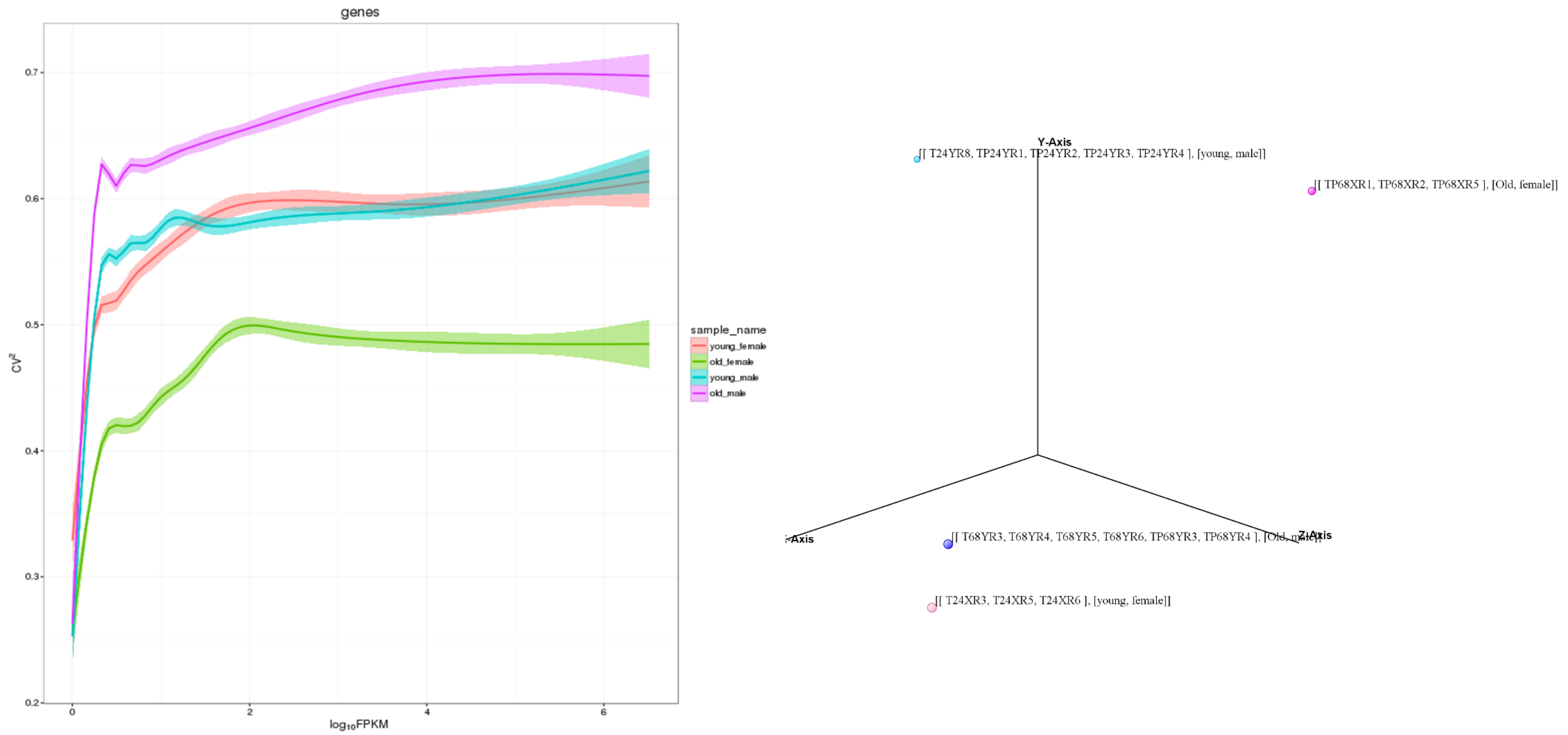


Figure M1S1 Squared cumulative variance plot (left) of RNAseq samples from old and young tissue engineered tendon and tendon from males and females shows young males and females have similar variances, as cells age variance in gene expression reduces in female cells, and increases in male cells. PCA plot (right) of RNAseq samples from tissue engineered tendon (T) and tendon (TP) from young (24) and old (68) males (blue) and females (pink) shows that young males cluster with old females and old males cluster with young females.

Significant Gene Ontology categories represented by genes increased in expression in old males

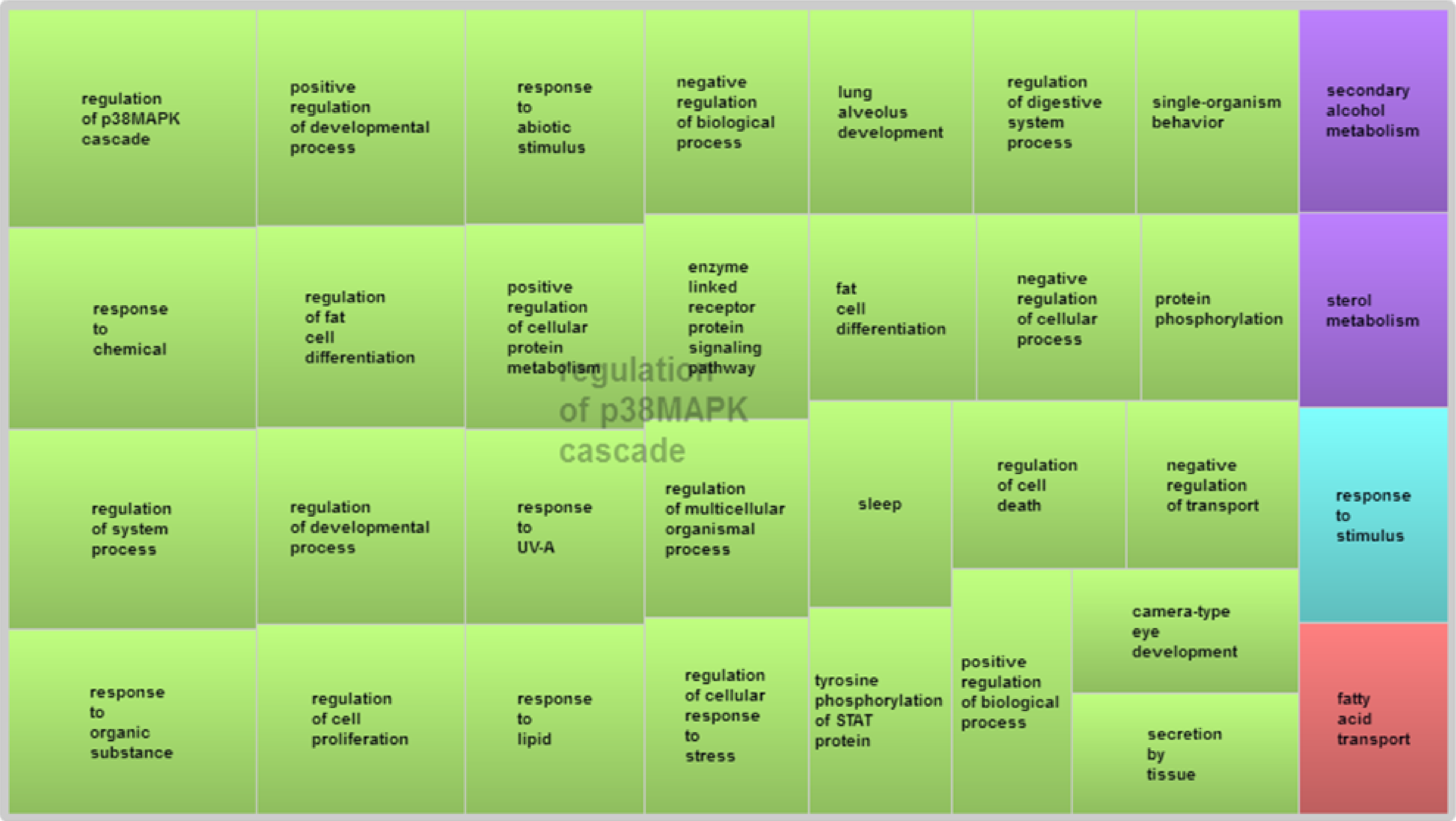


Figure M1S2 REVIGO output TreeMap of Gene Ontology Biological Process terms over-represented by genes significantly increased in expression in old males (n=70) identified using RNASeq. Maps are coloured by category and sized by log10 p-value

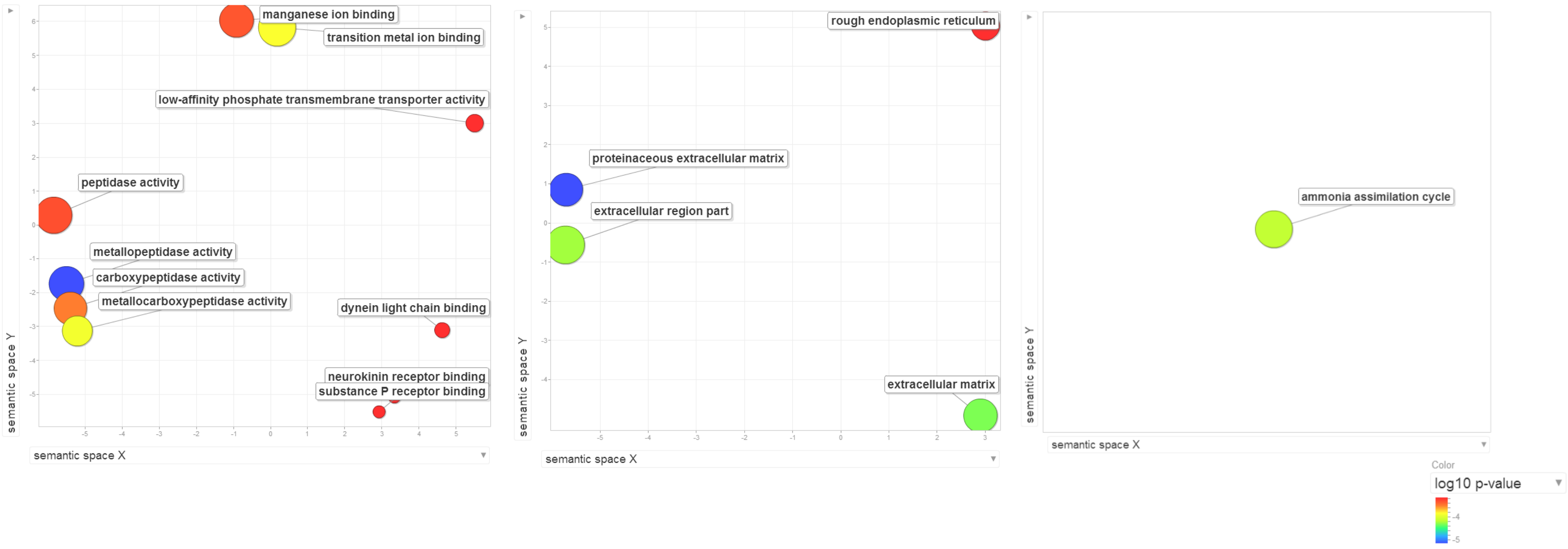


Figure M1S3 REVIGO output scatter plot of Gene Ontology Biological Process (left), Molecular Function (middle), Cellular Component (right) terms over-represented by genes significantly increased in expression in old male cells identified using microarrays

Significant Gene Ontology categories represented by genes increased in expression in old females

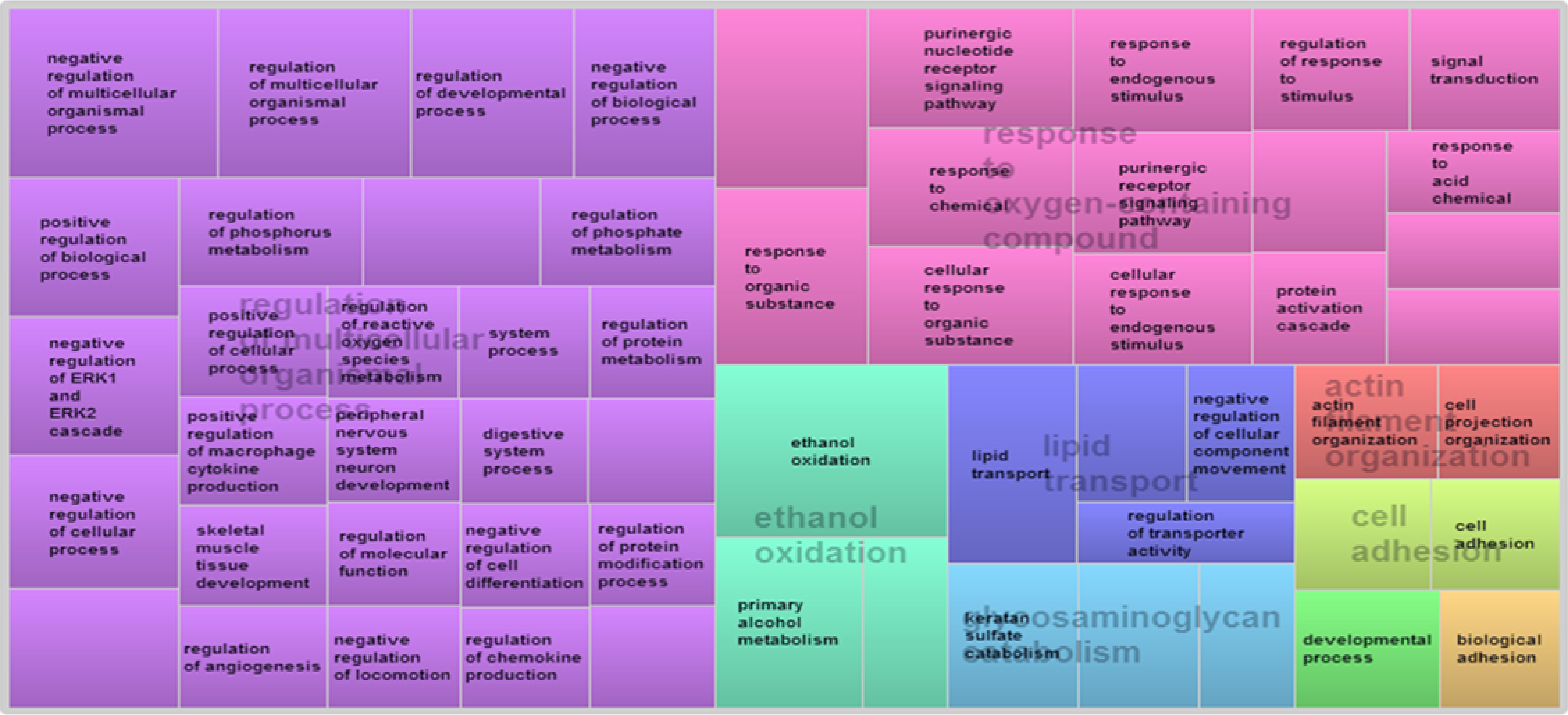


Figure M1S4 REVIGO output TreeMap of Gene Ontology Biological Process terms over-represented by genes significantly increased in expression in old females (n=123) identified using RNASeq. Coloured by category and sized by log10 p-value

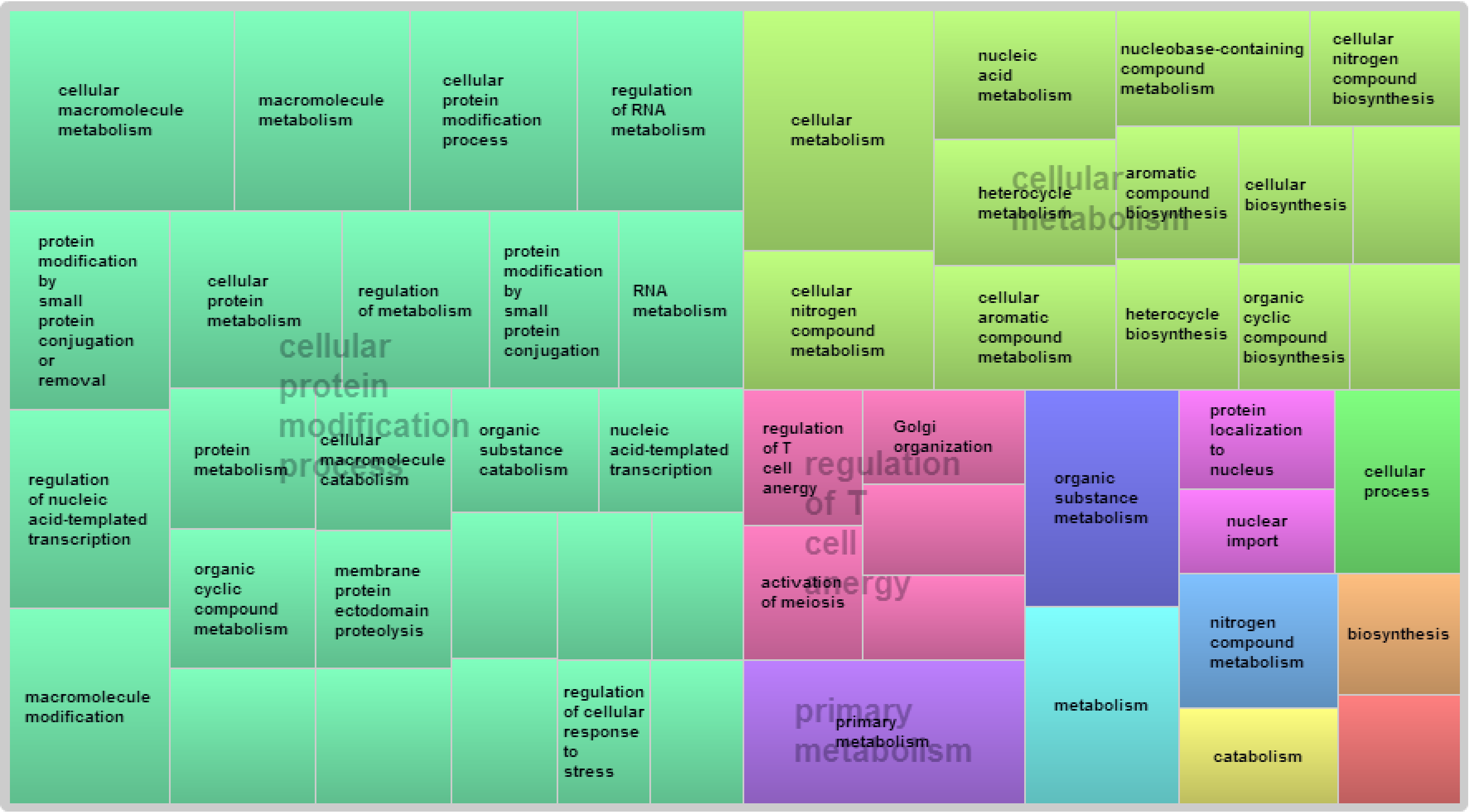


Figure M1S5 REVIGO output tree map of Gene Ontology Biological Process terms (n=84) over-represented by genes significantly increased in expression in old female cells identified using microarrays. Coloured by category and sized by log10 p-value

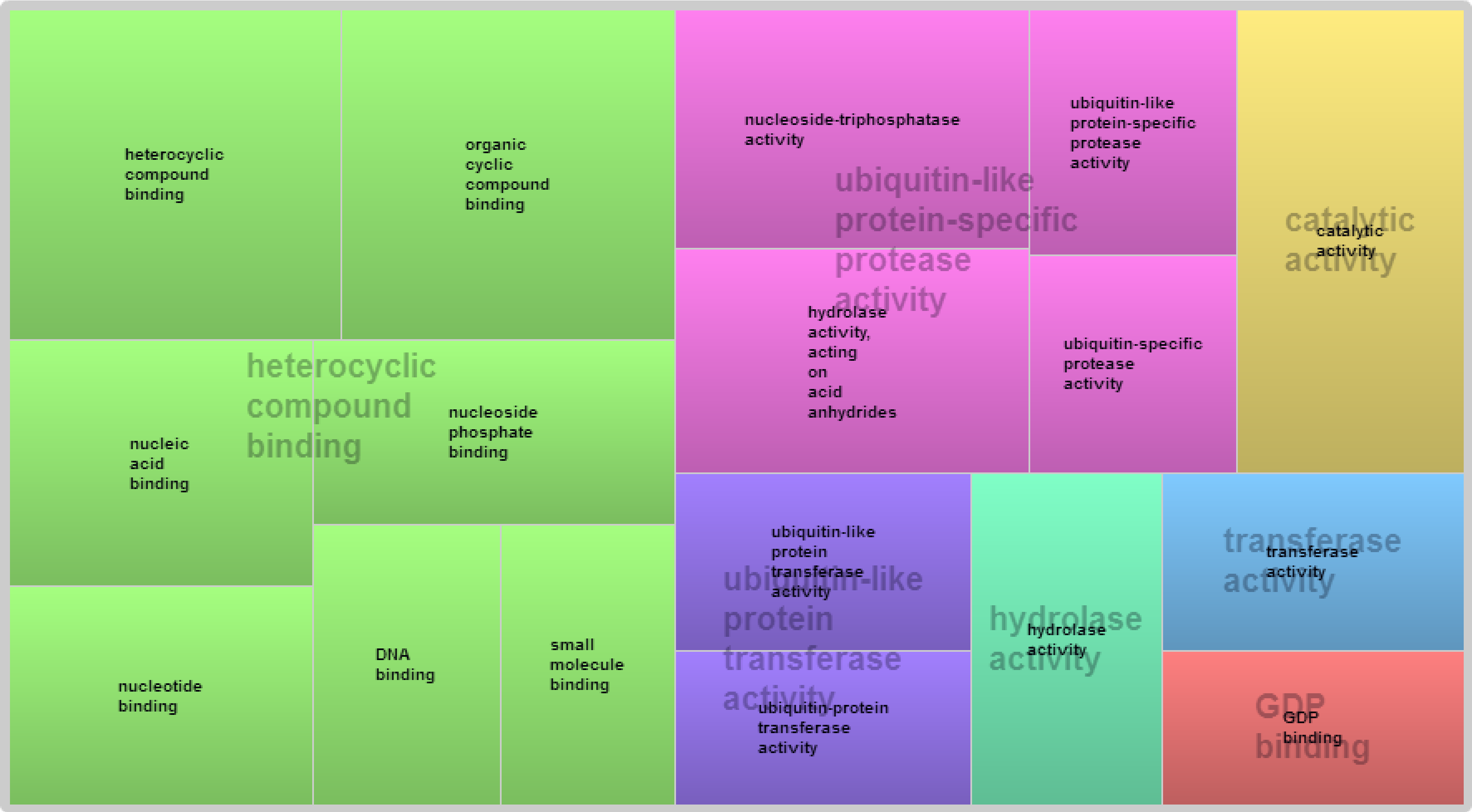


Figure M1S6 REVIGO output tree map of Gene Ontology Molecular Function terms (n=27) over-represented by genes significantly increased in expression in old female cells identified using microarrays Coloured by category and sized by log10 p-value

Significant Gene Ontology represented by genes decreased in expression in old females

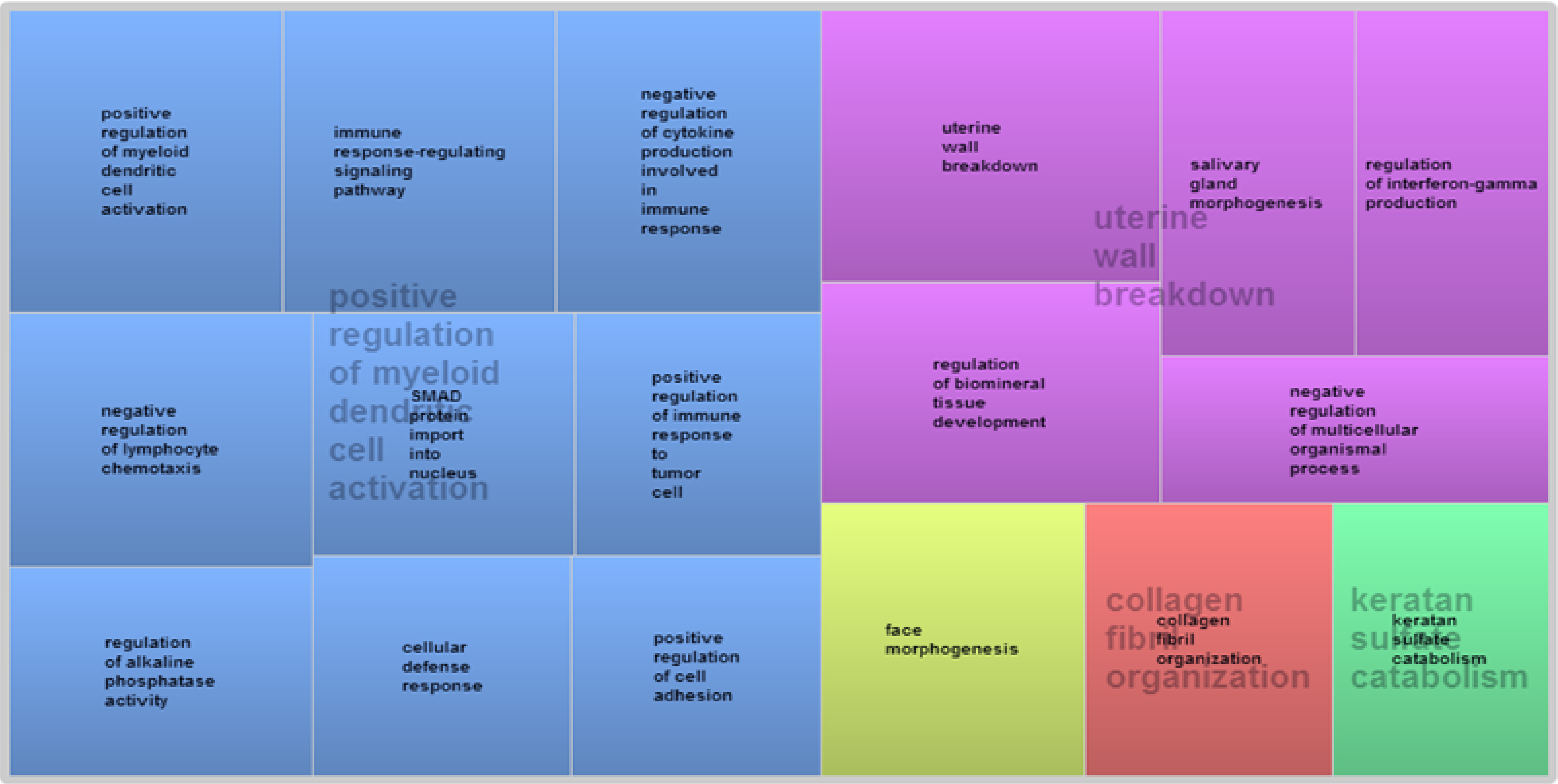


Figure M1S7 REVIGO output TreeMap of Gene Ontology Biological Process terms over-represented by genes significantly decreased in expression in old males (n=48) cells identified using RNASeq. Coloured by category and sized by log10 p-value

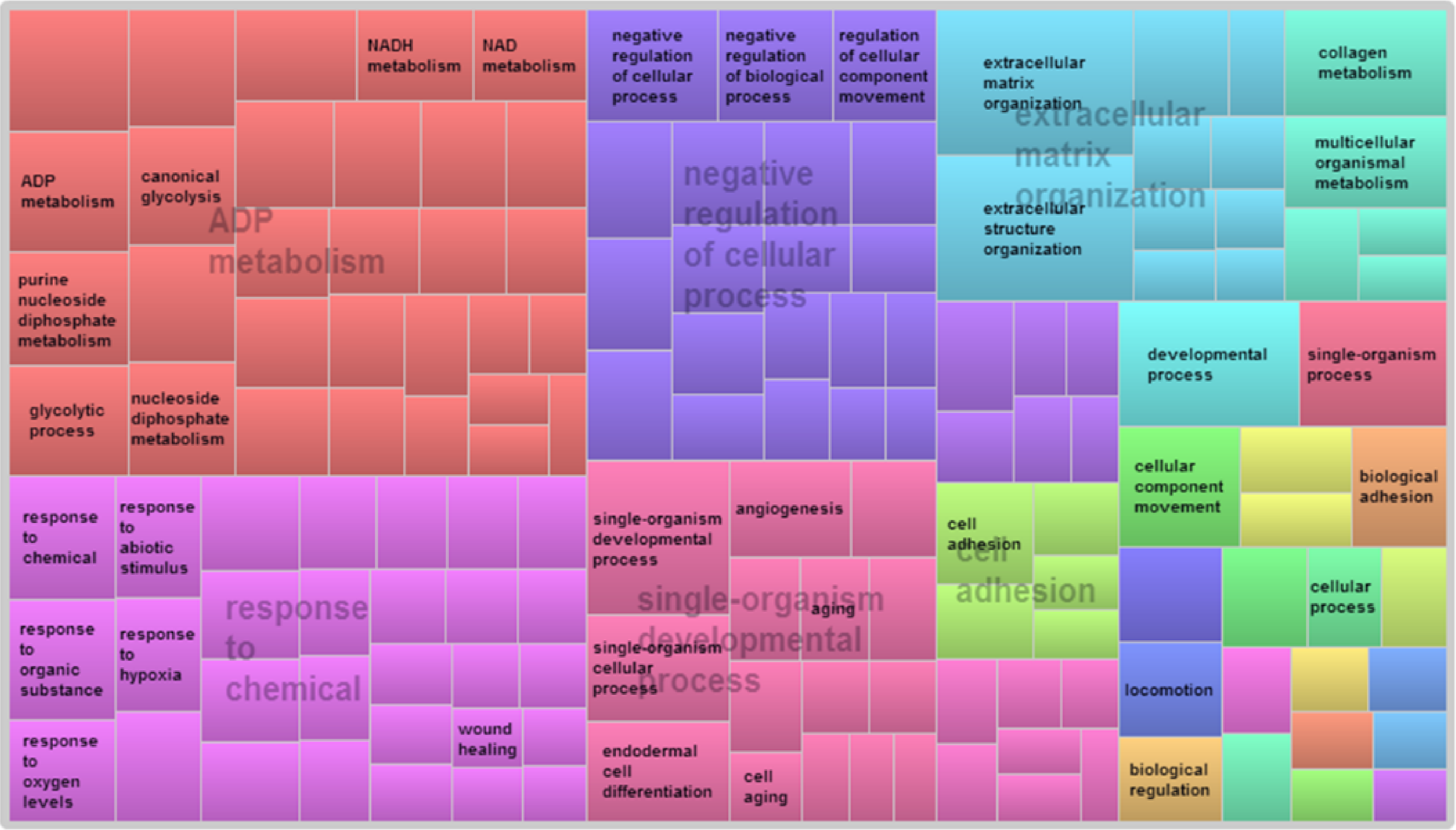


Figure M1S8 REVIGO output TreeMap of Gene Ontology Biological Process terms over-represented by genes significantly decreased in expression in old females (n=334) cells identified using RNASeq. Coloured by category and sized by log10 p-value

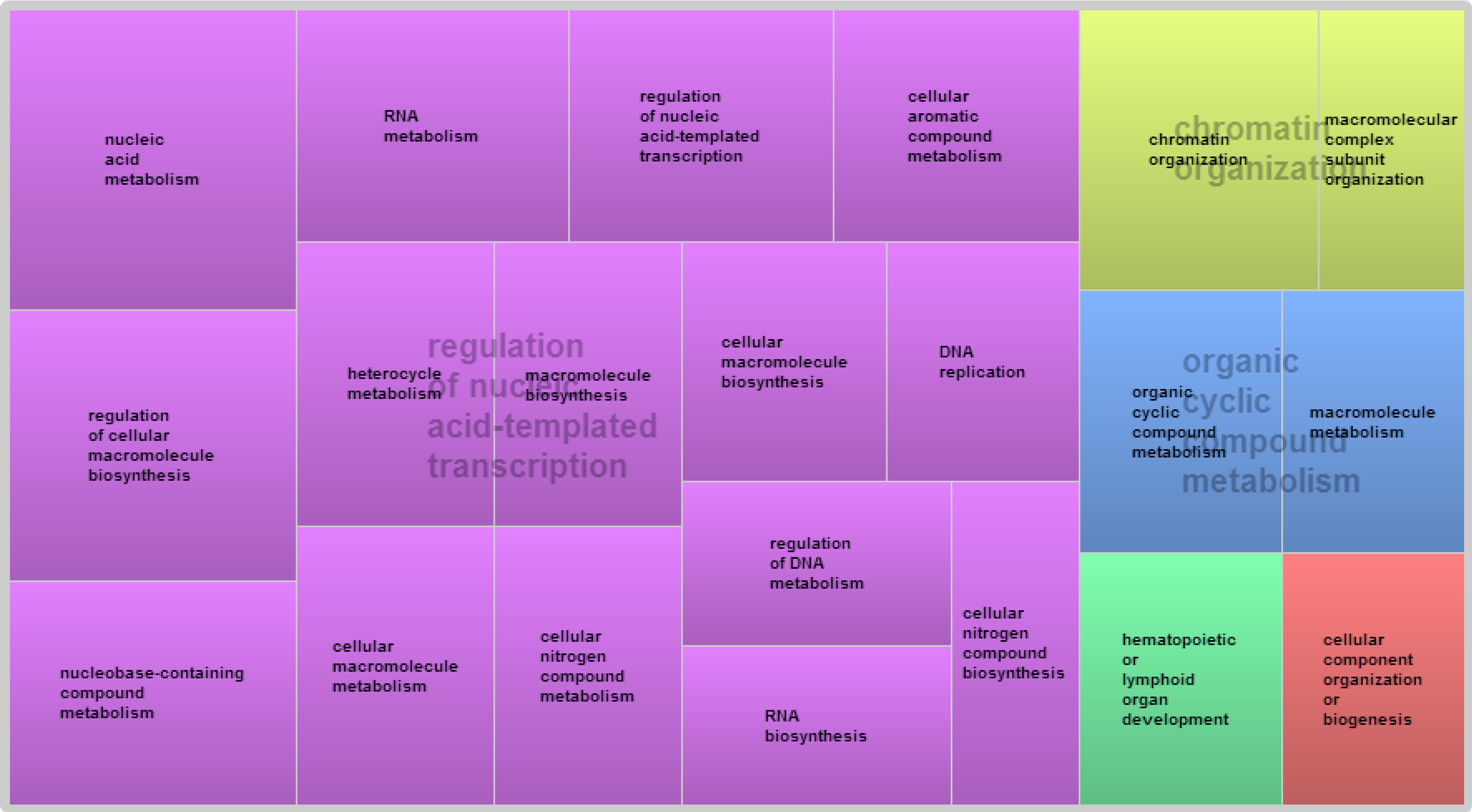


Figure M1S9 REVIGO output tree map of Gene Ontology Biological Process terms (n=37) over-represented by genes significantly decreased in expression in old female cells identified using microarrays Coloured by category and sized by log10 p-value

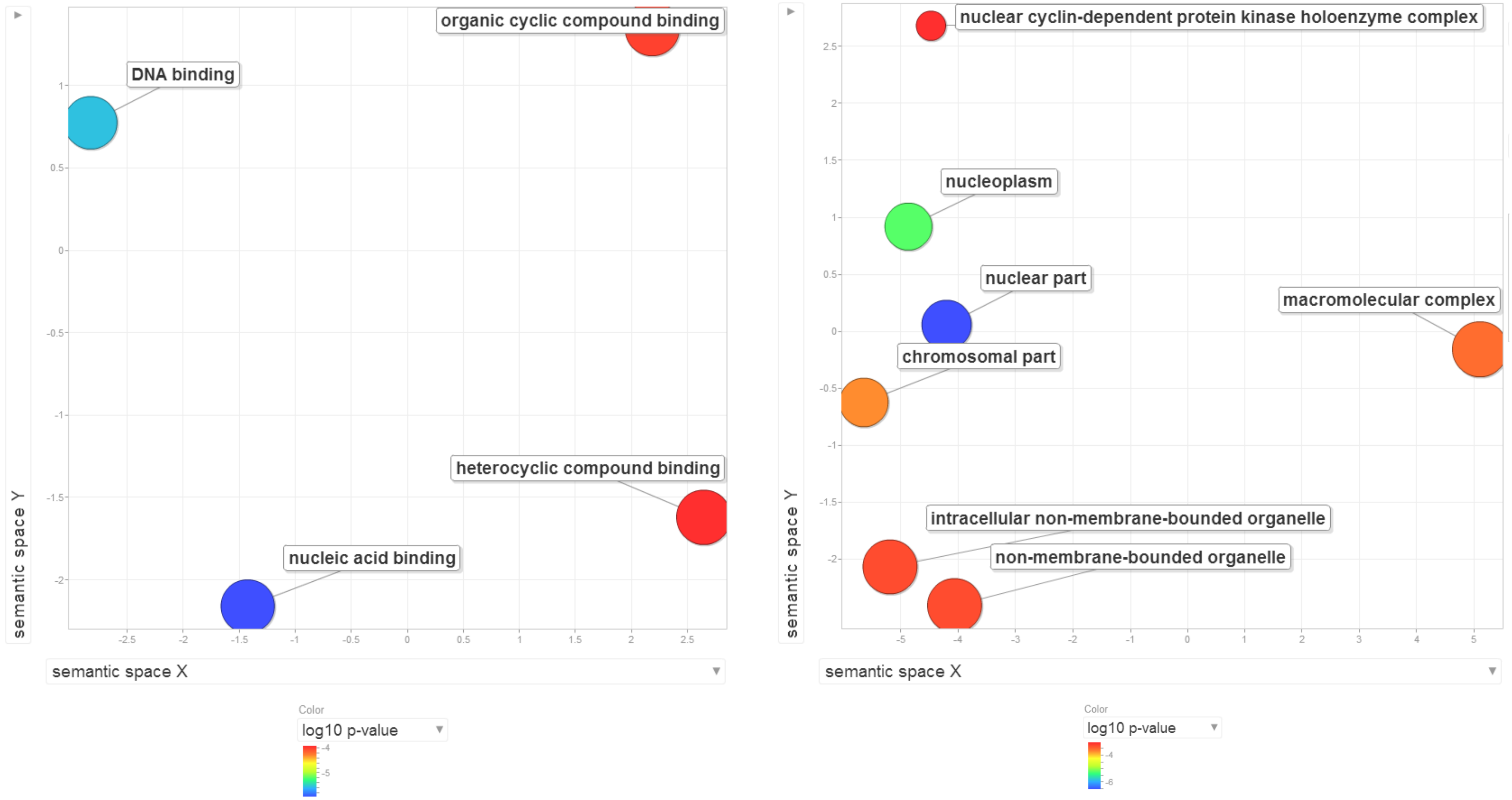


Figure M1S10 REVIGO output scatter plot of Gene Ontology Molecular Function (n=4, left), Cellular Component (n=7, right) terms over-represented by genes significantly decreased in expression in old female cells identified using microarrays

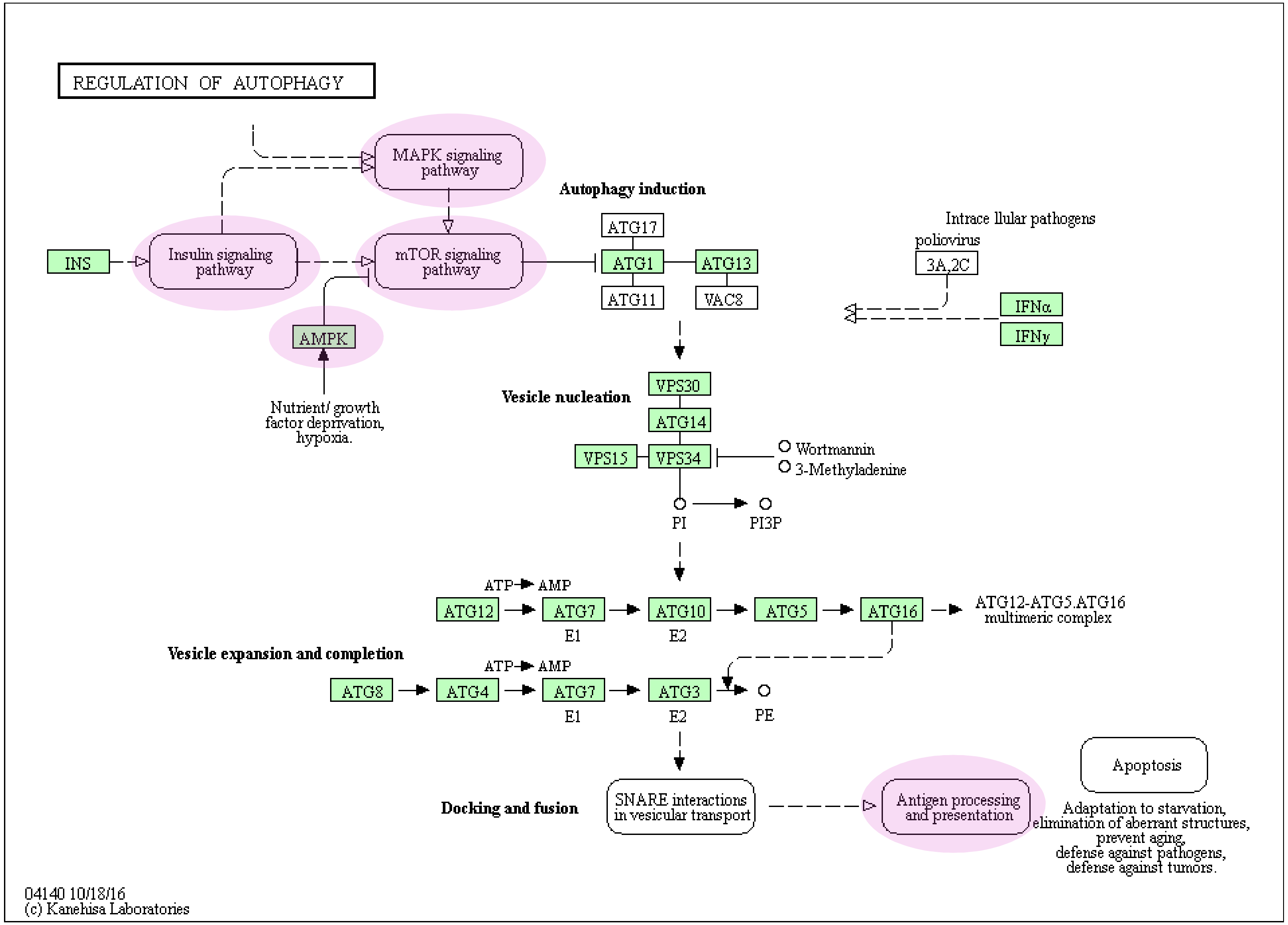


Figure M1S11. Kegg pathway map of regulation of autophagy, genes and pathways identified as affected by ageing in females are circled (Image courtesy of KEGG: Kanehisa, M. and Goto, S. KEGG: Kyoto Encyclopedia of Genes and Genomes. *Nucleic Acids Research* 2000;28(1):27-30. Kanehisa, M., *et al.* KEGG as a reference resource for gene and protein annotation. *Nucleic Acids Research* 2016;44(D1):D457-D462.)

# Cross platform analysis of transcriptomic data identifies ageing has distinct and opposite effects on tendon in males and females

**Louise. I. Pease[1], Peter. D. Clegg[1,2,] Carole. J. Proctor[1,3,] Daryl. J. Shanley[1,4,] Simon. J. Cockell[5], and Mandy. J. Peffers[*1,2]**

The sample identifier allows the sample origins to be traced for instance T24XR3 identifies a Tissue engineered tendon (T) sample from young (24), female (X) donor replicate three (R3). For RNAseq three replicates are available however no old females were sampled and only one young male was assayed; gender affects global gene expression; male and female samples are not comparable.

Table M2S1 Details of tissue engineered tendon sample donors and Tendon donors for biological replicates of RNAseq data

| Cell | Age | Gender | Replicate | sample identifier | Processing and data deposits |
|---|---|---|---|---|---|
| Tissue engineered tendon young | 20 | X | 3 | T24XR3 | Mandy Peffers Liverpool University 2016 |
| | 22 | X | 5 | T24XR5 | E-MTAB-4879 |
| | 20 | X | 6 | T24XR6 | |
| | 25 | Y | 8 | T24YR8 | |
| Tissue engineered tendon old | 67 | Y | 3 | T68YR3 | Mandy Peffers Liverpool University 2016 |
| | 74 | Y | 4 | T68YR4 | E-MTAB-4879 |
| | 67 | Y | 6 | T68YR6 | |
| Tendon young | 21 | Y | 1 | TP24YR1 | Mandy Peffers Liverpool University |
| | 16 | Y | 2 | TP24YR2 | E-MTAB-2449 (April 2014) |
| | 27 | Y | 3 | TP24YR3 | |
| | 14 | Y | 4 | TP24YR4 | |
| Tendon old | 66 | X | 1 | TP68XR1 | Mandy Peffers Liverpool University |
| | 68 | X | 2 | TP68XR2 | E-MTAB-2449 (April 2014) |
| | 74 | Y | 3 | TP68YR3 | |
| | 60 | Y | 4 | TP68YR4 | |
| | 79 | X | 5 | TP68XR5 | |

Table M2S2 The number of significantly (q<0.05) differentially expressed genes identified in old age using Method 2 for each gender with 1.5 fold change in expression.

| Regulation | | Up | Down |
|---|---|---|---|
| Male | RNAseq (tendon) | 43 | 90 |
| | RNAseq (TET) | 100 | 56 |
| | RNAseq (mixed) | 862 | 11 |
| Female | RNAseq (TET vs tendon) | 1637 | 4907 |

Table M2S3 The number of significant (p<0.05) genes 1.5 fold up and down regulated identified using Method 2 in combinations of gender, age and technology comparisons

| Comparison | Technology | Up | Down |
|---|---|---|---|
| Both genders | RNAseq | 0 | 1 |
| | Array | 0 | 0 |
| | Both Technologies | 0 | 0 |
| Males (Tendon Method 2) | Both Technologies | 0 | 0 |
| Males (tissue engineered tendon Method 2) | Both technologies | 0 | 0 |
| Males (mixed Method 2) | Both technologies | 0 | 0 |
| Females (Method 2) | Both Technologies | 0 | 0 |

Table M2S4 Pathways significantly (q<0.05) represented by significantly (q<0.05) differentially expressed genes that were 1.5 fold up and down regulated measured using Method 1 and Method 2 for the analysis of RNAseq data, upregulated genes and pathways, downregulated genes and pathways, genes with high (<3) fold changes are noted with an asterisk (*).

| | Pathways M1 RNAseq | Gene Symbols / enzyme names | Pathways M2 RNAseq | Gene Symbols / enzyme names |
|---|---|---|---|---|
| Male genes increased in expression | Calcium signalling<br>cytokine -cytokine receptor pathway<br>Neuroactive ligand receptor pathway<br>Focal adhesion caveolin<br>JAK-stat signalling pathway<br>Bacterial invasion of epithelial cells<br>CHAGAS disease<br>GLIOMA<br>Melanoma<br>Bladder cancer<br>Chronic myeloid leukemia | GPCR, PHK<br>IL8, LIF, LEP, SF10D<br>HTR, BDKRB, OXTR, LEP<br>Caveolin, Shc, CycD<br>Cytokines, Hormones, CycD<br>Shc, Caveolin<br>B2R, IRAK, IL8<br>Shc, cyclinD<br>GF, cyclinD<br>Cyclin D1, IL-8<br>Shc, CyclinD1 | ABC transporters<br>Ubiquitin mediated protelysis<br><br>Cardiac Muscle contraction<br>Pathways in cancer<br><br>PI3k-AKT signalling<br><br>Focal adhesion<br><br>Endocytosis<br>proteoglycans in cancer | ABCA3, ABCB7, ABCC5<br>PIRH2, Apc2, NEDD4, WWP1, ARF-BR1, SOCSbox<br>DHPR, Cyto<br>CyclinD, GLI, COX-2, VEGF, TRAFs, Cyclin D1, PKB, ECM, EGFR, HGF, FGF, MST1, JNK, Bax, MMPs, PMLRAR alpha, Dvl<br>TSC1, GF, RTK, ECM, ITGA, AKT, p21, CCND1, Cyclin, GYS, GbetaL, Bim<br>ECM, ITGA, PIP5K, AktPKB, Cyclin D, JNK, Rap1, P130Cas, GF, RTK<br>cPML, RTK, EPS15, clathrin, AP-2, ARH, GRK, PIP5K, EHD1, ACP33, His, ArfGAP<br>IP3R, Ankynn, Cyclin D, FN, HGF, FN, alpha5beta1, EGFR, VEGFA, MMP2, uPAR, AKT |
| Male genes decreased in expression | Antigen processing and presentation<br>Natural killer cell mediated cytotoxicity<br>Leishmaniasis<br>Malaria<br>Toxoplasmosis<br>Amoebiasis<br>Colorectal cancer<br>Renal cell carcinoma<br>Pancreatic cancer<br>Chronic myeloid leukemia<br>Asthma<br>Rheumatoid arthritis | MHCII, KIR<br>NKG2C/E, NKG2D, NKG2DL<br>TGFβ, MHCII<br>NKC, TGFβ<br>MHCII, TGFβ<br>TGFβ<br>TGFβ<br>TGFBeta<br>TGFβ<br>TGFβ<br>MHCII, FceR1<br>MHCII, TGFBeta | -- | -- |
| Females genes increased in expression | Glycolysis / gluconogenesis<br>Fatty acid degradation<br><br>Tyrosine metabolism<br><br>Retinol metabolism<br>Xenbiotics<br>Drug metabolism<br>ABC transporters<br>Phagosome<br>Vascular smooth muscle contraction<br>Coagulation cascade<br>Antigen processing and presentation | Aldehyde dehydrogenase, alcohol dehydrogenase<br>Aldehyde dehydrogenase, alcohol dehydrogenase, long chain acyl-CoA dehydrogenase<br>Tyrosine monooxygenase, monoamine oxidase, alcohol dehydrogenase (oxidoreductases)<br>ADH, DHRS<br>Alcohol dehydrogenase<br>Alcohol dehydrogenase, monoamine oxidase<br>ABCA5-10<br>Dynein, MHCII, TLR4, CD36, TSP, iC3b, cathepsin<br>CRLR, AC, PKC, IRAG, s-GC, MLCP, MHC<br>VWF, TM, PAR3,4, FH, C3, Clusterin, C6,7,8,9<br>HSP70, Ii, MHCII, SLIP, CTSB/LS, CLIP, | RNA transport<br><br>Tyrosine metabolism<br><br>Vitamin digestion and adsorption<br>ABC transporters<br>non-homologous end joining<br>Photo transduction | Nup58/45, Nup54, Nup214, pom121, eIF2, PABP, THOC6<br>Alcohol dehydrogenase<br><br>IF-R<br>ABCA3, ABCB2, ABCC10, ABCG5<br>RAd50, XLF<br>RK, PDE, GCAP |

| | | | | |
|---|---|---|---|---|
| | MHCI pathway<br>MHCII pathway<br>Leishmaniasis<br>Toxoplasmosis<br>Staphylococcus aureus<br>Asthma<br>Systemic lupus erythematosis<br>Hypertrophic cardiomyopathy<br>Dialated cardiomyopathy<br>Viral myocarditis | TLR2/4, C3b, C3bi, MHCII<br>TLR4, Laminin, tgHSP70, MKK3/6, MHCII, PI3K<br>C3, HF, MHCII<br>MHCII, MB<br>H2A, H2B, MHCII, C7, MAC, C3<br>ITGA, Desmin, Laminin, DHPR, Titin<br>ITGA, Desmin, Titin, DHPR, AC, Laminin<br>MHCII, laminin | | |
| Females genes decreased in expression | Glycolysis / gluconeogenesis<br><br><br><br><br>Fructose and manose metabolism<br>Galactose metabolism<br>Steroid biosynthesis<br>Amino sugar and nucleotide sugar metabolism<br>Butiriosin and neomycin biosynthesis<br>Neuroactive ligand-resecptor interaction<br><br>P53 signalling pathway<br><br>Lysosome<br>Phagosome<br>Focal adhesion<br><br>ECM receptor interaction<br><br>RENIN Angiotensim<br>Neurotrophin signalling pathway<br>Protein digestion and absorption<br><br>Parkinsons<br>Amoebiasis<br>Pathways in cancer<br><br>Glioma<br>Bladder cancer<br>Small cell lung cancer | Glucose phosphomutase, phosphoglyceraldehyde dehydrogenase, aldehyder dehydrogenase, phosphoglyceralkinase, glycerate phosphomutase, pyruvate kinase, lactate dehydrogenase, thiose phosphoisomerase<br>Phosphofructokinase, fructose aldolase<br>hexokinase<br>Squalene synthase, squalene monooxygenase, methylsterol monooxygenase, 7-dehydrocholesterol reductase, DWF5<br>Hexosaminidase, hexokinase, phosphoglucomutase<br>Hexokinase<br>GR1<br>p14ARF, IGF-BP3, PAI, Noxa, PAG608, TSAP6, Cdc2<br>AP3, cathepsins, TPP1, HEXA/B, LIMP, NPC, LALP70<br>TfR, vATPase, Rab7, TUBA, TUBB, CALR, SRA1, SRB1, αVβ5, TSP, α5β1<br>ECM, ITGA, ITGB, Actinin, Fuilamin, PI3K, GF, Shc, MLC, CycD, RhoGAP<br>Collagen, Laminin, THBS, Integrin α3, Integrin α5, Tenascin, Integrinβ5,Agrin, CD44<br>MME, CTSA, AP-N<br>Shc, PI3K, CaMK, RhoGD1, NRAGE, NADE, IRAK, 14-3-3E<br>Peptidase, collagen<br><br>Hsp27, COL, Laminin, EhRab, Actinin, PI3K<br>Wnt, Frizzlebox, ECM, ITGA, HSP, Fu, Glut1, VEGF, HIF-α, p14/ARF, p21, p16/INK4, CyclinD1, p15INK4b<br>Shc, PI3K, p21, p14ARF, CyclinD1, p16INK4<br>MMPs, VEGF, p21, p16INK4, CyclinD1<br>ECM, ITGA, PI3K, COX-2, CyclinD1, p15INK4b | Oxidative phosphorylation<br><br><br>Ether lipid metabolism<br><br>Cytokine-cytokine receptor interaction<br><br>Apoptosis<br><br>Hedgehog signalling pathway<br>Toll-like receptor signalling<br>Aminoacyl-tRNA biosynthesis<br>Endocytosis<br><br><br><br>Proteasome<br><br><br>MAPK signalling pathway<br>Focal adhesion<br><br><br>JAK-stat signalling<br>Natural killer cell mediated cytotoxicity<br>Pathways in cancer<br><br><br><br>p53 signallling pathway<br><br>Vitamin digestion and adsroption | COX7A, QCR7, OSCP, V-tupe ATPase (d.F.G), cytochrome c reductase, cytochrome c oxidase, ATP phosphohydrolase,<br>Phospholipase D<br><br>CXCL6, CXCR7, CCL4, CCL3, OSMR, CCL23, CCL8, CCL11, CCL18, SF21, TGFBR1, IL17B, IL17RC, SF19L<br>Calpain, Lamin, PARP, CASP9, Actin, alpha_tubulin, FAP1, A1<br>Kif7<br>TLR6, MIP-1alpha, MIP-1beta,<br>glutamate tRNA ligase, alanine tRNA ligase<br>TGF-BetaR, SARA, endophilin, Epsin, EPS15, Arp2/3, ARH, Dab2, Hsc70, CAPZA, FAM21, ArfGAP, ArfGEF, STAM, VPS26, CHMP6, VPS4, FIP3, Src, PAR3, WIPF, dynamin<br>Rpn5, Rpn7, Rpn11, PA28alpha, Rpn13, Rpt6, Rpt2, Rpt4, Beta5t, alpha1, alph3, alpha6, alpha7, beta1, beta2<br>FGF, CACN, RasGRF, RasGRP, Rap1, SRF, HSP72, MEF2C, TGF-BetaR, Cdb42/Rac<br>ECM, ITGB, Src, FAK, RhoGEF, MLCK, Rac, Rap1<br><br>STAM<br><br>CD94, NKG2CE, CD94, 3BP2, Rac, Rae-1, ULBP1-3<br>GPCR, RhoGEF, FAK, ECM, FGF, RasGEF, DAPK, Rac/Rho, RalGDS, Cdb42/Rac, RXR, PLZFRARalpha, cyclinE, HPH, Rbx1, HDAC, TGFbetaR1, CASP9, Fu<br>Cyclin D, MDM2, MDM1, PIGs, PAG608, Cyclin E, KAI |

| | | | | |
|---|---|---|---|---|
| | | | | THTR, SMVT |

Table M2S5 Pathways significantly (q<0.05) represented by significantly (q<0.05) differentially expressed genes measured using Method 2 for the analysis of male tendon and tissue engineered RNAseq data, upregulated genes and pathways, downregulated genes and pathways.

| | Pathways Males tendon RNAseq | Gene Symbols / enzyme names | Pathways Males tissue engineered tendon RNAseq | Gene Symbols / enzyme names |
|---|---|---|---|---|
| Male genes increased in expression | Pentose and gluconerate interconversions<br>Proteasome<br>Vasopressin regulated water re-absorption<br>Prostate cancer<br>Epstein Barr virus<br>Shigellosis<br>Hepatitis C<br>NOD-like receptor signalling<br>Neurotrophin signalling pathway<br>Herpes simplex infection<br>Leishmaniasis<br>MAPK signalling pathway<br>Toxoplasmosis<br>Toll-like receptor signalling pathway<br>Protein processing in the endoplasmic reticulum<br>Fc gamma R-mediated phagocytosis<br>GnRH signalling pathway<br>Chronic myeloid leukemia<br>RIG-I-like receptor signalling pathway | D-xylose 1-dehydrogenase (NADP(+)).<br>Rpt2<br>AQP4<br>MAPK1, SOS2, ATF4, CREB3L3, CREB3L1, CREB3, CDK2, SOS1, MTOR, MAPK3, E2F1, TP53, NFKBIA<br>TRAF6, CDK2, PTMA, TRAF2, GTF2E1, MAPK8, MAPK14, TP53, NFKBIA, JUN, TAB2<br>MAPK1, WASL, VCL, MAPK3, WAS, MAPK8, MAPK14, NFKBIA<br>MAPK1, TRAF6, SOS2, IKBKE, TRAF2, SOS1, MAPK3, MAPK8, MAPK14, TP53, NFKBIA<br>MAPK1, TRAF6, TAB3, MAPK3, MAPK8, MAPK14, NFKBIA, TAB2<br>SOS1, MAPK3, MAPK8, MAPK14, TP53, NFKBIA, JUN, YWHAE<br>TRAF6, CDK2, IKBKE, TRAF2, MAPK8, NFKBIA, JUN, TAB2, PPP1CA, SRSF5<br>MAPK1, TRAF6, MAPK3, MAPK14, NFKBIA, JUN, TAB2<br>MAPK1, TRAF6, SOS2, DUSP4, ATF4, DDIT3, TRAF2, SOS1, MAPK3, HSPA8, HSPA1A, MAPK8, MAPK14, TP53, JUN, TAB2<br>MAPK1, TRAF6, MAPK3, HSPA8, HSPA1A, MAPK8, MAPK14, NFKBIA, TAB2<br>MAPK1, TRAF6, IKBKE, MAPK3, MAPK8, MAPK14, NFKBIA, JUN, TAB2<br>ATF4, DDIT3, PARK2, PDIA4, TRAF2, UBQLN4, HSPA8, HSPA1A, MAPK8, VIMP<br>MAPK1, ARF6, DNM2, WASL, MAPK3, WAS, ASAP1, DNM1<br>MAPK1, SOS2, ATF4, SOS1, MAPK3, MAPK8, MAPK14, JUN<br>MAPK1, SOS2, SOS1, MAPK3, E2F1, TP53, NFKBIA<br>TRAF6, IKBKE, TRAF2, MAPK8, MAPK14, NFKBIA | Glycosphingolipid biosynthesis<br>RIG-I like receptor signalling<br>Fcgamma R- mediated phagocytosis<br>Regulation of actin cytoskeleton<br>Pathways in cancer<br>Bile secretion<br>Aldosterone synthesis<br>Protein processing in the ER<br>Ovarian steroidogenesis<br>PPAR signalling<br>N-glycan biosynthesis<br>Sphingolipid metabolism<br>mTOR signalling | ST3GAL4<br>SIKE, IFNkappa<br>MARCKS, Arp2/3, VAV, AMPHIIm<br>Arp2/3<br>AC, MITF, CtBP<br>LDLR, SR-B1, AC<br>LDLR, SR-B1, AC<br>ERManI, Hsp40,<br>AC, LDLR, SR-B1<br>GyK, Perilipin<br>MAN1, MGAT1<br>CERS1, UGCG<br>FNIP, SGK1 |

| | | | | |
|---|---|---|---|---|
| Male genes decreased in expression | Metabolic pathways<br>cAMP signalling<br>Pathways in cancer<br>Ras signalling pathway<br>Fc gamma R mediated phagocytosis<br>Endocytosis<br>Sphingolipid signalling pathway<br>Regulations of actin cytoskeleton<br>chemokine signalling pathway<br>Ether lipid metabolism<br>cytokine-cytokine receptor pathway | PLD, NFKB<br>EPAC, VAV2, PLD, NFkappaB<br>SDF1, NfkappaB, RhoGEF, , PLD1, AML1ETC<br>NfkappaB, PLD<br>PLD, VAV<br>PLD, Rab22,<br>PLD, NfkappaB<br>VAV, RhoGEF, IRSp53<br>NfkappaB, VAV<br>PLD<br>IL1RAP | Regulation of actin cytoskeleton<br>Transcriptional misregulation in cancer<br>PI3K-AKT signalling<br>Proteoglycan in cancer<br>N-glycan biosynthesis | Arp2/3<br>AML1, CEBPalpha, ETO, PML, PLZF, RARalpha, E2A, PBX1, TEL, AML1, MLL, AF4, ENL, TLX3, TLX1, LMO2, c-Rel, Bcl-6, IgH, MAF, MMSET, PAX5, PAX8, PRCC, TMPSS2, ERG, ETV1, ETV4, ETV5, ELK4, SLC45A3, DDX5, MYCN, Menin, EWSR1, FLI1, ETV1, ERG, ETV4, FEV, ATF1, WT1, TAF15, FUS, DDIT3, PAX3, FOXO1A, SSX, SYT, ASPL, TFE3<br>GF, PKCs, YWHAE<br>Sdc-1, HGF, PKCalpha, PKC<br>MGAT5, B4GALT3 |

Table M2S6. MicroRNAs (MIR), long non-coding RNAs (LINC) and small nuclear RNAs (snoRNA) identified as differentially expressed in old females. Where transcripts were also identified as significantly (q<0.05) differentially expressed in males the locus is given, if the transcript was not identified as differentially expressed in old males the column contains NA

| Transcript IDs | locus female | locus male |
|---|---|---|
| MIR1257,TAF4 | 20:60528524-60640866 | NA |
| MIR125B1 | 11:121899062-121988132 | NA |
| MIR1287,PYROXD2 | 10:100143171-100175149 | 10:100143307-100175030 |
| MIR1304,SNORA1,SNORA18,SNORA25,SNORA32,SNORA40,SNORA8,SNORD5,TAF1D | 11:93394804-93547861 | 11:93394804-93547861 |
| MIR130B,PPIL2 | 22:22006558-22090123 | NA |
| MIR1909,REXO1 | 19:1815247-1848452 | 19:1815247-1848452 |
| MIR22HG | 17:1614775-1641893 | NA |
| MIR3605,PHC2 | 1:33772366-33896653 | NA |
| MIR3614,TRIM25 | 17:54965269-54991399 | 17:54965269-54991399 |
| MIR3671 | 1:65509411-65532186 | NA |
| MIR3917,STMN1 | 1:26210671-26233482 | NA |
| MIR423,NSRP1 | 17:27887564-28514994 | NA |
| MIR4435-1HG | 2:111953539-112268567 | NA |
| MIR4435-1HG | 2:111953539-112268567 | NA |
| MIR4517,NFATC2IP | 16:28962127-28978418 | NA |
| MIR4523,TAOK1 | 17:27679086-27878922 | NA |
| MIR4647,SLC35B2 | 6:44214823-44225291 | NA |

| | | |
|---|---|---|
| MIR4651,POR | 7:75528517-75623977 | 7:75528517-75623977 |
| MIR4680,PDCD4 | 10:112629500-112679032 | NA |
| MIR4741,RBBP8 | 18:20279443-20606451 | NA |
| MIR4745,PTBP1 | 19:797074-812327 | NA |
| MIR4750,TBC1D17 | 19:50372294-50392005 | NA |
| MIR4784,MZT2A | 2:132222472-132250316 | NA |
| MIR5187,TOMM40L | 1:161195792-161208092 | NA |
| MIR600HG,STRBP | 9:125871772-126030855 | NA |
| MIR639,TECR | 19:14625581-14676792 | NA |
| LINC00094 | 9:136890560-136933657 | NA |
| LINC00152 | 2:87754886-87907311 | NA |
| LINC00338,SEC14L1 | 17:75082797-75213179 | NA |
| LINC00342 | 2:96472293-96486935 | NA |
| LINC00630,LL0XNC01-237H1.2 | X:102024088-102161086 | NA |
| LINC00657 | 20:34633265-34638893 | NA |
| LINC00662 | 19:28175487-28475892 | 19:28175487-28475892 |
| LINC00843,PARGP1 | 10:51592079-51742743 | 10:51623416-51742596 |
| LINC00854 | 17:41363853-41383338 | NA |
| LINC00863,NUTM2D | 10:89102492-89130452 | NA |
| LINC00894 | X:149097744-149392815 | NA |
| LINC00969,MUC20,SDHAP2 | 3:195384932-195467994 | NA |
| LINC00998 | 7:112740717-112786385 | NA |
| LINC01011,NQO2,RP1-90J20.12 | 6:2988200-3024006 | NA |
| LINC01122 | 2:58654933-59290901 | NA |
| LINC01155 | X:53122891-53200096 | NA |
| snoU13 | 7:56168307-56174269 | NA |

Table M2S7. Network Analyst KEGG enriched pathways identified for significantly (q<0.05) differentially expressed transcripts with different expression levels and GC content in males and females

| Pathway | Total | Expected | Hits | P.Value | FDR |
|---|---|---|---|---|---|
| Chronic myeloid leukemia | 73 | 5.51 | 27 | 5.68E-13 | 1.23E-10 |
| Pathways in cancer | 310 | 23.4 | 60 | 1.74E-12 | 1.89E-10 |
| RNA transport | 126 | 9.5 | 35 | 3.17E-12 | 2.29E-10 |
| Cell cycle | 124 | 9.35 | 33 | 4.94E-11 | 2.68E-09 |
| Prostate cancer | 87 | 6.56 | 25 | 2.13E-09 | 9.26E-08 |
| Neurotrophin signaling pathway | 123 | 9.28 | 30 | 3.86E-09 | 0.00000014 |
| Herpes simplex infection | 103 | 7.77 | 25 | 9.02E-08 | 0.0000028 |
| ErbB signaling pathway | 87 | 6.56 | 22 | 0.000000253 | 0.00000687 |
| Protein processing in endoplasmic reticulum | 129 | 9.73 | 27 | 0.000000701 | 0.0000169 |
| NOD-like receptor signaling pathway | 49 | 3.7 | 15 | 0.00000159 | 0.0000346 |
| T cell receptor signaling pathway | 98 | 7.39 | 22 | 0.00000228 | 0.000045 |
| Shigellosis | 47 | 3.55 | 14 | 0.00000513 | 0.0000928 |
| Glioma | 65 | 4.9 | 16 | 0.0000165 | 0.000276 |
| mRNA surveillance pathway | 82 | 6.19 | 18 | 0.0000268 | 0.000416 |
| Epstein-Barr virus infection | 91 | 6.86 | 19 | 0.0000339 | 0.000491 |
| Ribosome biogenesis in eukaryotes | 55 | 4.15 | 14 | 0.0000374 | 0.000508 |
| Non-small cell lung cancer | 52 | 3.92 | 13 | 0.000087 | 0.00111 |
| Hepatitis C | 100 | 7.54 | 19 | 0.000131 | 0.00158 |
| Focal adhesion | 200 | 15.1 | 30 | 0.000175 | 0.002 |
| Bacterial invasion of epithelial cells | 56 | 4.22 | 13 | 0.000196 | 0.00213 |
| Small cell lung cancer | 80 | 6.03 | 16 | 0.000241 | 0.00249 |
| Renal cell carcinoma | 60 | 4.53 | 13 | 0.000407 | 0.00401 |
| Pancreatic cancer | 69 | 5.2 | 14 | 0.000502 | 0.00474 |
| Adherens junction | 70 | 5.28 | 14 | 0.000586 | 0.0053 |
| Pathogenic Escherichia coli infection | 35 | 2.64 | 9 | 0.000865 | 0.00751 |
| Influenza A | 107 | 8.07 | 18 | 0.000922 | 0.00769 |
| Bladder cancer | 29 | 2.19 | 8 | 0.00102 | 0.00816 |
| Apoptosis | 83 | 6.26 | 15 | 0.00115 | 0.0089 |
| Endometrial cancer | 44 | 3.32 | 10 | 0.00128 | 0.00956 |
| Melanoma | 68 | 5.13 | 13 | 0.00142 | 0.0103 |
| Legionellosis | 40 | 3.02 | 9 | 0.00239 | 0.0167 |
| Alzheimer's disease | 49 | 3.7 | 10 | 0.00301 | 0.0198 |
| Colorectal cancer | 49 | 3.7 | 10 | 0.00301 | 0.0198 |
| Toxoplasmosis | 93 | 7.01 | 15 | 0.00369 | 0.0235 |

| | | | | | |
|---|---|---|---|---|---|
| p53 signaling pathway | 68 | 5.13 | 12 | 0.00431 | 0.0267 |
| RNA degradation | 60 | 4.53 | 11 | 0.00457 | 0.0273 |
| Circadian rhythm - mammal | 22 | 1.66 | 6 | 0.00466 | 0.0273 |
| Epithelial cell signaling in Helicobacter pylori infection | 37 | 2.79 | 8 | 0.00536 | 0.0306 |
| Chemokine signaling pathway | 189 | 14.3 | 24 | 0.00742 | 0.0413 |

Table M2S8. Network analyst enriched reactome categories for transcripts identified as significantly ($q<0.05$) differentially expressed in old females that have different GC content in males and females

| Pathway | Total | Expected | Hits | P.Value | FDR |
|---|---|---|---|---|---|
| Gene Expression | 1090 | 86.9 | 216 | 6.7E-45 | 9.39E-42 |
| mRNA Splicing | 115 | 9.2 | 65 | 3.57E-42 | 1.48E-39 |
| mRNA Splicing - Major Pathway | 115 | 9.2 | 65 | 3.57E-42 | 1.48E-39 |
| Processing of Capped Intron-Containing Pre-mRNA | 119 | 9.52 | 66 | 4.21E-42 | 1.48E-39 |
| Metabolism of RNA | 339 | 27.1 | 108 | 7.38E-40 | 2.07E-37 |
| mRNA Processing | 140 | 11.2 | 69 | 1.59E-39 | 3.71E-37 |
| Nonsense Mediated Decay Enhanced by the Exon Junction Complex | 203 | 16.2 | 79 | 5.6E-36 | 9.82E-34 |
| Nonsense-Mediated Decay | 203 | 16.2 | 79 | 5.6E-36 | 9.82E-34 |
| Nonsense Mediated Decay Independent of the Exon Junction Complex | 184 | 14.7 | 74 | 7.76E-35 | 1.21E-32 |
| Metabolism of mRNA | 317 | 25.4 | 98 | 8.71E-35 | 1.22E-32 |
| Influenza Infection | 185 | 14.8 | 74 | 1.2E-34 | 1.53E-32 |
| Translation | 249 | 19.9 | 86 | 1.55E-34 | 1.81E-32 |
| Eukaryotic Translation Elongation | 186 | 14.9 | 74 | 1.86E-34 | 2E-32 |
| Peptide chain elongation | 178 | 14.2 | 72 | 4.32E-34 | 4.32E-32 |
| Influenza Life Cycle | 180 | 14.4 | 72 | 1.04E-33 | 9.73E-32 |
| GTP hydrolysis and joining of the 60S ribosomal subunit | 201 | 16.1 | 76 | 1.38E-33 | 1.08E-31 |
| 3' -UTR-mediated translational regulation | 201 | 16.1 | 76 | 1.38E-33 | 1.08E-31 |
| L13a-mediated translational silencing of Ceruloplasmin expression | 201 | 16.1 | 76 | 1.38E-33 | 1.08E-31 |
| Disease | 945 | 75.6 | 180 | 2.3E-33 | 1.7E-31 |
| Eukaryotic Translation Initiation | 209 | 16.7 | 77 | 4.09E-33 | 2.73E-31 |
| Cap-dependent Translation Initiation | 209 | 16.7 | 77 | 4.09E-33 | 2.73E-31 |
| Influenza Viral RNA Transcription and Replication | 176 | 14.1 | 70 | 1.38E-32 | 8.43E-31 |
| Viral mRNA Translation | 176 | 14.1 | 70 | 1.38E-32 | 8.43E-31 |

| | | | | | |
|---|---|---|---|---|---|
| Eukaryotic Translation Termination | 178 | 14.2 | 70 | 3.26E-32 | 1.9E-30 |
| SRP-dependent cotranslational protein targeting to membrane | 204 | 16.3 | 75 | 3.52E-32 | 1.97E-30 |
| Formation of a pool of free 40S subunits | 189 | 15.1 | 71 | 3.65E-31 | 1.97E-29 |
| Metabolism of proteins | 689 | 55.1 | 117 | 1.41E-16 | 7.12E-15 |
| RNA Polymerase II Transcription Termination | 45 | 3.6 | 25 | 1.52E-16 | 7.12E-15 |
| Post-Elongation Processing of the Transcript | 45 | 3.6 | 25 | 1.52E-16 | 7.12E-15 |
| Cleavage of Growing Transcript in the Termination Region | 45 | 3.6 | 25 | 1.52E-16 | 7.12E-15 |
| Transport of Mature mRNA derived from an Intron-Containing Transcript | 26 | 2.08 | 19 | 4.1E-16 | 1.85E-14 |
| Translation initiation complex formation | 92 | 7.36 | 35 | 5.48E-16 | 2.4E-14 |
| Activation of the mRNA upon binding of the cap-binding complex and eIFs, and subsequent binding to 43S | 93 | 7.44 | 35 | 8.14E-16 | 3.39E-14 |
| Formation of the ternary complex, and subsequently, the 43S complex | 83 | 6.64 | 33 | 8.21E-16 | 3.39E-14 |
| Ribosomal scanning and start codon recognition | 91 | 7.28 | 34 | 2.76E-15 | 1.1E-13 |
| Transport of Mature Transcript to Cytoplasm | 30 | 2.4 | 19 | 2.51E-14 | 9.77E-13 |
| mRNA Splicing - Minor Pathway | 45 | 3.6 | 23 | 2.88E-14 | 1.09E-12 |
| mRNA 3'-end processing | 36 | 2.88 | 20 | 1.77E-13 | 6.37E-12 |
| Post-Elongation Processing of Intron-Containing pre-mRNA | 36 | 2.88 | 20 | 1.77E-13 | 6.37E-12 |
| RNA Polymerase II Transcription | 107 | 8.56 | 33 | 3.76E-12 | 1.32E-10 |
| Signaling by the B Cell Receptor (BCR) | 199 | 15.9 | 46 | 1.91E-11 | 6.53E-10 |
| Activation of BH3-only proteins | 24 | 1.92 | 14 | 3.44E-10 | 1.15E-08 |
| Intrinsic Pathway for Apoptosis | 37 | 2.96 | 17 | 6.14E-10 | 0.00000002 |
| Transcription | 149 | 11.9 | 36 | 8.92E-10 | 2.84E-08 |
| Signaling by EGFR in Cancer | 181 | 14.5 | 40 | 1.75E-09 | 5.45E-08 |
| Signaling by constitutively active EGFR | 19 | 1.52 | 12 | 1.82E-09 | 5.55E-08 |
| Signaling by ERBB2 | 164 | 13.1 | 37 | 0.000000004 | 0.000000119 |
| Apoptosis | 158 | 12.6 | 36 | 4.93E-09 | 0.000000144 |
| Downstream Signaling Events Of B Cell Receptor (BCR) | 173 | 13.8 | 38 | 5.43E-09 | 0.000000155 |
| Signaling by ERBB4 | 152 | 12.2 | 35 | 6.05E-09 | 0.00000017 |
| Downstream signal transduction | 163 | 13 | 36 | 1.19E-08 | 0.000000328 |
| Signaling by FGFR in disease | 178 | 14.2 | 38 | 1.25E-08 | 0.000000337 |
| Signaling by EGFR | 179 | 14.3 | 38 | 1.47E-08 | 0.000000389 |
| Signalling by NGF | 290 | 23.2 | 52 | 1.54E-08 | 0.000000399 |
| Signaling by FGFR | 162 | 13 | 35 | 3.48E-08 | 0.000000887 |

| | | | | | |
|---|---|---|---|---|---|
| Signaling by SCF-KIT | 142 | 11.4 | 32 | 4.77E-08 | 0.00000119 |
| Signaling by NOTCH | 95 | 7.6 | 25 | 5.85E-08 | 0.00000144 |
| snRNP Assembly | 24 | 1.92 | 12 | 6.72E-08 | 0.0000016 |
| Metabolism of non-coding RNA | 24 | 1.92 | 12 | 6.72E-08 | 0.0000016 |
| Activation of BAD and translocation to mitochondria | 17 | 1.36 | 10 | 0.000000115 | 0.00000268 |
| Signaling by PDGF | 189 | 15.1 | 37 | 0.000000211 | 0.00000484 |
| Constitutive Signaling by NOTCH1 HD+PEST Domain Mutants | 52 | 4.16 | 17 | 0.000000272 | 0.00000614 |
| DAP12 signaling | 164 | 13.1 | 33 | 0.000000504 | 0.0000112 |
| GAB1 signalosome | 106 | 8.48 | 25 | 0.000000578 | 0.0000125 |
| PI3K/AKT activation | 106 | 8.48 | 25 | 0.000000578 | 0.0000125 |
| Downstream signaling of activated FGFR | 150 | 12 | 31 | 0.000000609 | 0.0000129 |
| NGF signalling via TRKA from the plasma membrane | 207 | 16.6 | 38 | 0.000000798 | 0.0000167 |
| NOTCH1 Intracellular Domain Regulates Transcription | 50 | 4 | 16 | 0.00000085 | 0.0000175 |
| PI3K events in ERBB4 signaling | 103 | 8.24 | 24 | 0.00000123 | 0.0000237 |
| PIP3 activates AKT signaling | 103 | 8.24 | 24 | 0.00000123 | 0.0000237 |
| PI-3K cascade | 103 | 8.24 | 24 | 0.00000123 | 0.0000237 |
| PI3K/AKT Signaling in Cancer | 103 | 8.24 | 24 | 0.00000123 | 0.0000237 |
| PI3K events in ERBB2 signaling | 103 | 8.24 | 24 | 0.00000123 | 0.0000237 |
| SLBP independent Processing of Histone Pre-mRNAs | 10 | 0.8 | 7 | 0.00000195 | 0.000037 |
| Constitutive Signaling by NOTCH1 PEST Domain Mutants | 59 | 4.72 | 17 | 0.00000202 | 0.0000378 |
| G0 and Early G1 | 27 | 2.16 | 11 | 0.00000309 | 0.0000569 |
| Signaling by NOTCH1 t(7;9)(NOTCH1:M1580_K2555) Translocation Mutant | 74 | 5.92 | 19 | 0.00000346 | 0.0000584 |
| Signaling by NOTCH1 in Cancer | 74 | 5.92 | 19 | 0.00000346 | 0.0000584 |
| Signaling by NOTCH1 PEST Domain Mutants in Cancer | 74 | 5.92 | 19 | 0.00000346 | 0.0000584 |
| FBXW7 Mutants and NOTCH1 in Cancer | 74 | 5.92 | 19 | 0.00000346 | 0.0000584 |
| Signaling by NOTCH1 HD Domain Mutants in Cancer | 74 | 5.92 | 19 | 0.00000346 | 0.0000584 |
| Signaling by NOTCH1 HD+PEST Domain Mutants in Cancer | 74 | 5.92 | 19 | 0.00000346 | 0.0000584 |
| Signaling by NOTCH1 | 74 | 5.92 | 19 | 0.00000346 | 0.0000584 |
| Membrane Trafficking | 203 | 16.2 | 36 | 0.00000363 | 0.0000607 |
| SLBP Dependent Processing of Replication-Dependent Histone Pre-mRNAs | 11 | 0.88 | 7 | 0.000005 | 0.0000825 |
| DAP12 interactions | 182 | 14.6 | 33 | 0.00000569 | 0.0000927 |
| Pre-NOTCH Transcription and Translation | 12 | 0.96 | 7 | 0.0000112 | 0.00018 |

| | | | | | |
|---|---|---|---|---|---|
| Signaling by Interleukins | 116 | 9.28 | 24 | 0.0000113 | 0.00018 |
| Antigen Activates B Cell Receptor Leading to Generation of Second Messengers | 32 | 2.56 | 11 | 0.0000211 | 0.000332 |
| Innate Immune System | 521 | 41.7 | 68 | 0.0000241 | 0.000375 |
| Interleukin-3, 5 and GM-CSF signaling | 51 | 4.08 | 14 | 0.0000298 | 0.00046 |
| HIV Infection | 214 | 17.1 | 35 | 0.0000308 | 0.00047 |
| Regulatory RNA pathways | 28 | 2.24 | 10 | 0.0000344 | 0.000519 |
| Mitotic G1-G1/S phases | 140 | 11.2 | 26 | 0.0000366 | 0.000539 |
| Processing of Capped Intronless Pre-mRNA | 23 | 1.84 | 9 | 0.0000369 | 0.000539 |
| Post-Elongation Processing of Intronless pre-mRNA | 23 | 1.84 | 9 | 0.0000369 | 0.000539 |
| Nephrin interactions | 24 | 1.92 | 9 | 0.0000549 | 0.000793 |
| Post-transcriptional Silencing By Small RNAs | 7 | 0.56 | 5 | 0.0000589 | 0.000843 |
| Cell Cycle | 508 | 40.6 | 65 | 0.0000689 | 0.000975 |
| Signalling to ERKs | 37 | 2.96 | 11 | 0.0000967 | 0.00136 |
| Translocation of GLUT4 to the Plasma Membrane | 71 | 5.68 | 16 | 0.000114 | 0.00158 |
| Fcgamma receptor (FCGR) dependent phagocytosis | 86 | 6.88 | 18 | 0.00012 | 0.00165 |
| Adaptive Immune System | 654 | 52.3 | 78 | 0.000141 | 0.00191 |
| Regulation of signaling by CBL | 22 | 1.76 | 8 | 0.000185 | 0.00249 |
| Transport of the SLBP independent Mature mRNA | 5 | 0.4 | 4 | 0.00019 | 0.00249 |
| Loss of Function of FBXW7 in Cancer and NOTCH1 Signaling | 5 | 0.4 | 4 | 0.00019 | 0.00249 |
| Constitutive PI3K/AKT Signaling in Cancer | 89 | 7.12 | 18 | 0.00019 | 0.00249 |
| Regulation of KIT signaling | 17 | 1.36 | 7 | 0.000192 | 0.0025 |
| Signaling by Robo receptor | 34 | 2.72 | 10 | 0.000222 | 0.00285 |
| Axon guidance | 292 | 23.4 | 41 | 0.000236 | 0.003 |
| Apoptosis induced DNA fragmentation | 13 | 1.04 | 6 | 0.000268 | 0.0033 |
| Activation of DNA fragmentation factor | 13 | 1.04 | 6 | 0.000268 | 0.0033 |
| PECAM1 interactions | 13 | 1.04 | 6 | 0.000268 | 0.0033 |
| AKT phosphorylates targets in the cytosol | 13 | 1.04 | 6 | 0.000268 | 0.0033 |
| Chromosome Maintenance | 124 | 9.92 | 22 | 0.000286 | 0.00349 |
| Host Interactions of HIV factors | 141 | 11.3 | 24 | 0.000295 | 0.00356 |
| Cytokine Signaling in Immune system | 286 | 22.9 | 40 | 0.000305 | 0.00365 |
| Small Interfering RNA (siRNA) Biogenesis | 9 | 0.72 | 5 | 0.000309 | 0.00367 |
| p75 NTR receptor-mediated signalling | 85 | 6.8 | 17 | 0.000328 | 0.00386 |

| | | | | | |
|---|---|---|---|---|---|
| trans-Golgi Network Vesicle Budding | 64 | 5.12 | 14 | 0.000418 | 0.00485 |

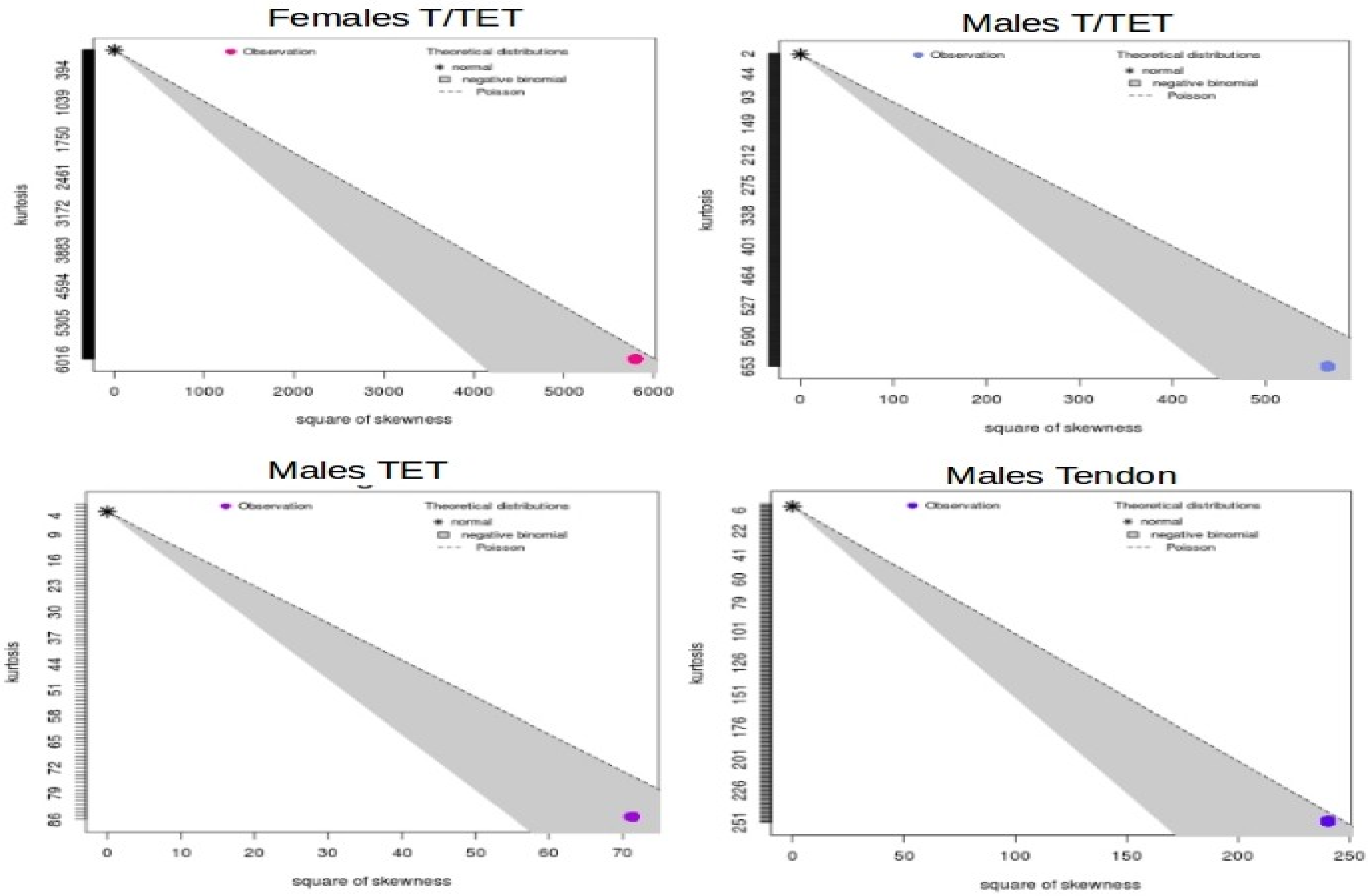


Figure M2S1 Cullen and Frey graphs of data distributions for male and female tendon tissues analysed using separate CuffDiff analysis (method 2). Males were assessed for tendon, tissue engineered tendon and a mixture of tendon tissue engineered tendon and tendon (mixed), females were assessed based on young tissue engineered tendon and old tendon because these were the only samples available.

Significant Gene Ontology categories represented by genes increased in expression in old males

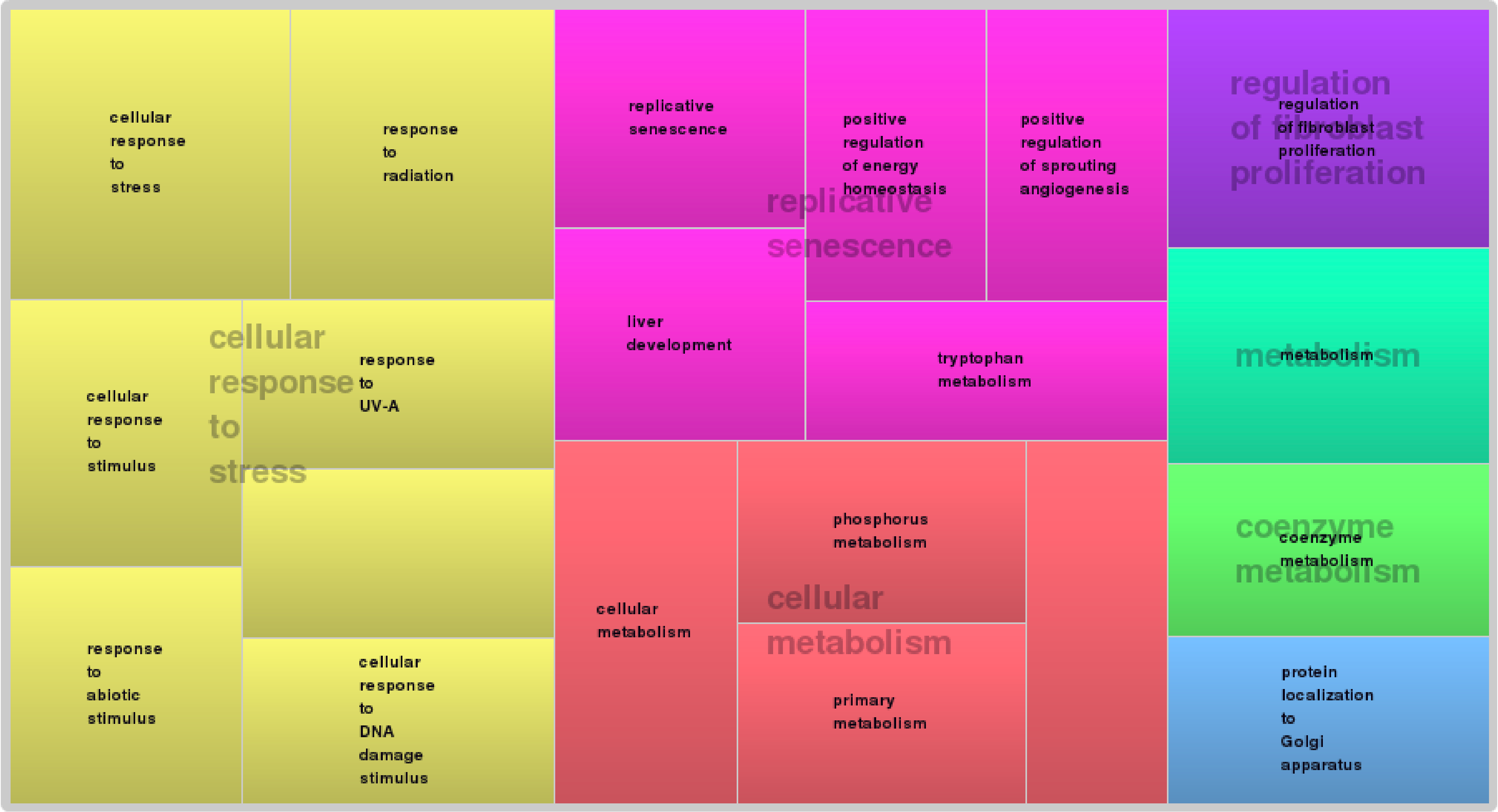


Figure M2S2 REVIGO output TreeMap of Gene Ontology Biological Process terms over-represented by genes significantly increased in expression in old males (n=21) identified using using mixed tendon and tissue engineered tendon RNASeq samples. Maps are coloured by category and sized by log10 p-value

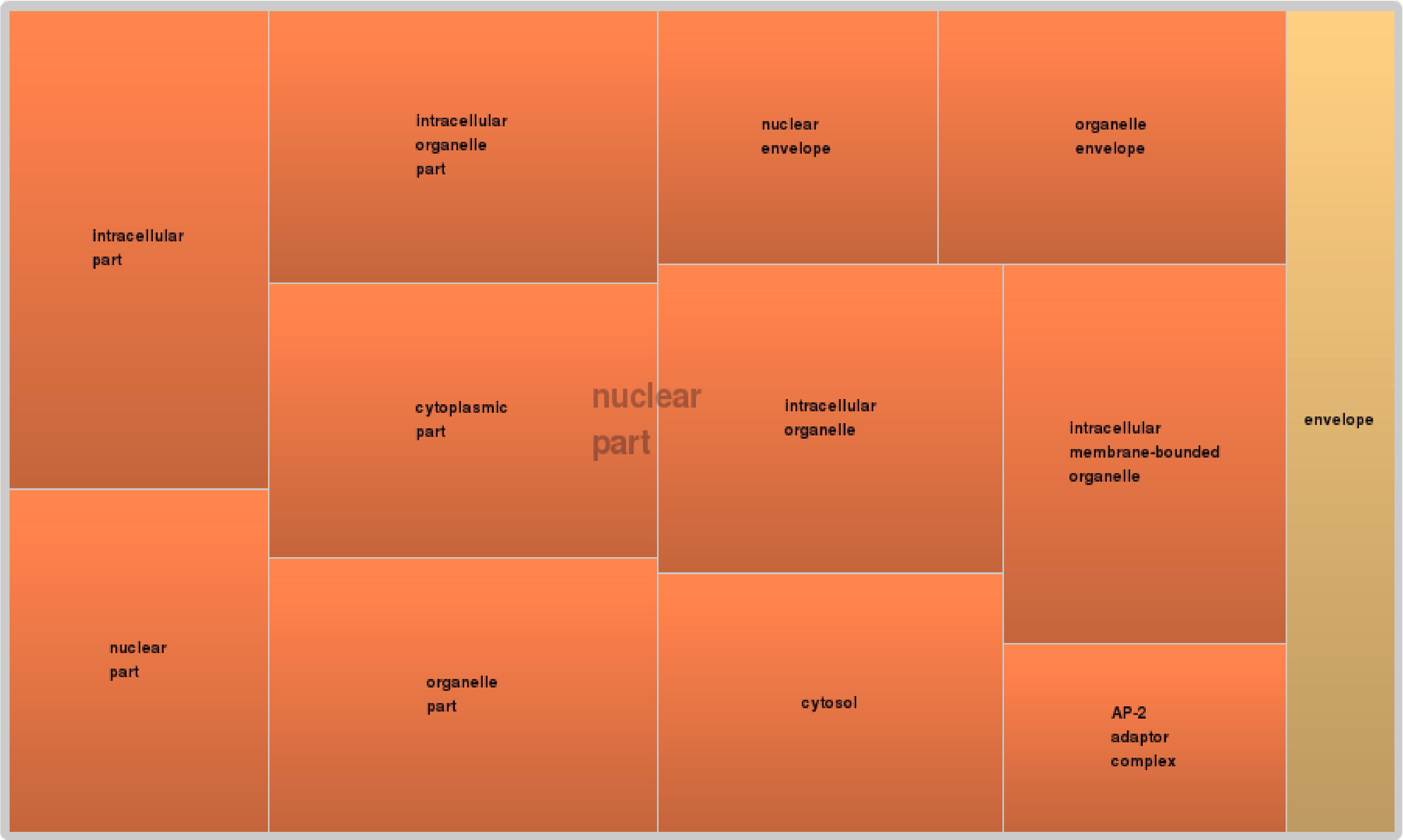


Figure M2S3 REVIGO output TreeMap of Gene Ontology Cellular Component terms over-represented by genes significantly increased in expression in old males (n=12) cells identified using using mixed tendon and tissue engineered tendon RNASeq samples. Coloured by category and sized by log10 p-value

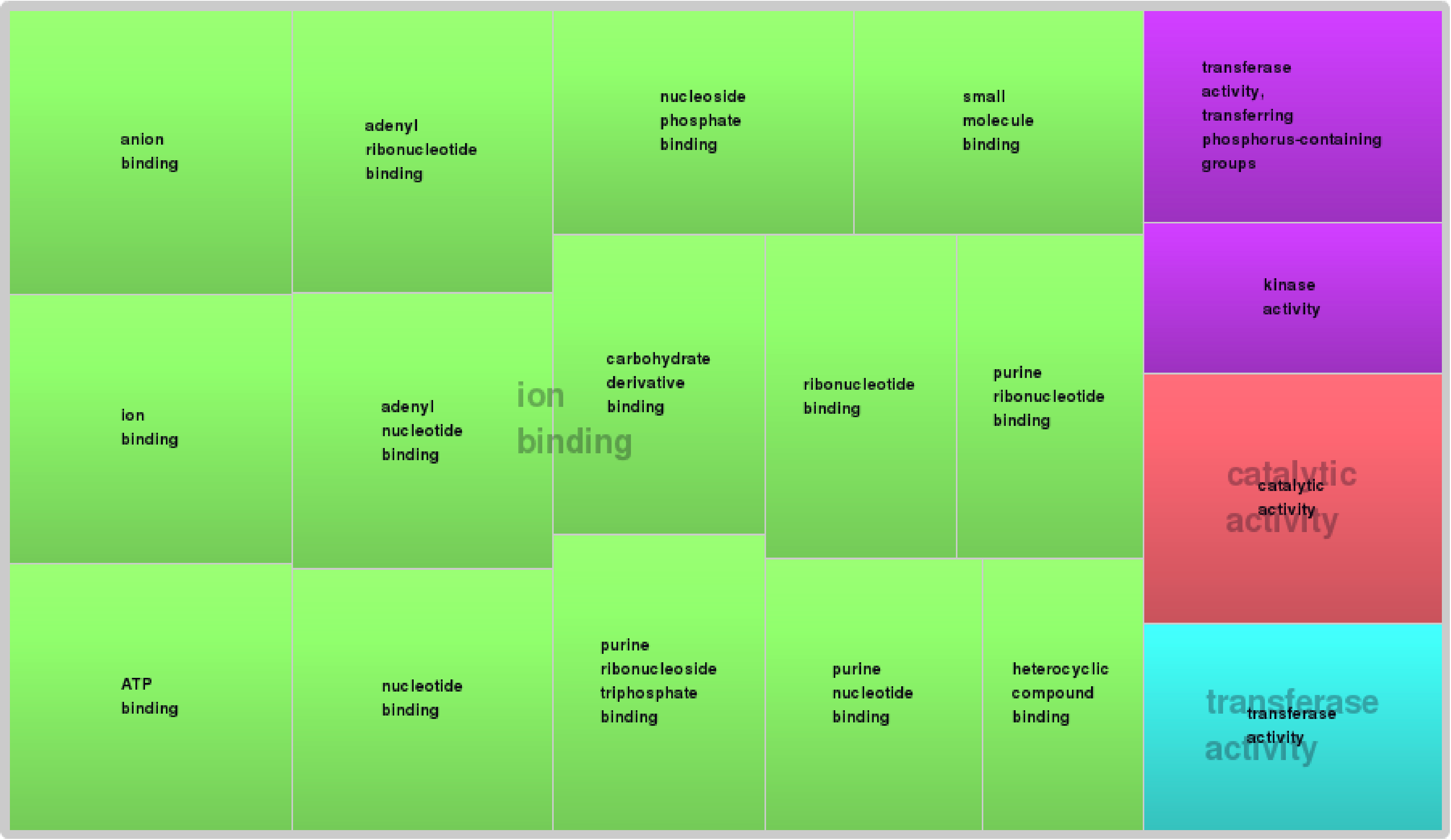


Figure M2S4 REVIGO output TreeMap of Gene Ontology Molecular Function terms over-represented by genes significantly increased in expression in old males (n=12) cells identified using using mixed tendon and tissue engineered tendon RNASeq samples. Coloured by category and sized by log10 p-value

Significant Gene Ontology categories represented by genes increased in expression in old females

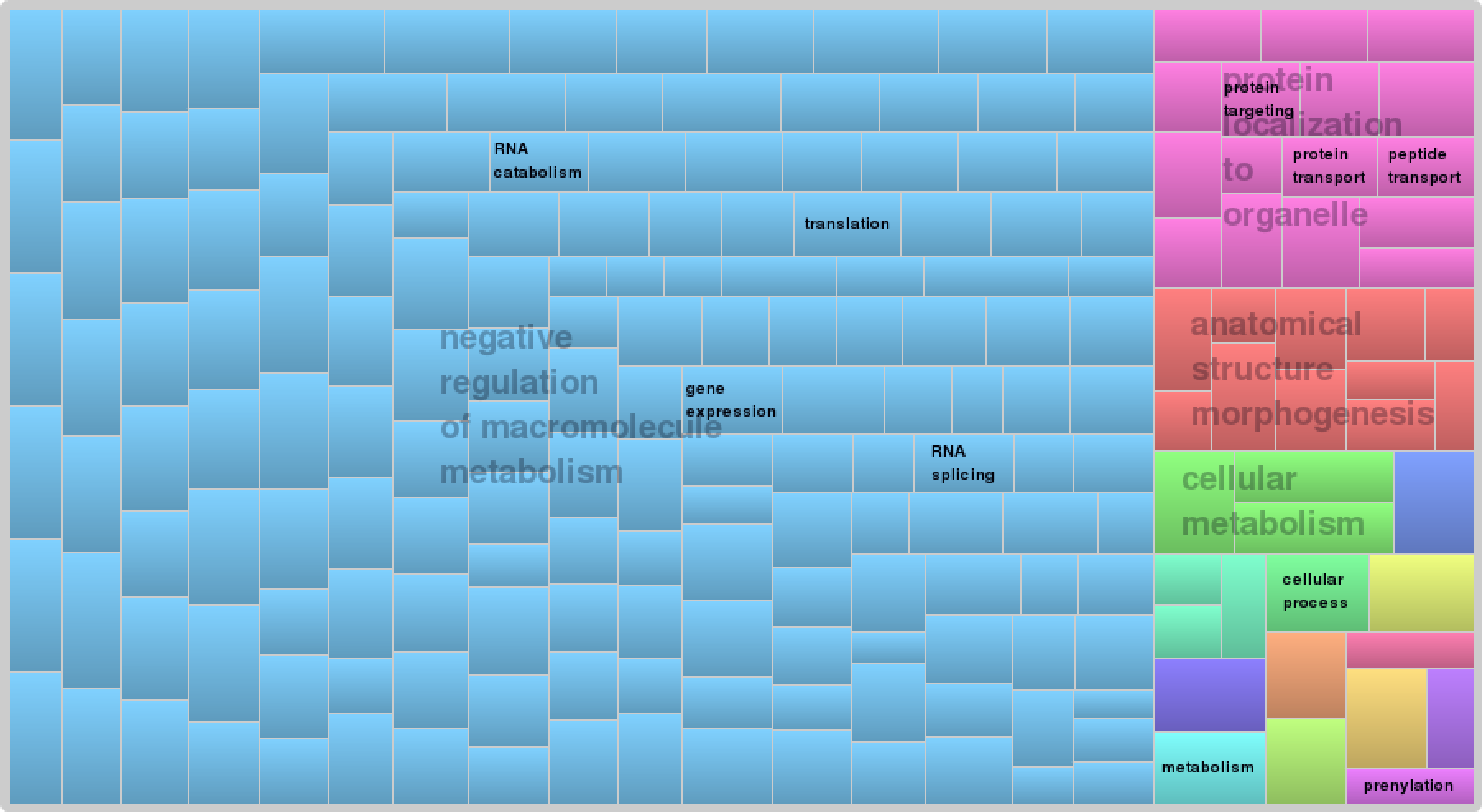


Figure M2S4 REVIGO output TreeMap of Gene Ontology Biological Process terms over-represented by genes significantly increased in expression in old females (n=273) identified using RNASeq. Coloured by category and sized by log10 p-value

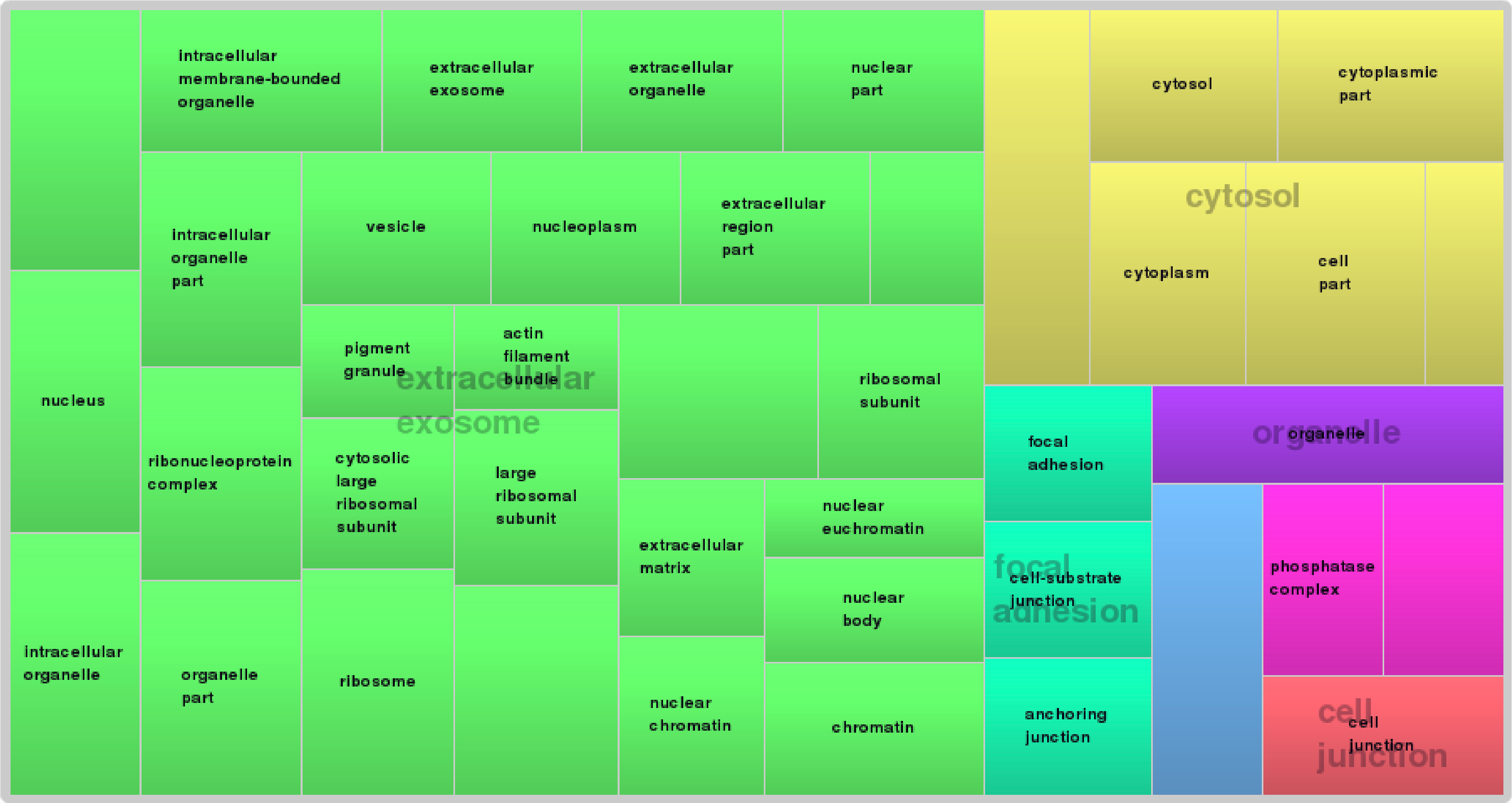


Figure M2S5 REVIGO output tree map of Gene Ontology Cellular Components terms (n=45) over-represented by genes significantly increased in expression in old female cells identified using RNAseq Coloured by category and sized by log10 p-value

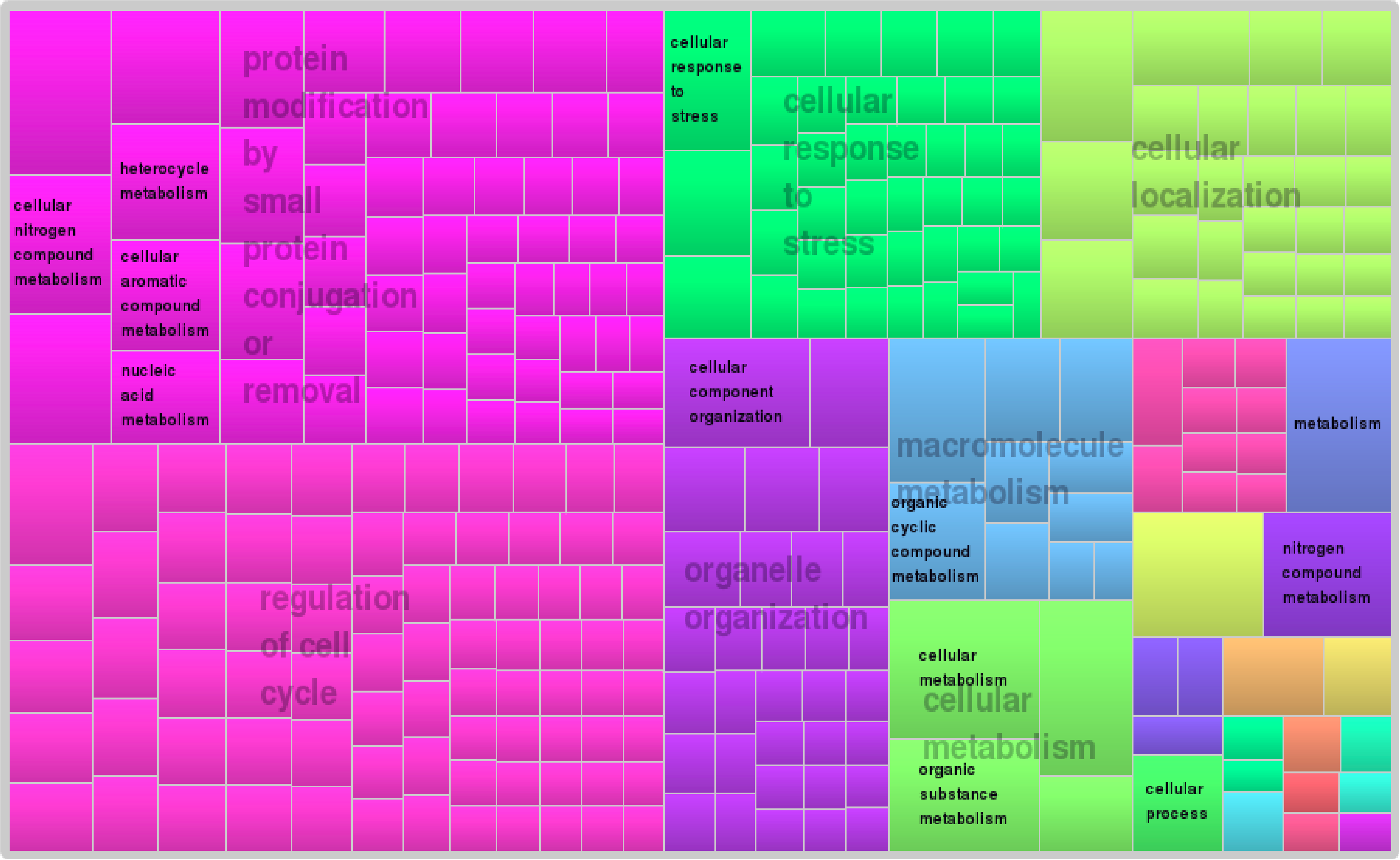


Figure M2S6 REVIGO output tree map of Gene Ontology Molecular Function terms (n=57) over-represented by genes significantly increased in expression in old female cells identified using RNAseq Coloured by category and sized by log10 p-value

Significant Gene Ontology categories represented by genes decreased in expression in old females

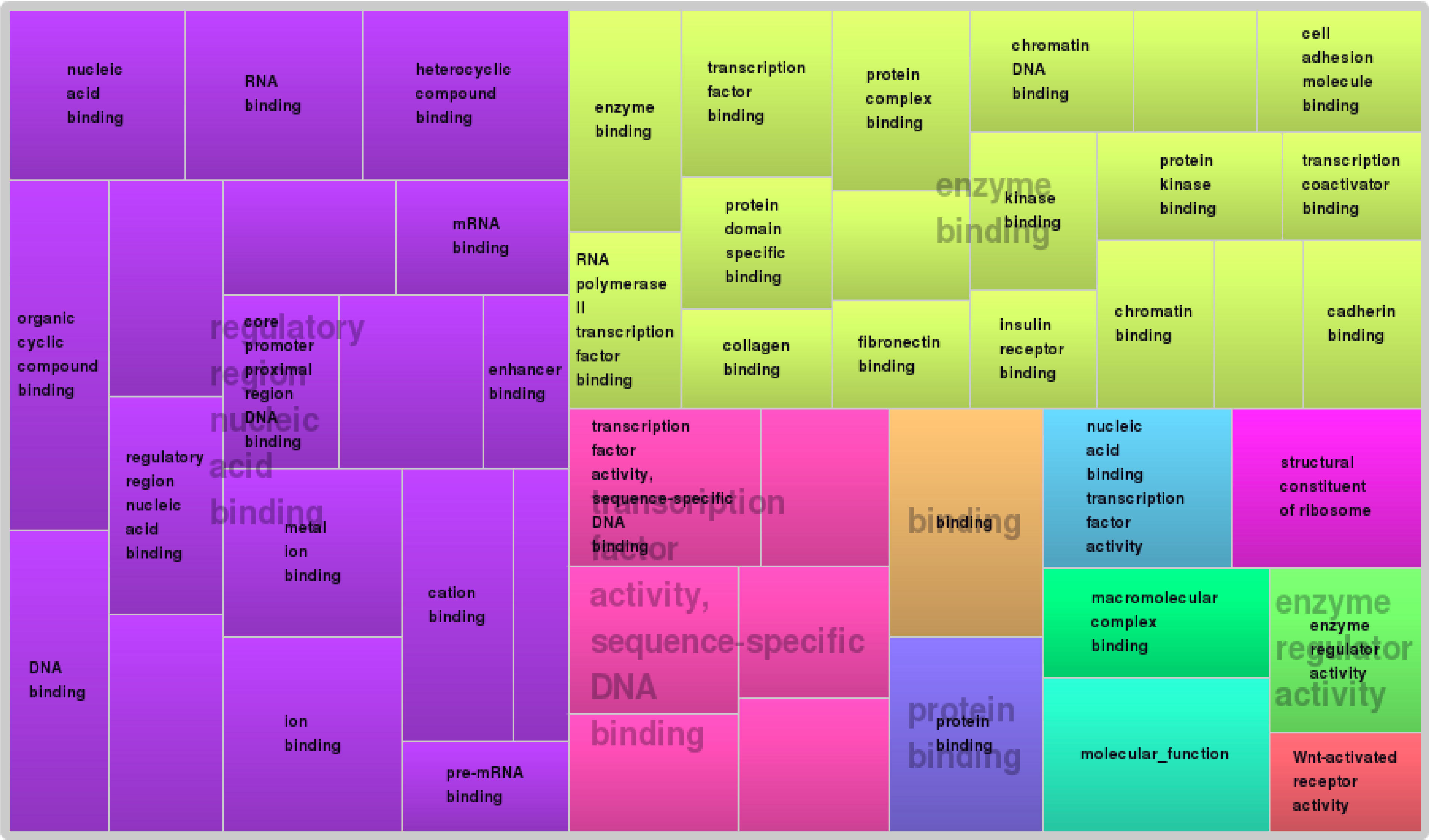


Figure M2S7 REVIGO output TreeMap of Gene Ontology Biological Process terms over-represented by genes significantly decreased in expression in old females (n=527) cells identified using RNASeq. Coloured by category and sized by log10 p-value

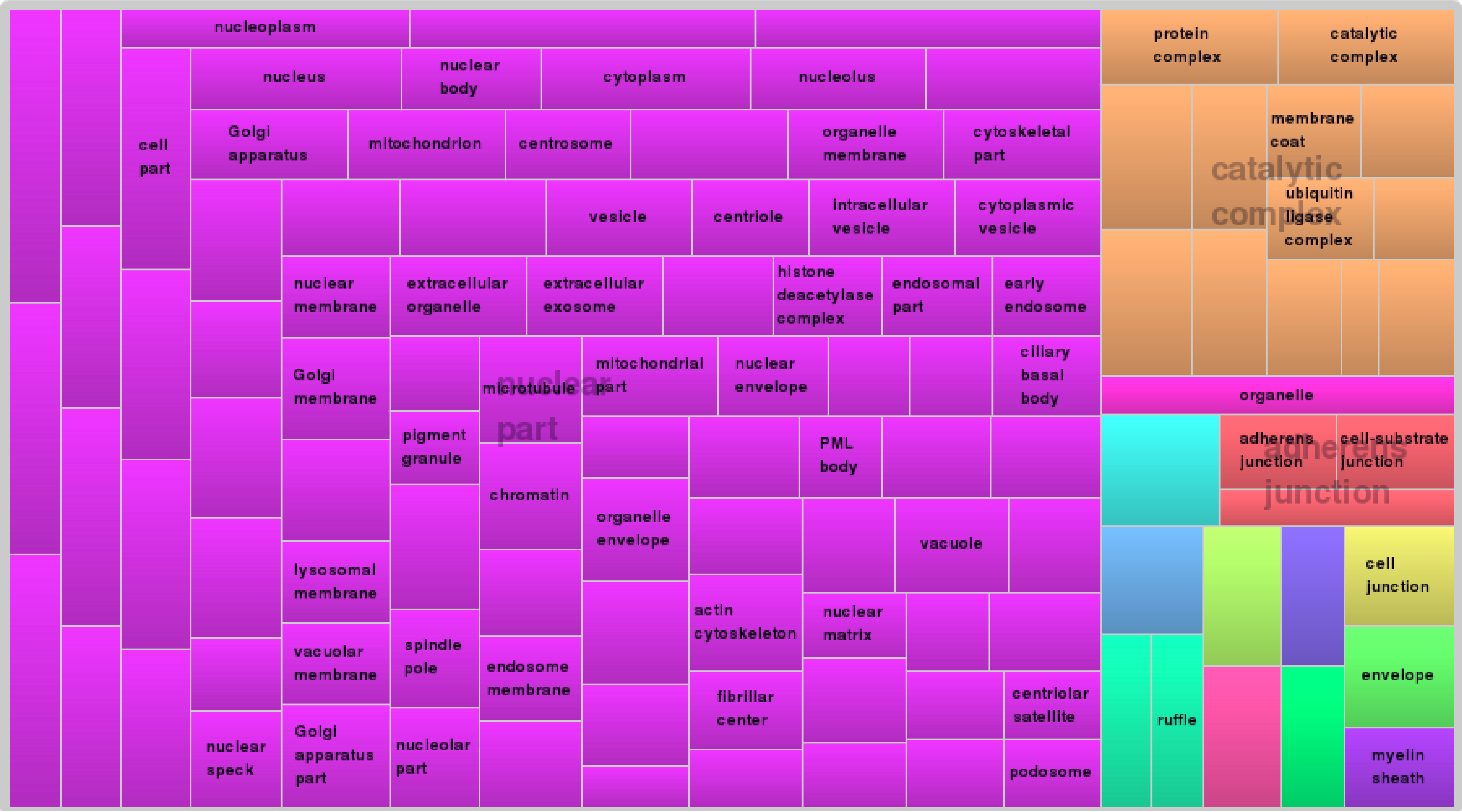


Figure M2S8 REVIGO output TreeMap of Gene Ontology Cellular Component terms over-represented by genes significantly decreased in expression in old females (n=129) cells identified using RNASeq. Coloured by category and sized by log10 p-value

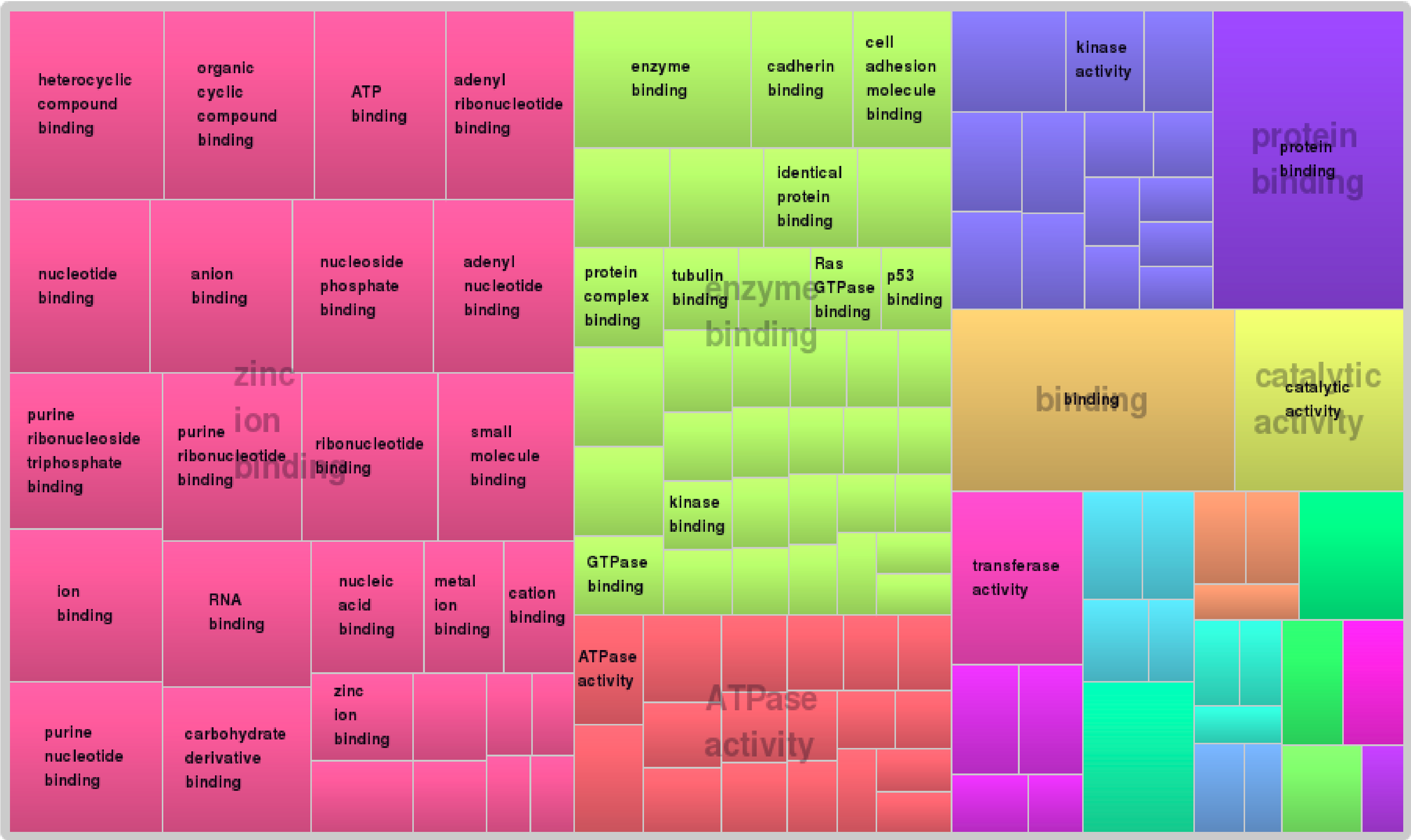


Figure M2S9 REVIGO output TreeMap of Gene Ontology Molecular Function terms over-represented by genes significantly decreased in expression in old females (n=130) cells identified using RNASeq. Coloured by category and sized by log10 p-value

Significant Gene Ontology categories represented by genes decreased in expression in old males

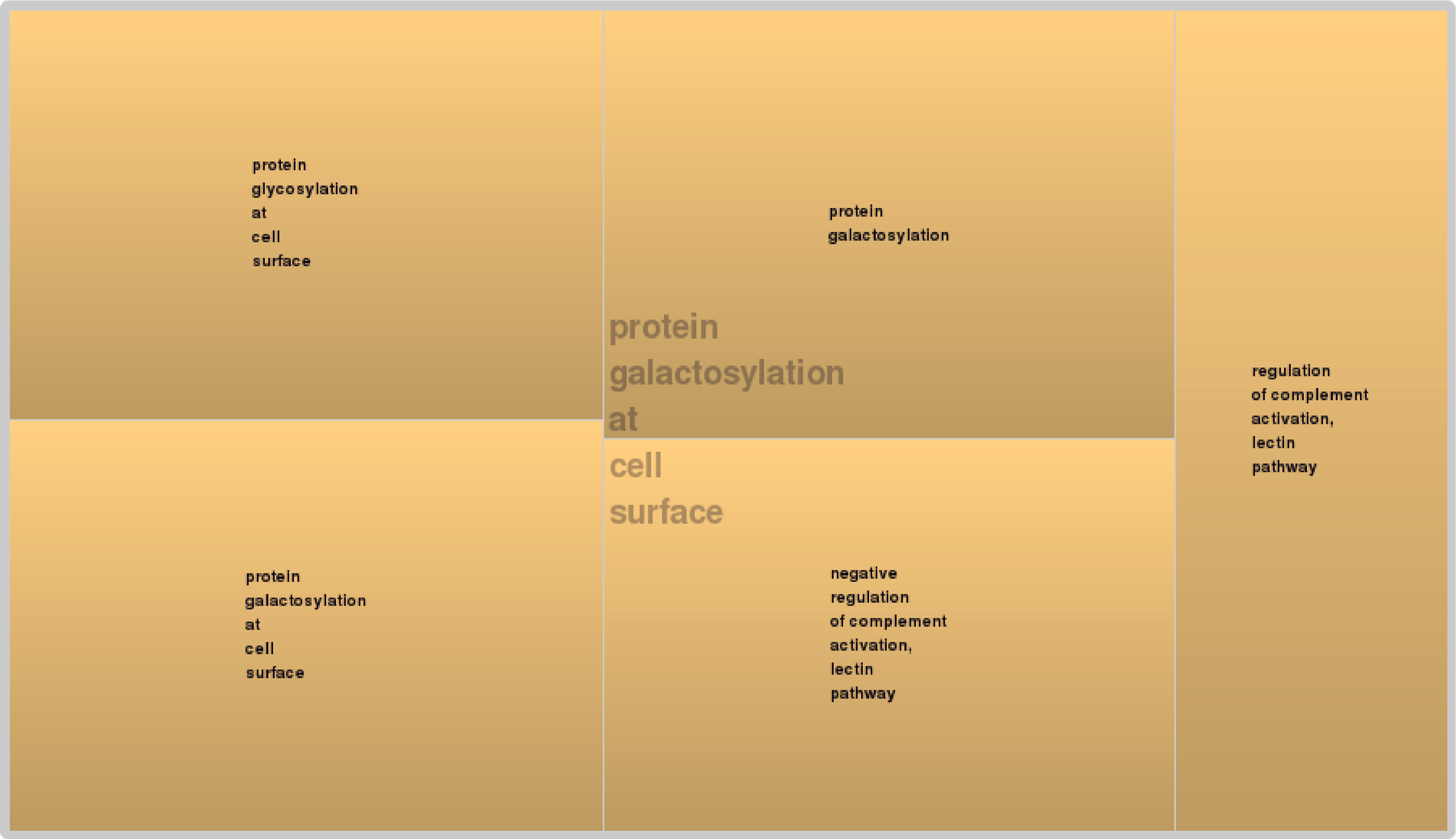


Figure M2S10 REVIGO output TreeMap of Gene Ontology Biological Process terms over-represented by genes significantly decreased in expression in old males (n=5) cells identified using using mixed tendon and tissue engineered tendon RNASeq samples. Coloured by category and sized by log10 p-value

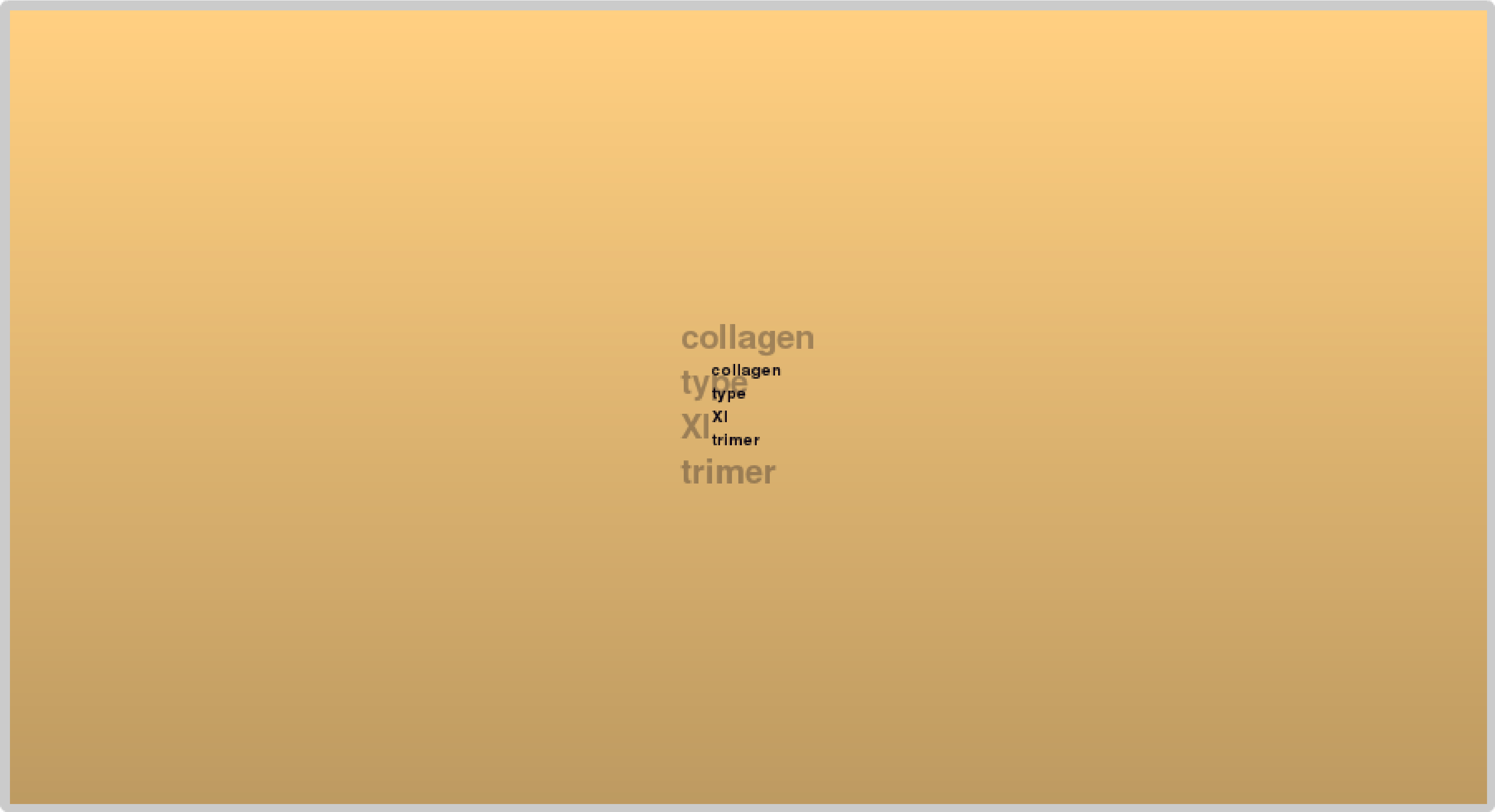


Figure M2S11 REVIGO output TreeMap of Gene Ontology Cellular Component terms over-represented by genes significantly decreased in expression in old males (n=1) cells identified using using mixed tendon and tissue engineered tendon RNASeq samples.  Coloured by category and sized by log10 p-value

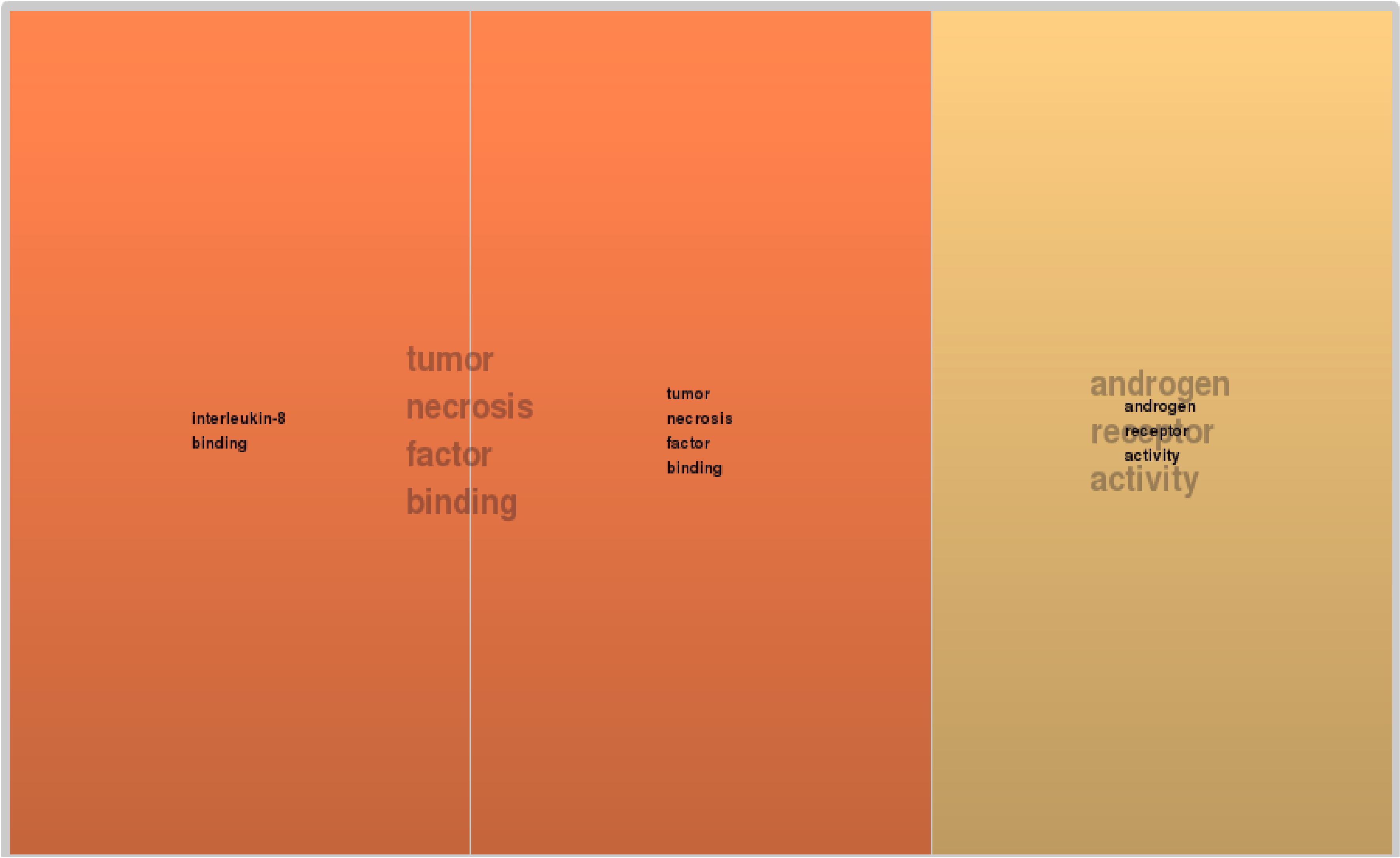


Figure M2S12 REVIGO output TreeMap of Gene Ontology Molecular Function terms over-represented by genes significantly decreased in expression in old males (n=3) cells identified using mixed tendon and tissue engineered tendon RNASeq samples.  Coloured by category and sized by log10 p-value

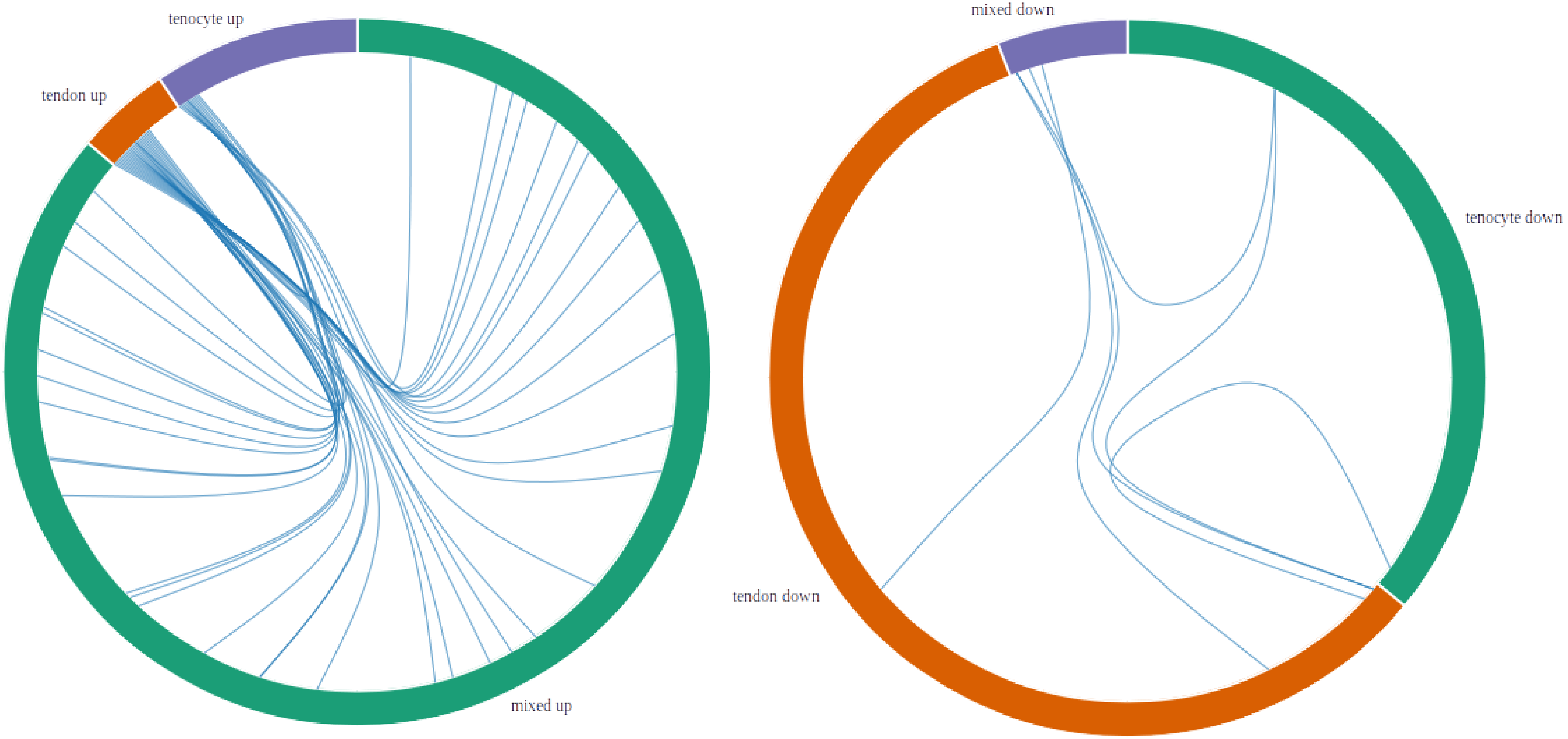


Figure M2S13 Chord diagrams generated in Network analyst showing intersecting genes that were identified as up (left) and down (right) regulated in tendon tissue (tendon), tissue engineered tendon (tenocyte) and when a mixture of tendon and tissue engineered tendon were analysed (mixed).

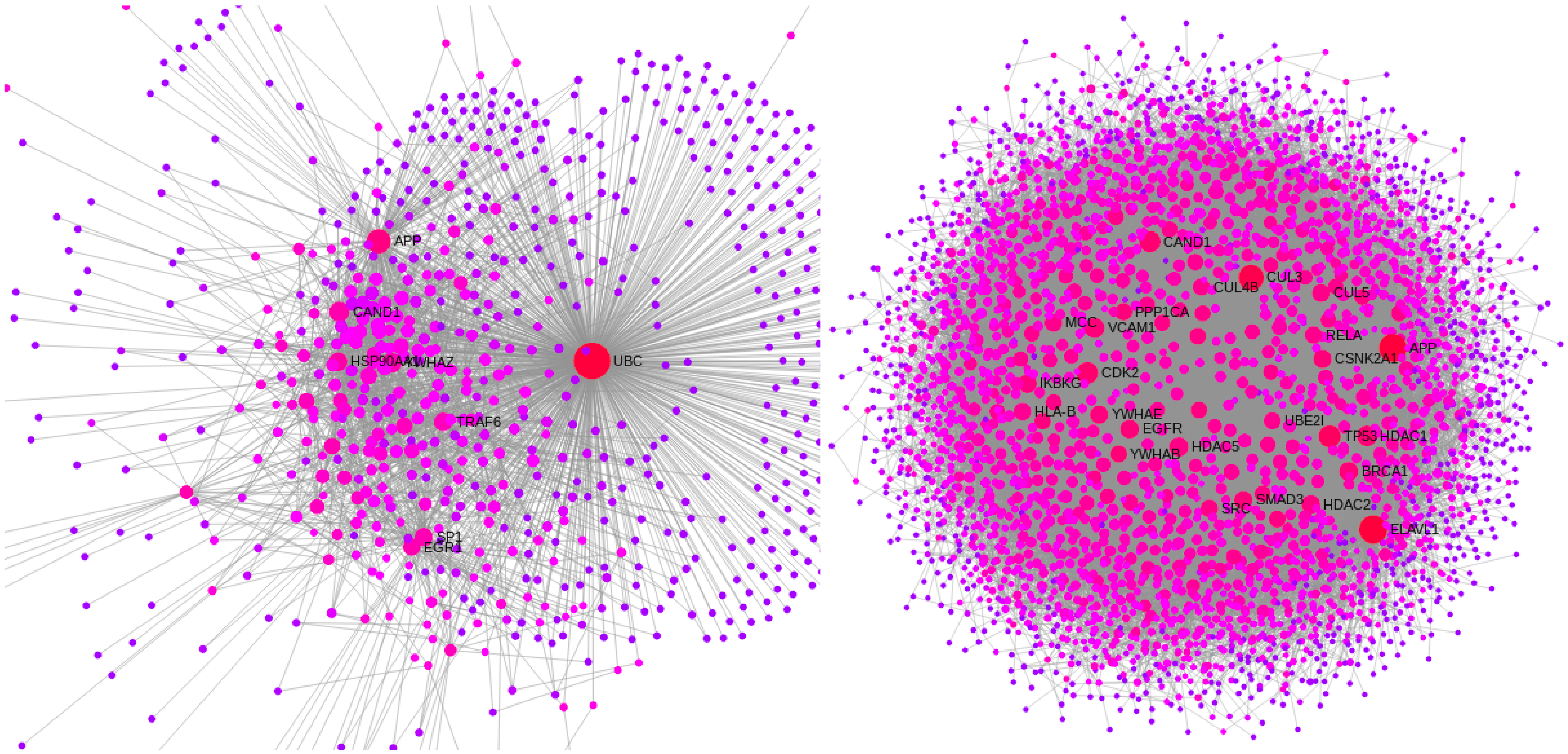


Figure M2S14 Network Analyst Topology view of zero order network identified by genes significantly (q<0.05) increased (left) and decreased (right) in expression in old females . Genes are coloured according to connectivity and significance, red nodes have the highest degree and betweenness, pink nodes have medium connectivity and purple nodes have the lowest connectivity. The most significant nodes are labelled.

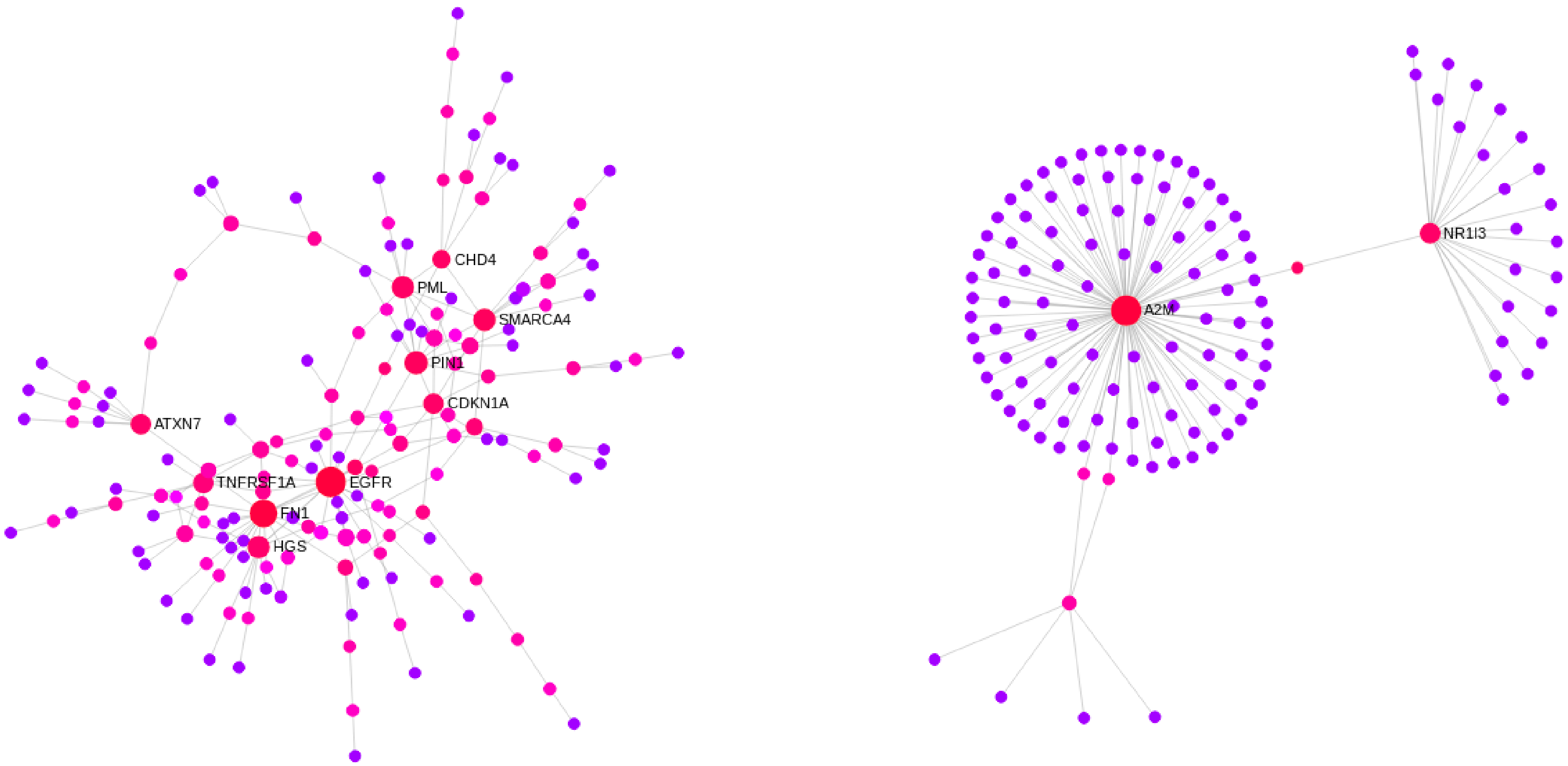


Figure M2S15 Network Analyst Topology view of the zero order network identified by genes significantly (q<0.05) increased (left) and decreased in expression in old males when tendon and tissue engineered tendon are mixed. Genes are coloured according to connectivity and significance, red nodes have the highest degree and betweenness, pink nodes have medium connectivity and purple nodes have the lowest connectivity. The most significant nodes are labelled.

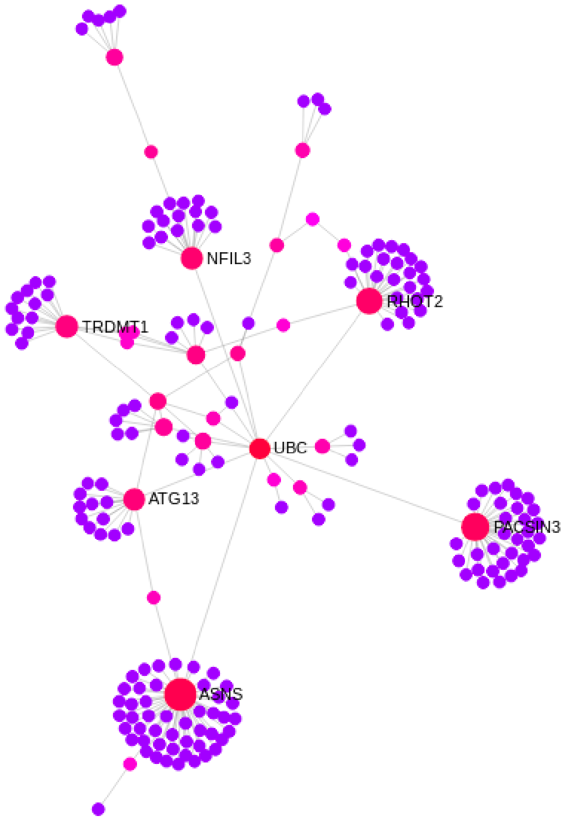


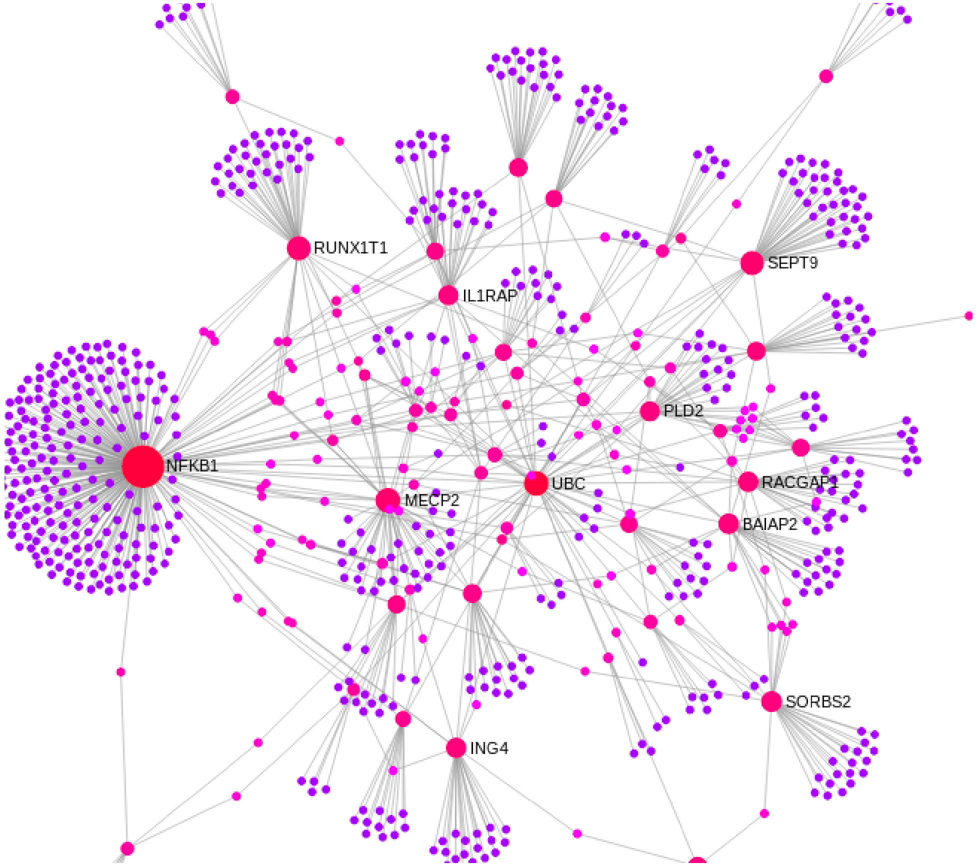


Figure M2S16 Network Analyst Topology view of the first order network identified from genes significantly (q<0.05) increased (left) and decreased in expression in old male tendon when analysed separately from tissue engineered tendon. Genes are coloured according to connectivity and significance, red nodes have the highest degree and betweenness, pink nodes have medium connectivity and purple nodes have the lowest connectivity. The most significant nodes are labelled.

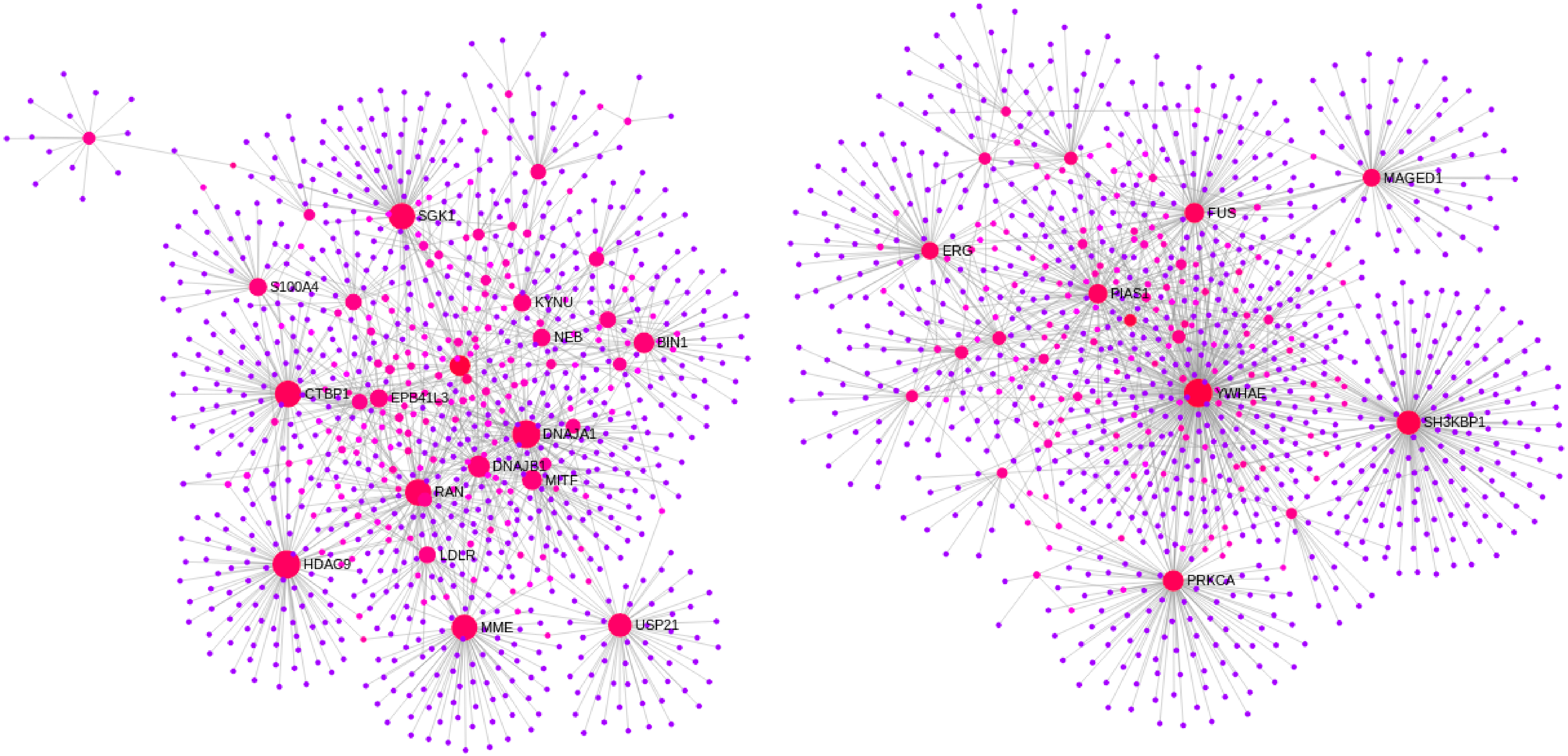


Figure M2S17 Network Analyst Topology view of the first order network identified by genes significantly (q<0.05) increased (left) and decreased in expression in old male tissue engineered tendon when analysed separately from tendon. Genes are coloured according to connectivity and significance, red nodes have the highest degree and betweenness, pink nodes have medium connectivity and purple nodes have the lowest connectivity. The most significant nodes are labelled.

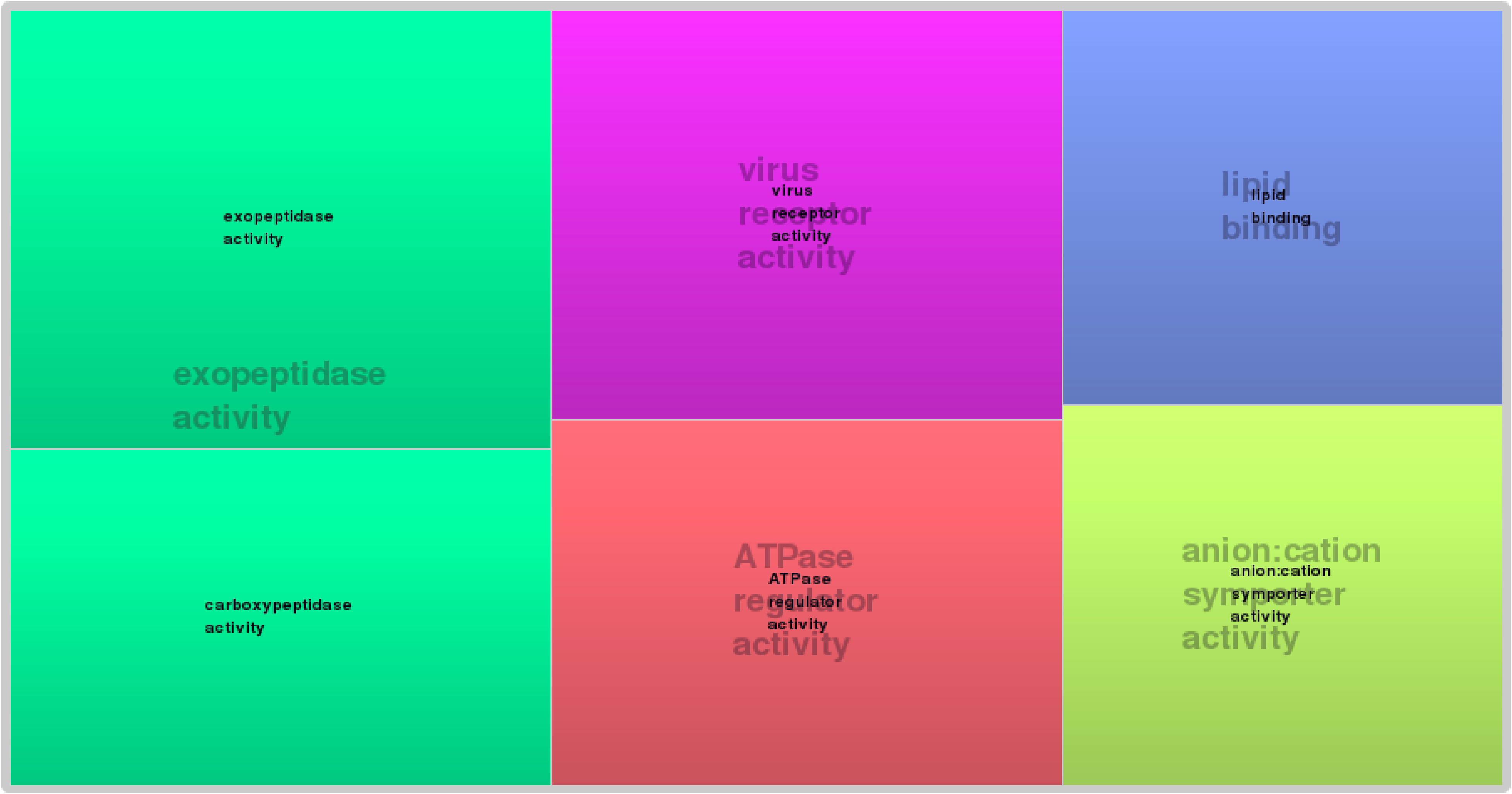


Figure M2S18 REVIGO output TreeMap of Gene Ontology Biological Process terms over-represented by genes significantly increased in expression in old males tissue engineered tendon (n=33) RNASeq samples. Maps are coloured by category and sized by log10 p-value

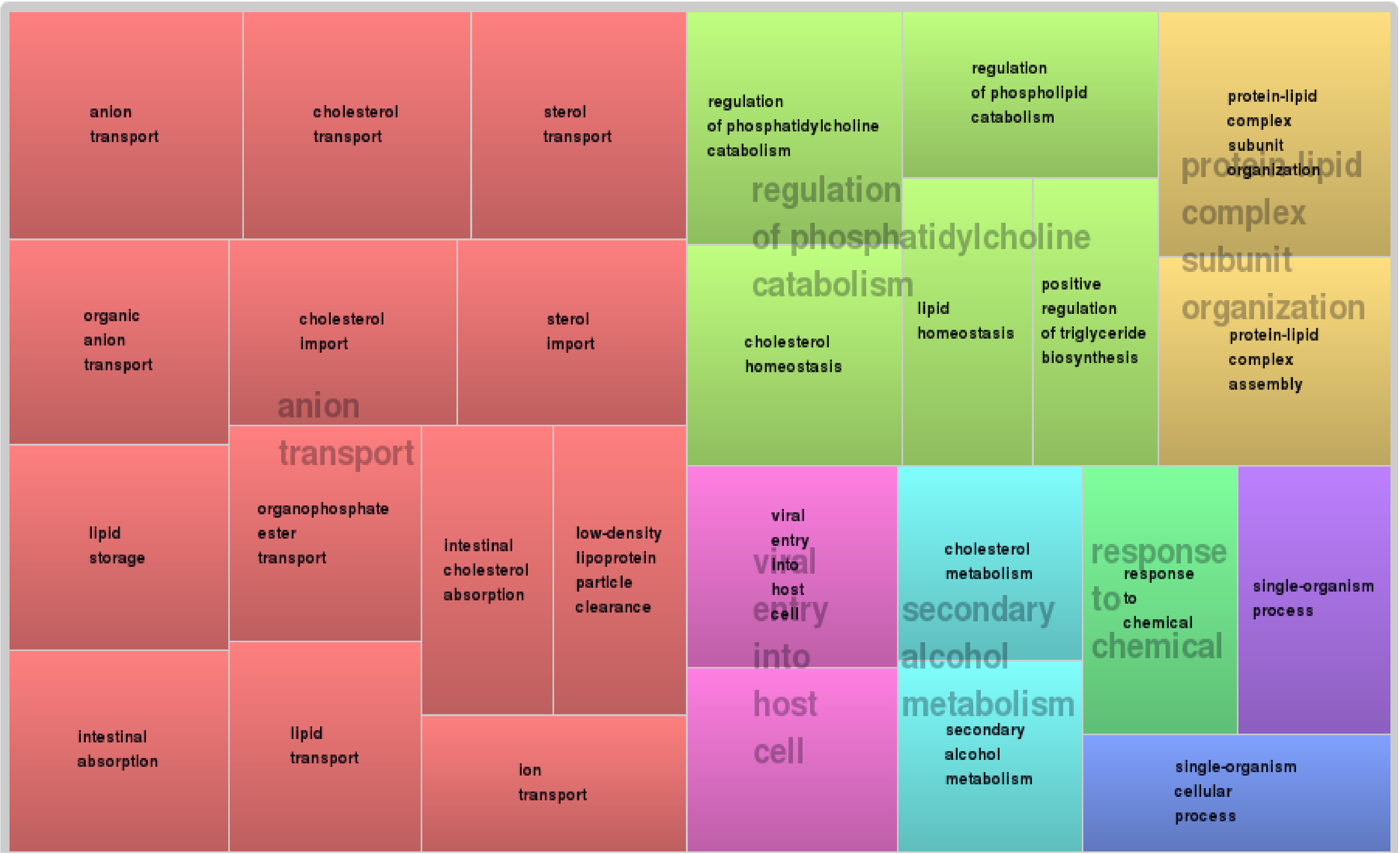


Figure M2S19 REVIGO output TreeMap of Gene Ontology Molecular Function terms over-represented by genes significantly increased in expression in old males tissue engineered tendon (n=6) RNASeq samples. Maps are coloured by category and sized by log10 p-value

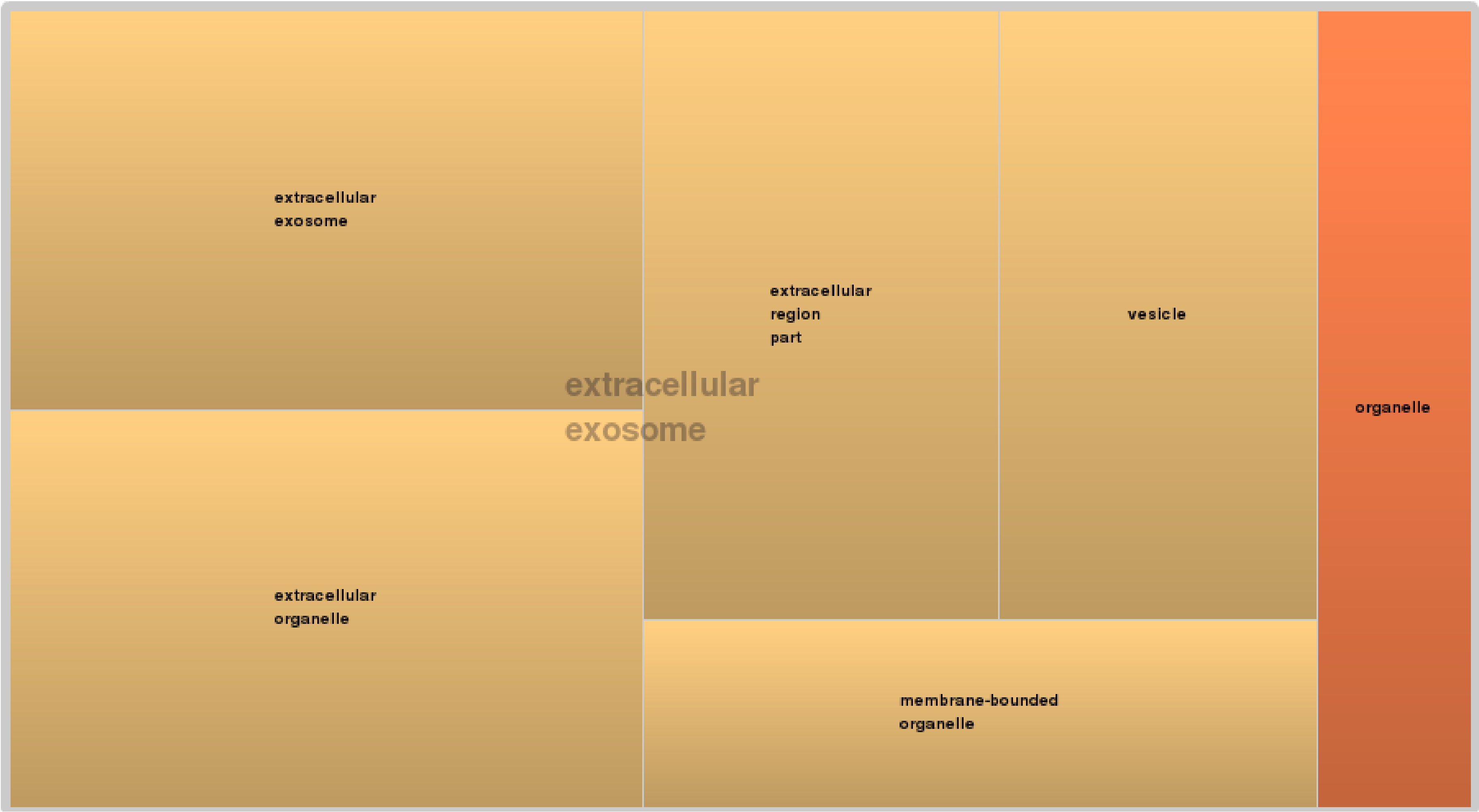


Figure M2S20 REVIGO output TreeMap of Gene Ontology Cellular Component terms over-represented by genes significantly increased in expression in old males tissue engineered tendon (n=7) RNASeq samples. Maps are coloured by category and sized by log10 p-value

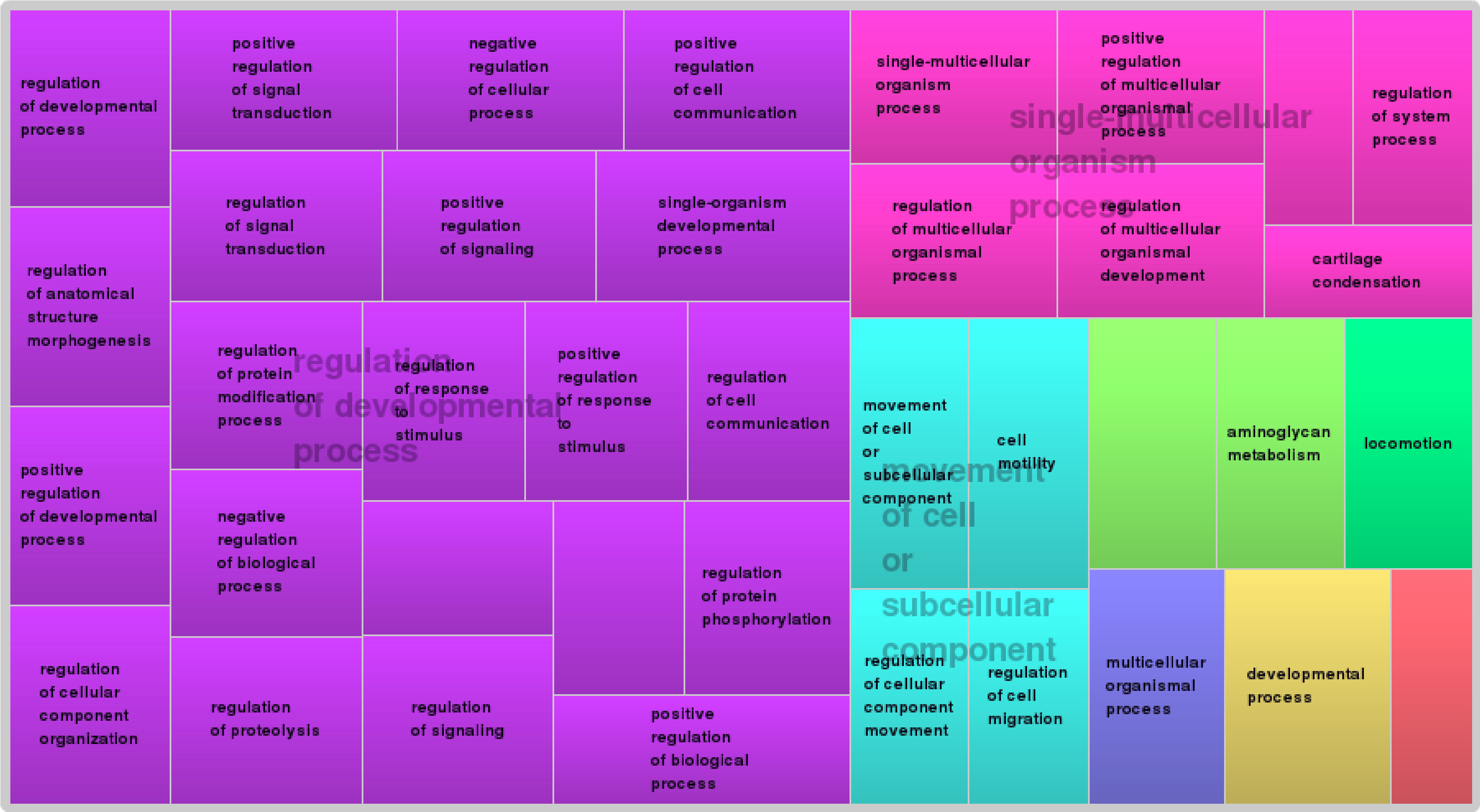


Figure M2S21 REVIGO output TreeMap of Gene Ontology Biological Process terms over-represented by genes significantly decreased in expression in old male tissue engineered tendon (n=44) RNASeq samples. Maps are coloured by category and sized by log10 p-value

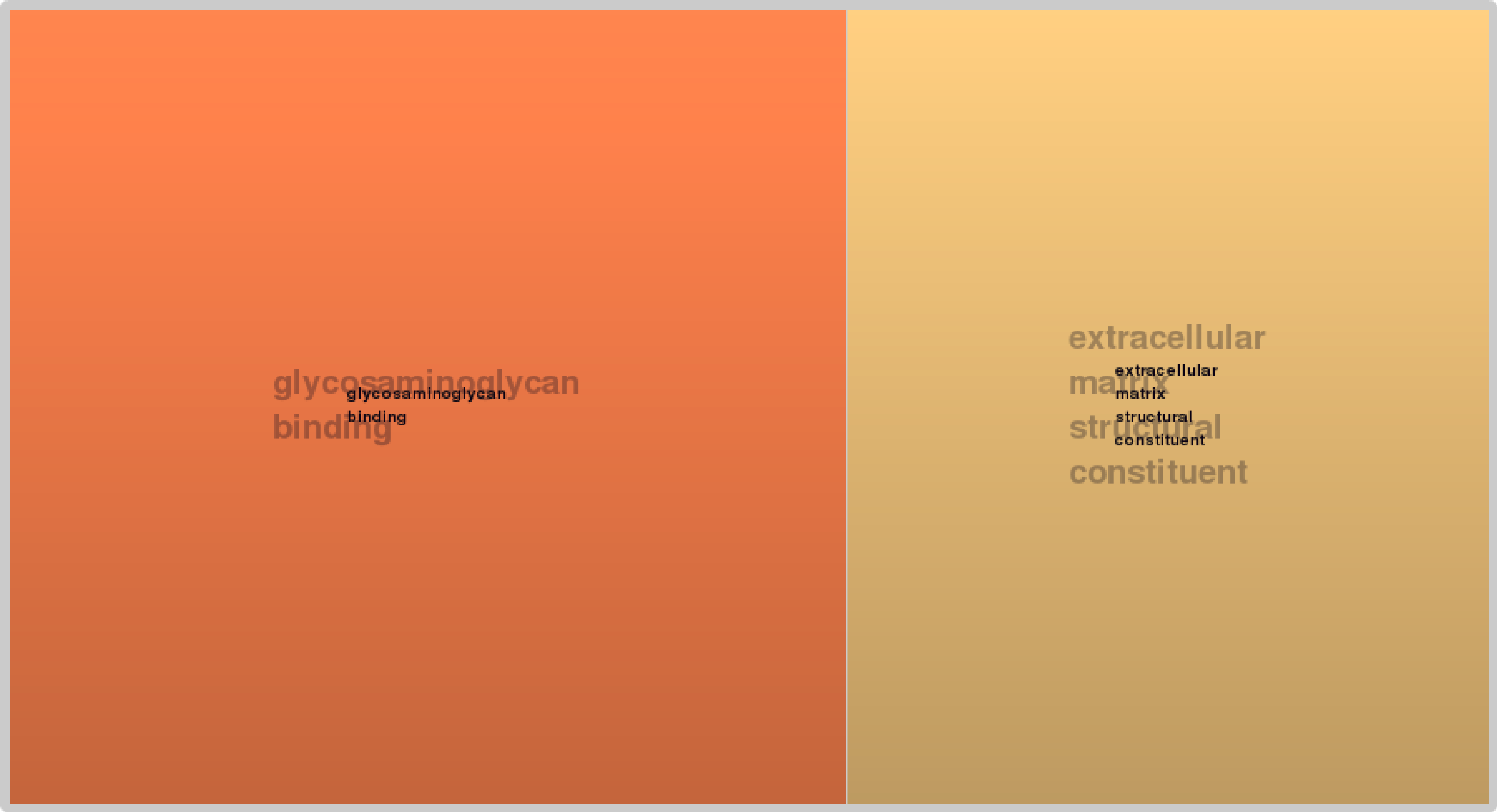


Figure M2S22 REVIGO output TreeMap of Gene Ontology Molecular Function terms over-represented by genes significantly decreased in expression in old male tissue engineered tendon (n=2) RNASeq samples. Maps are coloured by category and sized by log10 p-value

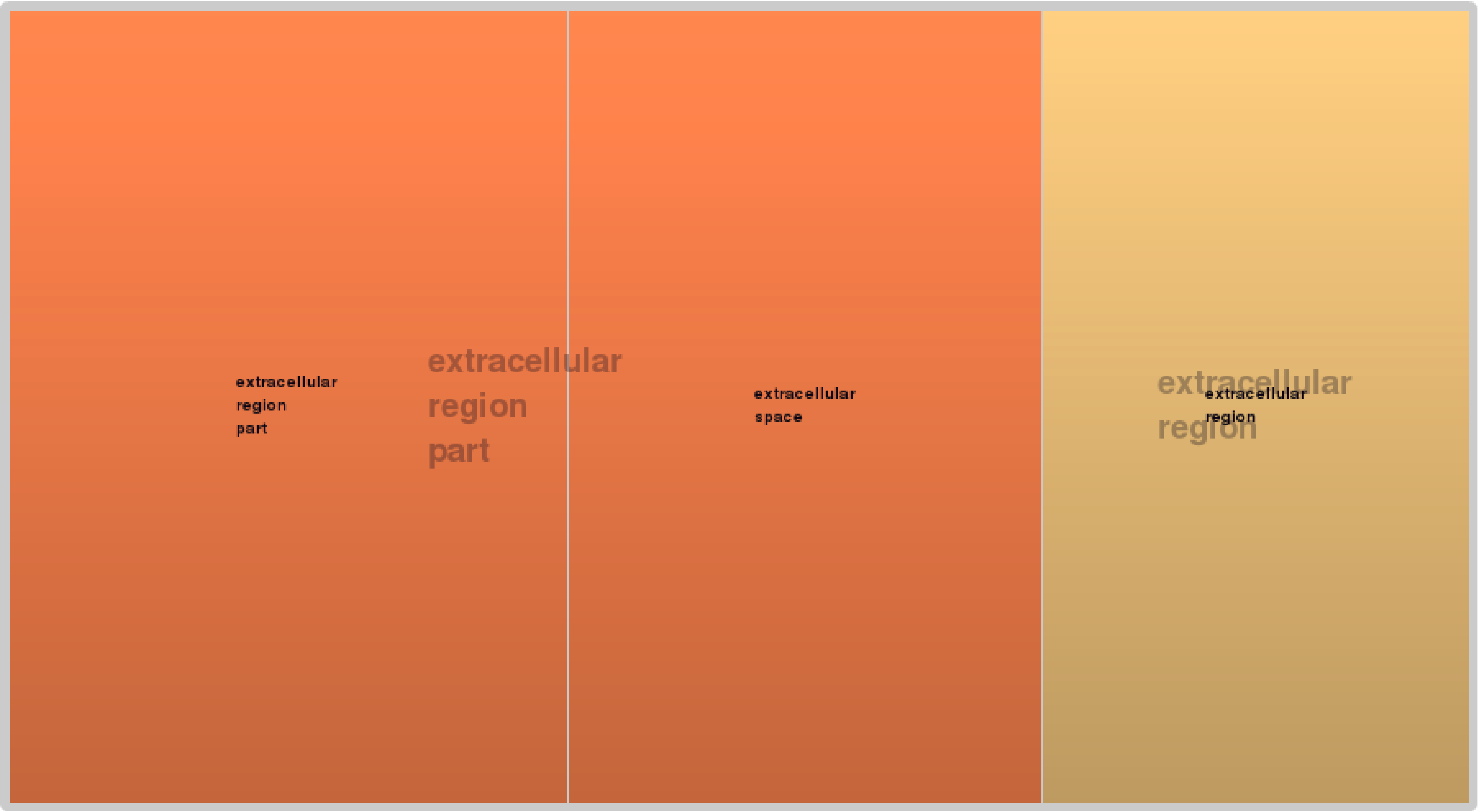


Figure M2S23 REVIGO output TreeMap of Gene Ontology Cellular Component terms over-represented by genes significantly decreased in expression in old male tissue engineered tendon (n=3) RNASeq samples. Maps are coloured by category and sized by log10 p-value

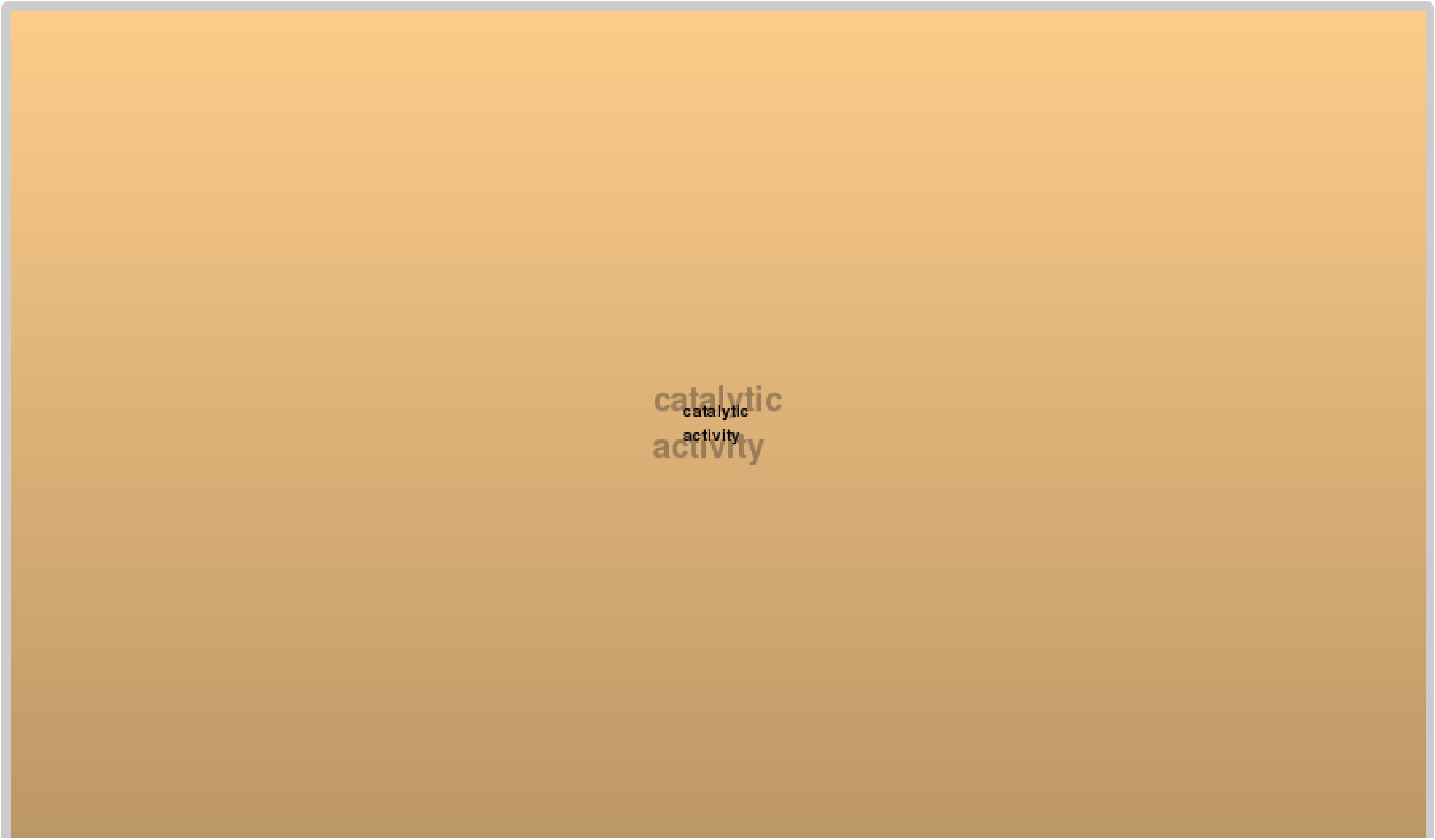


Figure M2S24 REVIGO output TreeMap of Gene Ontology Molecular Function terms over-represented by genes significantly increased in expression in old male Achilles tendon (n=1) RNASeq samples. Maps are coloured by category and sized by log10 p-value

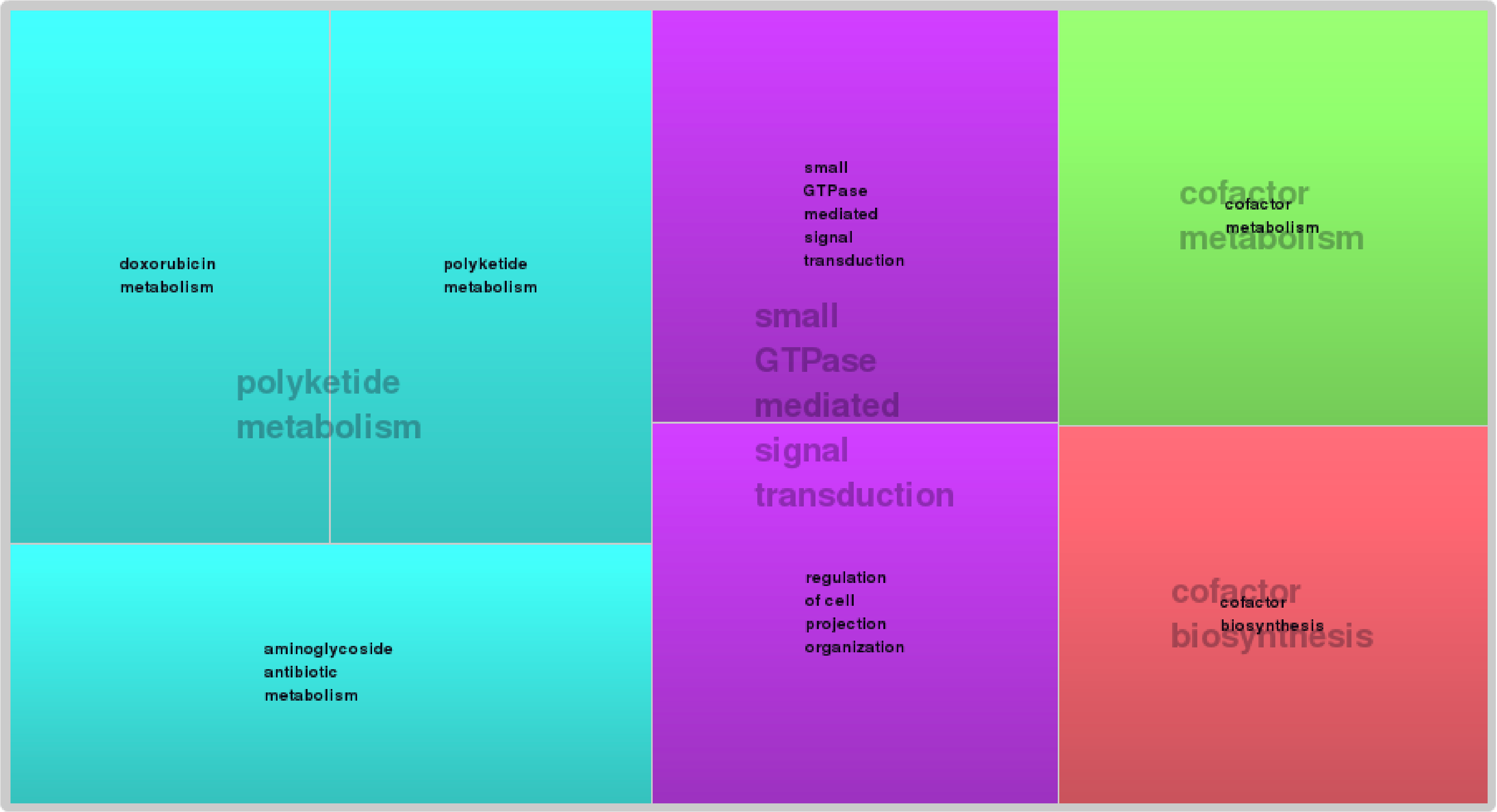


Figure M2S25 REVIGO output TreeMap of Gene Ontology Biological Process terms over-represented by genes significantly decreased in expression in old male Achilles tendon (n=8) RNASeq samples. Maps are coloured by category and sized by log10 p-value

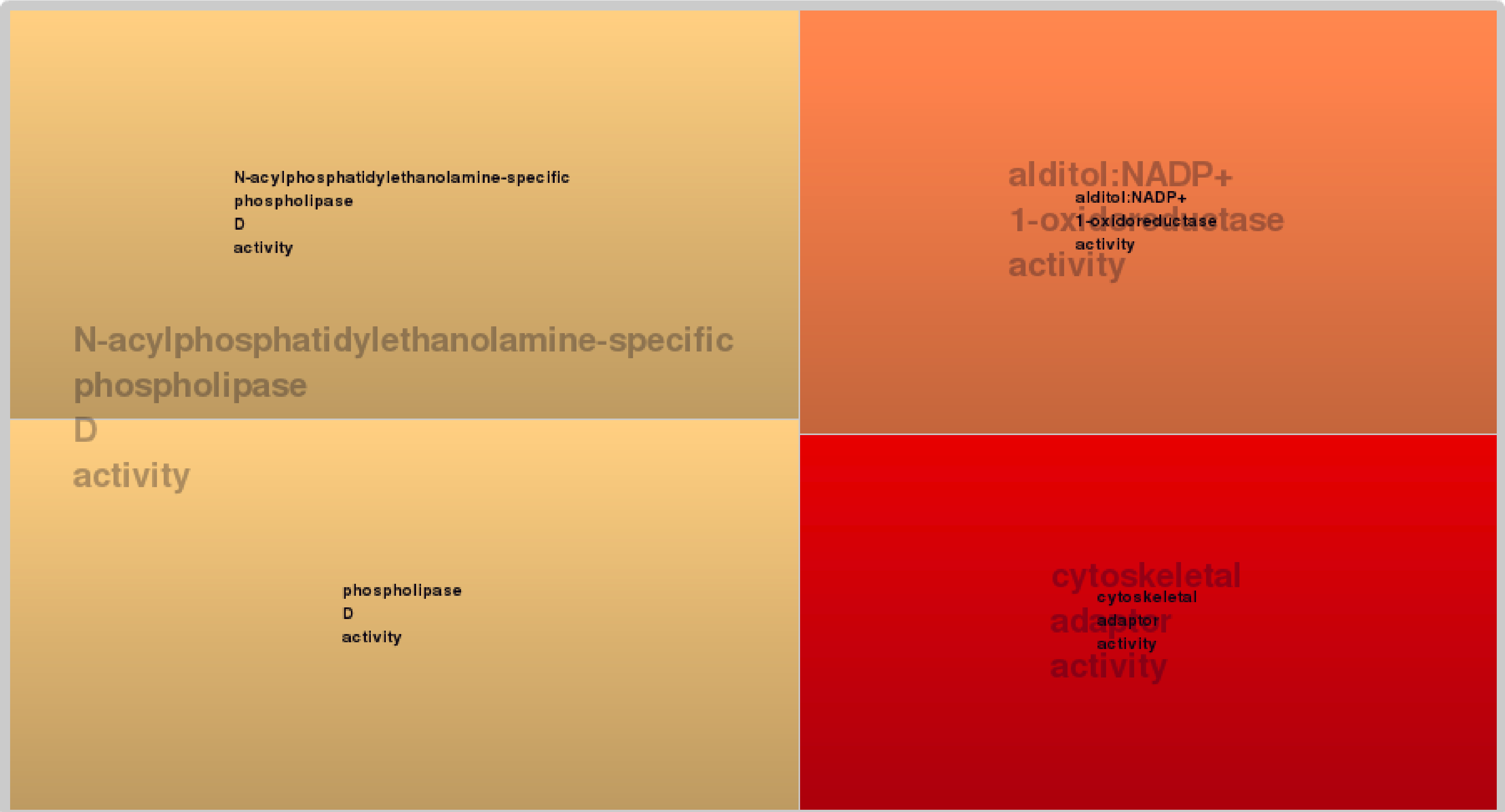


Figure M2S26 REVIGO output TreeMap of Gene Ontology Molecular Function terms over-represented by genes significantly decreased in expression in old male Achilles tendon (n=4) RNASeq samples. Maps are coloured by category and sized by log10 p-value

# Cross platform analysis of transcriptomic data identifies ageing has distinct and opposite effects on tendon in males and females

**Louise. I. Pease[1], Peter. D. Clegg[1,2], Carole. J. Proctor[1,3], Daryl. J. Shanley[1,4], Simon. J. Cockell[5], and Mandy. J. Peffers*[1,2]**

[1]MRC – Arthritis Research UK Centre for Integrated research into Musculoskeletal Ageing (CIMA)
[2*]Department of Musculoskeletal Biology, Institute of Ageing and Chronic Disease, The University of Liverpool, Leahurst Campus, Neston. CH64 7TE, UK
[3]Institute of Cellular Medicine, Newcastle University, NE2 4HH, UK
[4]Institute for Cell and Molecular Biosciences, Newcastle University, NE1 7RU, UK
[5]Faculty of Medical Sciences, Bioinformatics Support Unit, Framlington Place, Newcastle University, Newcastle, NE2 4HH
[*]M.J.Peffers@liverpool.ac.uk

## ABSTRACT

The development of tendinopathy is influenced by a variety of factors including age, gender, sex hormones and diabetes status. Cross platform comparative analysis of transcriptomic data elucidated the connections between these entities in the context of ageing. Tissue-engineered tendons differentiated from bone marrow derived mesenchymal stem cells from young (20 – 24 years) and old (54 – 70 years) donors were assayed using ribonucleic acid sequencing (RNA-seq). Extension of the experiment to microarray and RNA-seq data from tendon identified gender specific gene expression highlighting disparity with existing literature and published pathways. The results identify that in old males decreased expression of CRABP2 leads to cell proliferation, whereas in old females it leads to cellular senescence and an increase in autophagy. In conjunction with existing literature the results explain gender disparity in the development and types of degenerative diseases as well as highlighting a wide range of considerations for the analysis of transcriptomic data. Wider implications are that degenerative diseases may need to be treated differently in males and females because alternative mechanisms may be involved.

## Statistical Discussion

The aim of a transcriptomic experiment is to minimise variation introduced by experimental methods and control for compounding effects to increase the utility of results[1]. Available samples and their age attributes (Table 12) highlight comparability issues with RNA-seq donors being younger than those used in the microarray experiment in the young age group. In addition there were proportionally more young than old males assayed in the microarray experiment.

Incomplete recording of phenotypic data on Array Express limited the number of samples available for analysis. The number of replicates needed to gain accurate results from an RNA-seq experiment is one for debate. The general consensus is more is always better. Schurch *et al*.[2] investigated the number of replicates required in an RNA-seq experiment using *Saccharomyces cerevisiae* BY4741 sequenced using illumina HiSeq 2000 with seven technical replicates per biological replicate processed in four batches. The results were analysed using a variety of algorithms and tools. CuffDiff with three biological replicates produced false positive results consistent with zero and successfully captured differential expression consistent with other analysis methods. However the authors did not recommend CuffDiff for analysis of RNA-seq data whereas we do for the reasons outlined in the following section.

In the former study[2] *S. cerevisiae* was cultured in yeast extract peptone-dextrose + adenine (YPAD). Yeast extract is neither chemically defined nor consistent between batches and so avoidable experimental and environmental variation was introduced. Previous work has identified that significant pH changes occur during culture of *S. cerevisiae* in yeast extract based media which can contribute to variation[3]. The main aim of Schurch *et al*.'s study was to ascertain the most reliable RNA-seq data processing protocols. Table S13 contrasts experimental and data analysis methods used by[2] with those used in this study.

Table S13. Comparison of methods used by[2] and in this study

| Technology | Read type | Alignment Parameterisation | Batches | Samples | Lanes |
|---|---|---|---|---|---|
| Illumina HiSeq 2000[2] | single | standard | 4 | 96 | 7 |
| Illumina HiSeq 2000 (current) | paired | sample specific | 2 | 15 | 1 |

The overview in Table S13 identifies[2] used error prone single end reads, read alignment used standard parameterisation values for all samples, and samples were processed in four batches of seven lanes. Increased statistical power was achieved in this study by using alignment parameterisation values calculated for each sample. Samples were processed in one lane in one batch per experiment (n=2).[2] also assessed tools by running 100 bootstrap iterations on repeated sub-selections of samples. An exception to this was CuffDiff where due to slow processing of data the number of iterations was reduced to 30. The validity of the conclusions on CuffDiffs performance was therefore dependent on: variation introduced by experimental methods, values used for alignment parameters, sample selection, bias resulting from batch effects. Additionally CuffDiff was subject to a higher probability of encountering batch effects and variability as a consequence of fewer iterations. It is the opinion of the authors that the methods used in the former study[2] undermined the power of CuffDiff in the detection of differential expression.[2] concluded CuffDiff produced zero false positives at a low fold change with as few as three replicates, but it did not faithfully measure differential gene expression. This conclusion may be in part due to the use of fold changes estimated from $\log_2$ transformed data, $\log_2$ transformation of data following a negative binomial distribution (Figure 2) leads to a reduction in accuracy[4]. CuffDiff is a powerful algorithm that can estimate expression at the transcript level (which is more accurate, but takes longer) and controls for variability across replicate libraries[5]. This control over variability is subject to conditions 1 and 2 laid out by[6]. Subsequently a significant reduction in sensitivity and significance occurs when: samples are not grouped biologically, experimental methods increase variation, extensive batch effects are introduced, sample specific alignment parameters are not calculated to inform alignments. What is more in accordance with the data distribution observed in this study it could be hypothesised the assumptions of some of the algorithms used in the study by Schurch *et al*. may not be suited to analysing RNA-seq data. In this study biologically defined grouping of samples by age and gender altered data distribution estimates and increased statistical power. This shows the similarity of replicates within a group is as important as the number of replicates available.

Sample grouping prior to CuffDiff analysis has allowed for significance calculations based on the correct data distributions for each group and this has increased statistical power. The power to detect significant differences in sample groups also depends on closeness of means within test groups, this ultimately determines the variance. Significant results are obtained when the means are close and variance is low. Accuracy can be increased and variance reduced by increasing the number of samples in each group. In cases where the number of samples are low; such as in this study, better defining sample groups so that each sample within the group contributes less to the variance can greatly increase significance[6]. The conditions outlined by Glass *et al*[6] are defined as:

1. When n's are unequal and variances are heterogeneous, the actual significance level may greatly exceed the nominal significance level when samples with smaller n's come from populations with larger variances.

2. When n's are unequal and variances are heterogeneous, the actual significance level may be greatly exceeded by the nominal significance level when samples with smaller n's come from populations with smaller variances.

This study shows that analysing data without gender or age separation identifies no differentially expressed genes. Gender and age separation identified both age and gender affected genes (Tables M1S2 and M1S3). Defining sample groups by their biological attributes improved the accuracy of data distribution, mean and variance calculations; leading to the identification of more differentially expressed genes in each group. The power to detect differential expression was significantly increased in this study by manipulation of principles 1 and 2 outlined above[6]. Biologically informed grouping and sample group balancing ensured n's were equal, and variances were small and homogeneous within sample groups, generating the principle:

- When n's are equal and variances are homogeneous, the actual significance level may greatly exceed the nominal significance level when samples with smaller n's come from populations with smaller variances.

Previous assessment of age and gender impacts using the current data (E-GEOD-26051)[7] has been completed using covariance; identifying that both age and gender only contributed to approximately one percent of variance.

Covariance is widely used to assess the impact of experimental factors. The method relies on the calculation of a mean value for parameters (age or gender). Separating samples by compounding factors in this study identified that the combination of age and gender have opposing effects on gene expression. Consequently it could be concluded that the failure to identify these impacts in the previous study[7] was due to samples not being separated by compounding factors contributing to increased variance heterogeneity in age and gender groups as well as zeroing the calculated means. It appears that highly significant changes in gene expression in females were masked by the inclusion of a greater number of males, increased variance due to mixed gender analysis, and application of the wrong statistical tests and assumptions. Jelinsky *et al*.[7] violated conditions 1 and

2 defined by Glass *et al*.[6]. Although ANCOVA works when these assumptions are violated it assumes homogeneity of variance, an assumption that was not met. ANCOVA is also subject to an exception when there are an unequal number of subjects in groups (Table 12). In accordance with recorded variance in this study it could be concluded that the imbalanced experimental design (young males (n=11 low variance), old males (n=4 high variance)) in the microarray study contributed to the reduction in differentially expressed genes identified in males in accordance with the rule:

- When n's are unequal and variances are homogeneous, the actual significance level may fall well below the nominal significance level when samples with smaller n's come from populations with higher variances.

This rule highlights an additional factor that may have determined the number of male genes identified as differentially expressed in this study. Table 12 shows that there were 11 young males in the microarray study and only four old males, therefore giving rise to unequal n's and higher variance. The impact of unbalanced sample groups was tested by balancing male microarray samples into groups of young 35-50 (n=3) and old $< 59$ (n=3). Sample balancing identified only one gene (Table M1S1) a potassium gated voltage channel (KCNJ16) that was three fold decreased in expression. Balancing male age groups results in a reduction in sample group size further reducing the number of genes identified as differentially expressed in males. This may be a consequence of higher variance in old males.

## Hormones, lipids and the cell cycle

Hormones have been implicated in regulating oxidative stress and cell cycle processes; testosterone increases, but oestrogen decreases the production of red blood cells, DNA, RNA, and proteins[8]. Genes and pathways increased in old male TET are known to respond to steroid concentrations as well as the availability of lipids. Pathway changes represent alterations in lipid metabolism and profiles. Lipid profiles have been reported to be affected by a range of factors. Testosterone replacement reduces LDL and cholesterol, increases glucose oxidation, reduces insulin resistance and increases lipolysis[9]. Testosterone and oestrogen have both been implicated in regulating lipid profiles, and increasing the activity of the plasma membrane calcium pump[9–13]. Post-menopausal women have significantly lower testosterone concentrations than menstruating women, however in males testosterone concentrations remain stable until approximately 63 years of age[14]. Therefore gender and age related differences in lipid profiles and metabolism identified in this study may be a consequence of hormone differences. Interestingly genes involved in androgen receptor activity were decreased in expression in males (Figure M2S12), and two genes involved in responding to hormones (SGK1 Serum/Glucocorticoid Regulated Kinase 1, RAN, Member RAS Oncogene Family, Androgen Receptor-Associated Protein 24) were identified by network analysis on genes increased in expression in tissue engineered tendon (Figure M2S17). Immune signalling was affected by ageing in males and females, notably increases in cytokine-cytokine receptor signalling, natrual killer cells and inteleukin mediated signalling in males were mirrored by decreases in females. Immune responses are known to differ in males and female with females having stronger responses and higher concentrations of immunoglobulin, which has been linked to hormones[15].

Biosynthesis of testosterone, and oestrogen rely on the enzyme DHEA and availability of hydroxycholesterol; also a precursor for *de novo* vitamin D synthesis. A decline in vitamin D concentration occurs in old age and is a risk factor for the development of autoimmune disorders, infections, type 2 diabetes, multiple sclerosis and rheumatoid arthritis[16]. Calcium and vitamin D supplements alter the lipid profiles of diabetics. Vitamin D is thought to regulate *de novo* lipid synthesis, inflammation and calcium uptake[9]. A reduction in cholesterol synthesis or alterations in the oxidative state of cholesterol in old age could underpin reductions in hormone and vitamin D synthesis. This scenario is supported by observed differences in the efficacy of testosterone replacement that are thought to be due to the potential for cells to convert testosterone to dihydroxytestosterone[9]. Table M1S5 shows the enzyme 7-dehydrocholesterol reductase is decreased in expression in old females, thereby reducing availability of hydroxycholesterol. Additionally a significant over-representation of genes involved in cholesterol biosynthesis was identified by genes decreased in expression in old females (Table 6). In females Vascular Endothelial Growth Factor (VEGF) expression is significantly reduced (Table M1S5). Vitamin D3 is thought to increase VEGF expression[17], therefore reduced VEGF expression in females may be reflective of a vitamin D deficient state. Contrary to this TGF-*Beta* mediated growth inhibition is thought to be induced by treatment with vitamin D3[18,19]. In females TGF-*Beta* was increased in expression coinciding with reductions in cell cycle, however in males it was decreased in expression coinciding with high cell cycle. This may be suggestive of opposing effects of vitamin D in males and females, and confusion may have arisen through invalid comparisons of males and females. Determined relationships between the entities discussed are summarised in Figure 1.

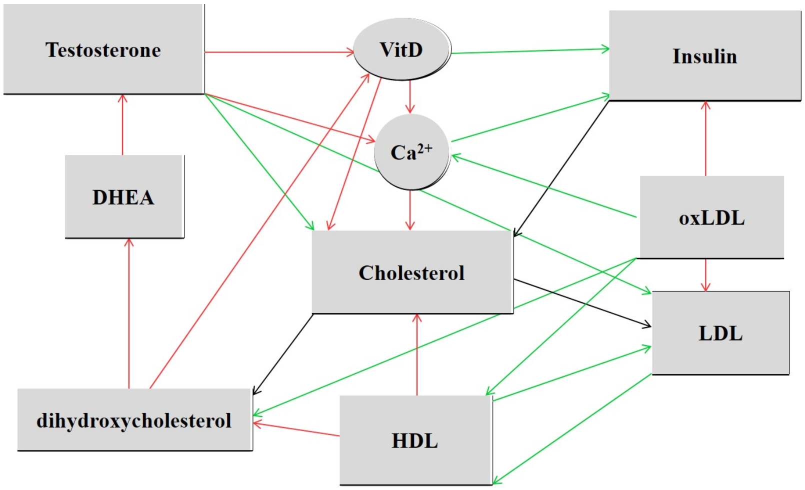


**Figure 1.** Entity relation diagram summarising the interactions between Vitamin D, calcium, insulin and cholesterol described above. Red arrows show positive regulatory processes thereby increasing entity concentrations, green arrows show negative regulatory processes thereby decreasing entity concentrations. Where the relationship is not fully determined entities are linked by black arrows.

High Density Lipoproteins (HDLs) regulate cholesterol bioavailability, and altered HDL profiles have been found to be associated with immune mediated disorders including rheumatoid arthritis, SLE, Crohn's disease and multiple sclerosis[20]. HDLs are decreased in type 2 diabetes which has been associated with small dense oxidized and glycated LDLs. HDLs are important components of MHCs, in this study MHCII expression was decreased in old males but was increased in old females alongside ubiquitin mediated proteolysis (Table M1S5), reported to promote the production of inflammatory cytokines resulting in endocytosis of MHCIIs[21]. Oxidised LDLs are associated with dysregulation of calcium homeostasis, ER stress and increased autophagy[22,23]. Increased lipolysis, insulin resistance and proteolysis were seen in old females providing evidence of autophagy that may also be reflective of an increase in oxidized LDLs and alterations in calcium homeostasis.

Retinoic acid binding protein CRABP2 was identified as decreased in expression with age in males and females using RNA-seq. CRABP2 is thought to influence tumor growth by; activation of RARs leading to cell cycle arrest, and activation of PPARs leading to proliferation. The outcome is determined by testosterone which inhibits RARs and FABP5 that has highest expression levels in females. FABP5 influences lipid metabolism and oestrogen receptor activity. Expression of FABP5 is also lowered by dietary intake of poly-unsaturated fatty acids (PUFAs)[24–27], suggesting the availability of PUFAs regulate FABP5 expression. Retinoic acid deficiency in rats has been shown to result in feminisation of gene expression and reduction in DHEA expression[28]. Since cholesterol is unaffected[28] retinoic acid could be said to regulate testosterone synthesis through DHEA. Retinoic acid responsive pathways regulate lipid and hormone synthesis as well as auto-immune disorders and the development of tumors. PPAR isoforms are differentially expressed in males (PPAR-$Alpha$) and females (PPAR-$Beta$) and this has been linked to seasonal alterations in testosterone and oestrogen concentrations, lipid metabolism and alterations in oxidative stress[29–34]; all of which differ in males and females. In this study PPAR signalling was increased, and RAR$alpha$ decreased in old male tissue engineered tendon (Table M2S5).

Hormones influence oxidative stress levels; progesterone increases oxidative stress while oestrogen suppresses the activity of NADPH oxidase. Oestrogen is reported to reduce the expression of oxidative stress protein encoding genes SOD2 and SOD3. There is a tendency for the source of oxidative stress in males to be mitochondrial, based on measurement of mitochondrial SOD proteins, whereas in females there is a greater contribution from the endoplasmic reticulum (ER)[35–38]. High oxidative stress has been linked to reduced lifespan; interestingly higher oxidative stress has been identified in female C57BL6 mice; one of few species where females have a shorter lifespan[35]. Lower NADPH oxidase concentration have been identified in female rats[39]. Oestrogen and progesterone have been implicated because ovariectomy of female rats abolished gender differences in oxidative stress. In humans there have been very few studies exploring the role of gender and oxidative stress. Frisard *et al.*[40] investigated resting metabolic rate (RMR) in humans to determine its impact on oxidative stress levels in the young, old, and those $>$ 90yrs. They collected data on; age, weight, height, BMI, percentage fat, Free Fat Mass (FFM), and Fat Mass (FM), measuring RMR following overnight fasting using $VO_2$ max. Significant ($p<0.001$) differences in weight, percentage fat, FFM and RMR for males and females were observed so RMR was adjusted for FFM, FM and gender. The study reported oxidative stress levels as one value per age group and the authors concluded there was no correlation between RMR and oxidative stress, or lower oxidative stress in those aged $>$ 90. In a further study[41] the same authors identified and then corrected for gender

differences instead of actively studying them. As a consequence the fact that males have higher metabolic rates and higher oxidative stress were not identified or reported. The aim of their work was to identify if people aged over 90 years had lower oxidative stress levels but analysis errors meant this remains undetermined.

In males a higher cell cycle is observed alongside decreased expression of MHCII which could lead to destruction of cells by phagocytes in males (increases in fc$\gamma$ R-mediated phagocytosis). This could be driving increases in cell cycle due to the requirement for replacement cells. In females lower metabolic rates could reduce the availability of lipids leading to reductions in hormone and vitamin D concentrations in old age, thereby driving a reduction in the cell cycle. Lower cell cycle in females would explain lower variation and the propensity to develop tendinopathy and other degenerative diseases could be due to damaged cells not being replaced. There are key gender differences in responses to retinoic acid and vitamin D. Retinoic acid appears to regulate testosterone synthesis and promotes the cell cycle, high doses of vitamin D in females counteract this effect[42].

## References


1. Novak, J. P., Sladek, R. & Hudson, T. J. Characterization of variability in large-scale gene expression data: Implications for study design. *Genomics* **79**, 104–113 (2002). URL <GotoISI>://WOS:000173296600014.

2. Schurch, N. J. *et al.* How many biological replicates are needed in an rna-seq experiment and which differential expression tool should you use? *Rna* **22**, 839–851 (2016). URL <GotoISI>://WOS:000376205600004.

3. Pease, L. I. Toxicogenomics : a transcriptomics approach to assess the toxicity of 4-nitrophenol to *sachharomyces cerevisiae*. *thesis* 32–54 (2011). URL http://hdl.handle.net/10443/1142.

4. O'Hara, R. B. & Kotze, D. J. Do not log-transform count data. *Methods Ecol. Evol.* **1**, 118–122 (2010). URL <GotoISI>://WOS:000288914100003.

5. Anjum, A. *et al.* Identification of differentially expressed genes in rna-seq data of arabidopsis thaliana: A compound distribution approach. *J. Comput. Biol.* **23**, 239–247 (2016). URL <GotoISI>://WOS:000373278500002.

6. Glass, G. V., Peckham, P. D. & Sanders, J. R. Consequences of failure to meet assumptions underlying fixed effects analyses of variance and covariance. *Rev. Educ. Res.* **42**, 237–288 (1972). URL <GotoISI>://WOS:A1972N995700001.

7. Jelinsky, S. A. *et al.* Regulation of gene expression in human tendinopathy. *Bmc Musculoskelet. Disord.* **12** (2011). URL <GotoISI>://WOS:000290616400001.

8. Yamamoto, M., Hayashi, Y. & Yamada, K. Sex hormones and bone marrow functions. *Nihon rinsho. Jpn. journal clinical medicine* **32**, 3346–9 (1974). URL <GotoISI>://MEDLINE:4615186.

9. Kelly, D. M. & Jones, T. H. Testosterone: a metabolic hormone in health and disease. *J. Endocrinol.* **217**, R25–R45 (2013). URL <GotoISI>://WOS:000319457600002.

10. Slusher, A. L., McAllister, M. J. & Huang, C.-J. A therapeutic role for vitamin d on obesity-associated inflammation and weight-loss intervention. *Inflamm. research : official journal Eur. Histamine Res. Soc.* **64**, 565–75 (2015). URL <GotoISI>://MEDLINE:26142253.

11. Asemi, Z. *et al.* Calcium plus vitamin d supplementation affects glucose metabolism and lipid concentrations in overweight and obese vitamin d deficient women with polycystic ovary syndrome. *Clin. Nutr.* **34**, 586–592 (2015). URL <GotoISI>://WOS:000357241900005.

12. Tabesh, M., Azadbakht, L., Faghihimani, E., Tabesh, M. & Esmaillzadeh, A. Effects of calcium-vitamin d co-supplementation on metabolic profiles in vitamin d insufficient people with type 2 diabetes: a randomised controlled clinical trial. *Diabetol.* **57**, 2038–2047 (2014). URL <GotoISI>://WOS:000341708900005.

13. Dick, I. M., Prince, R. L., Kelly, J. J. & Ho, K. K. Y. Estrogen effects on calcitriol levels in postmenopausal women - a comparison of oral versus transdermal administration. *Clin. Endocrinol.* **43**, 219–224 (1995). URL <GotoISI>://WOS:A1995RM99400012.

14. Yamaguchi, A., Ichimura, T. & Yamabe, T. The measurement of plasma-free testosterone in normal menstrual females, pregnant females, post menopausal females and vulvar dystrophy. *Nihon Naibunpi Gakkai zasshi* **64**, 482–8 (1988). URL <GotoISI>://MEDLINE:3208920.

15. Marshall-Gradisnik, S., Green, R., Brenu, E. & Weatherby, R. Anabolic androgenic steroids effects on the immune system: a review. *Cent. Eur. J. Biol.* **4**, 19–33 (2009). URL <GotoISI>://WOS:000263003200003. DOI 10.2478/s11535-008-0058-x.

**16.** Cutolo, M. *et al.* Vitamin d, steroid hormones, and autoimmunity. *Steroids Neuroendocr. Immunol. Ther. Rheum. Dis. I* **1317**, 39–46 (2014). URL <GotoISI>://WOS:000337808900007.

**17.** Cardus, A. *et al.* 1,25-dihydroxyvitamin d(3) regulates vegf production through a vitamin d response element in the vegf promoter. *Atheroscler.* **204**, 85–89 (2009). URL <GotoISI>://WOS:000266670100015.

**18.** Kimura, A., Ohashi, K. & Naganuma, A. Cisplatin upregulates saccharomyces cerevisiae genes involved in iron homeostasis through activation of the iron insufficiency-responsive transcription factor aft1. *J. Cell. Physiol.* **210**, 378–384 (2007). URL <GotoISI>://WOS:000242876400012.

**19.** Heger, J. *et al.* Tgf beta receptor activation enhances cardiac apoptosis via smad activation and concomitant no release. *J. Cell. Physiol.* **226**, 2683–2690 (2011). URL <GotoISI>://WOS:000294214400026.

**20.** Norata, G. D., Pirillo, A., Ammirati, E. & Catapano, A. L. Emerging role of high density lipoproteins as a player in the immune system. *Atheroscler.* **220**, 11–21 (2012). URL <GotoISI>://WOS:000298374800003.

**21.** Oh, J. & Shin, J.-S. Molecular mechanism and cellular function of mhcii ubiquitination. *Immunol. Rev.* **266**, 134–144 (2015). URL <GotoISI>://WOS:000356457000010.

**22.** Muller, C., Salvayre, R., Negre-Salvayre, A. & Vindis, C. Hdls inhibit endoplasmic reticulum stress and autophagic response induced by oxidized ldls. *Cell Death Differ.* **18**, 817–828 (2011). URL <GotoISI>://WOS:000289344200008.

**23.** Verges, B. Lipid modification in type 2 diabetes: the role of ldl and hdl. *Fundamental Clin. Pharmacol.* **23**, 681–685 (2009). URL <GotoISI>://WOS:000271631800005.

**24.** Li, Q. *et al.* Serum retinol binding protein 4 is negatively related to estrogen in chinese women with obesity: a cross-sectional study. *Lipids Heal. Dis.* **15** (2016). URL <GotoISI>://WOS:000371779300004.

**25.** Vreeland, A. C., Levi, L., Zhang, W., Berry, D. C. & Noy, N. Cellular retinoic acid-binding protein 2 inhibits tumor growth by two distinct mechanisms. *J. Biol. Chem.* **289**, 34065–34073 (2014). URL <GotoISI>://WOS:000346077600032.

**26.** Zhang, F. *et al.* Dietary fatty acids affect lipid metabolism and estrogen receptor expression in n-methyl-n-nitrosourea-induced rat mammary cancer model. *Clin. Lab.* **61**, 389–395 (2015). URL <GotoISI>://WOS:000353008800023.

**27.** Huang, H. F. S., Li, M. T., VonHagen, S., Zhang, Y. F. & Irwin, R. J. Androgen modulation of the messenger ribonucleic acid of retinoic acid receptors in the prostate, seminal vesicles, and kidney in the rat. *Endocrinol.* **138**, 553–559 (1997). URL <GotoISI>://WOS:A1997WC64600006.

**28.** McClintick, J. N. *et al.* Global effects of vitamin a deficiency on gene expression in rat liver: evidence for hypoandrogenism. *J. Nutr. Biochem.* **17**, 345–355 (2006). URL <GotoISI>://WOS:000237646300006.

**29.** Attakpa, E. S., Sezan, A. & Seri, B. Ppar-ALPHA role in obesity-diabetes in mice. *Acta Endocrinol.* **9**, 533–542 (2013). URL <GotoISI>://WOS:000327227600004.

**30.** Rosenberger, T. A., Hovda, J. T. & Peters, J. M. Targeted disruption of peroxisomal proliferator-activated receptor beta (delta) results in distinct gender differences in mouse brain phospholipid and esterified fa levels. *Lipids* **37**, 495–500 (2002). URL <GotoISI>://WOS:000175743300009.

**31.** Ibabe, A., Bilbao, E. & Cajaraville, M. P. Expression of peroxisome proliferator-activated receptors in zebrafish (danio rerio) depending on gender and developmental stage. *Histochem. Cell Biol.* **123**, 75–87 (2005). URL <GotoISI>://WOS:000227426200008.

**32.** Yaacob, N. S., Goh, K. S. K. & Norazmi, M. N. Male and female nod mice differentially express peroxisome proliferator-activated receptors and pathogenic cytokines. *Exp. Toxicol. Pathol.* **64**, 127–131 (2012). URL <GotoISI>://WOS:000299030400019.

**33.** Shirinsky, I. V. & Shirinsky, V. S. Targeting nuclear hormone receptors: Pparalpha agonists as potential disease-modifying drugs for rheumatoid arthritis. *Int. journal rheumatology* **2011**, 937843–937843 (2011). URL <GotoISI>://MEDLINE:21760804.

**34.** Batista-Pinto, C., Rocha, E., Castro, L. F. C., Rodrigues, P. & Lobo-da Cunha, A. Seasonal and gender variation of peroxisome proliferator activated receptors expression in brown trout liver. *Gen. Comp. Endocrinol.* **161**, 146–152 (2009). URL <GotoISI>://WOS:000264012300022.

**35.** Ali, S. S. *et al.* Gender differences in free radical homeostasis during aging: shorter-lived female c57bl6 mice have increased oxidative stress. *Aging Cell* **5**, 565–574 (2006). URL <GotoISI>://WOS:000242198700013.

**36.** Wang, L., Zhu, L. & Wang, C.-c. The endoplasmic reticulum sulfhydryl oxidase ero1 beta drives efficient oxidative protein folding with loose regulation. *Biochem. J.* **434**, 113–121 (2011). URL <GotoISI>://WOS:000287461000011.

**37.** Wang, J. *et al.* Derlin-1 is overexpressed in human breast carcinoma and protects cancer cells from endoplasmic reticulum stress-induced apoptosis. *Breast Cancer Res.* **10** (2008). URL <GotoISI>://WOS:000254811400014.

**38.** Mota, S. I. *et al.* Oxidative stress involving changes in nrf2 and er stress in early stages of alzheimer's disease. *Biochimica Et Biophys. Acta-Molecular Basis Dis.* **1852**, 1428–1441 (2015). URL <GotoISI>://WOS:000355715200021.

**39.** Miller, A. A., De Silva, T. M., Jackman, K. A. & Sobey, C. G. Effect of gender and sex hormones on vascular oxidative stress. *Clin. Exp. Pharmacol. Physiol.* **34**, 1037–1043 (2007). URL <GotoISI>://WOS:000248977900013.

**40.** Frisard, M. & Ravussin, E. Energy metabolism and oxidative stress - impact on the metabolic syndrome and the aging process. *Endocr.* **29**, 27–32 (2006). URL <GotoISI>://WOS:000236772000005.

**41.** Frisard, M. I. *et al.* Aging, resting metabolic rate, and oxidative damage; results from the louisiana healthy aging study. *Journals Gerontol. Ser. a-Biological Sci. Med. Sci.* **62**, 752–759 (2007). URL <GotoISI>://WOS:000253828700010.

**42.** Hetland, R. B., Alexander, J., Berg, J. P., Svendsen, C. & Paulsen, J. E. Retinol-induced intestinal tumorigenesis in min/+ mice and importance of vitamin d status. *Anticancer. Res.* **29**, 4353–4360 (2009). URL <GotoISI>://WOS:000273203300006.

## Author contributions statement

P.D.C. and M.J.P. conceived the experiment(s), M.J.P. conducted the experiment(s), L.I.P analysed the results. S.J.C monitored programs and statistics. All authors reviewed the manuscript.

## Funding

Mandy Peffers is funded through a Wellcome Trust Clinical Intermediate fellowship. This work was supported by the MRC and Arthritis Research UK as part of the MRC – Arthritis Research UK Centre for Integrated research into Musculoskeletal Ageing (CIMA).

**Competing financial interests**. The authors declare no competing financial interests.
